# Supplementary material for: Cannabinoids versus placebo for pain: A systematic review with meta-analysis and Trial Sequential Analysis
Source: PLoS One. 2023 Jan 30;18(1):e0267420. doi: 10.1371/journal.pone.0267420 (PMC9886264; doi:10.1371/journal.pone.0267420)
Supplement: S1 File — (DOCX) [file pone.0267420.s002.docx]

**Cannabinoids versus placebo for pain: a systematic review with meta-analysis and Trial Sequential Analysis**

Barakji J^1,*^, Korang SK^1^, Feinberg JB^1,2^ , Maagaard M^1,3^, Mathiesen O^3,4^, Gluud C^1,5^, Jakobsen JC^1,5^

^1^ Copenhagen Trial Unit, Centre for Clinical Intervention Research, The Capital Region, Copenhagen University Hospital ─ Rigshospitalet, Copenhagen, Denmark

^2^ Medical Department, Cardiology Section, Holbaek University Hospital, Holbaek, Denmark.

^3^ Centre for Anaesthesiological Research, Department of Anaesthesiology, Zealand University Hospital, Køge, Denmark

^4^ Department of Clinical Medicine, Copenhagen University, Copenhagen, Denmar

^5^ Department of Regional Health Research, The Faculty of Heath Sciences, University of Southern Denmark, Odense, Denmark

**Supplemental**

**1 Databases searched**

- Cochrane Central Register of Controlled Trials (CENTRAL)
- Medical Literature Analysis and Retrieval System Online (MEDLINE)
- Excerpta Medica database (EMBASE)
- Latin American and Carribean Health Sciences Literature (LILACS)
- Science Citation Index Expanded on Web of Science
- BIOSIS
- ClinicalTrials.gov
- Google Scholar
- The Turning Research into Practice (TRIP) Database
- European Medicines Agency (EMA), United States Food and Drug Administration (FDA)
- China Food and Drug Administration (CFDA)
- Medicines and Healthcare products Regulatory Agency
- The World Health Organization (WHO)

**2 Search strategy**

This was the search strategy that we used in MEDLINE and corrected to fit other databases as needed. We used a minimal search strategy to ensure that we did not miss any relevant trials.

1. exp Cannabis/

2. exp Cannabinoids/

3. (cannabi* or mari*uana or nabixmol* or dronabinol* or marinol* or nabilon* or cesamet* or hash* or hemp* or levonantradol* or anandamid* or 2-AG).mp. [mp=title, abstract, original title, name of substance word, subject heading word, floating sub-heading word, keyword heading word, organism supplementary concept word, protocol supplementary concept word, rare disease supplementary concept word, unique identifier, synonyms]

4. 1 or 2 or 3

5. exp Pain/

6. (pain* or ache* or migraine*).mp. [mp=title, abstract, original title, name of substance word, subject heading word, floating sub-heading word, keyword heading word, organism supplementary concept word, protocol supplementary concept word, rare disease supplementary concept word, unique identifier, synonyms]

7. 5 or 6

8. 4 and 7

9. (random* or blind* or placebo* or meta-analys*).mp. [mp=title, abstract, original title, name of substance word, subject heading word, floating sub-heading word, keyword heading word, organism supplementary concept word, protocol supplementary concept word, rare disease supplementary concept word, unique identifier, synonyms]

10. 8 and 9

**3 PRISMA Flow Diagram**


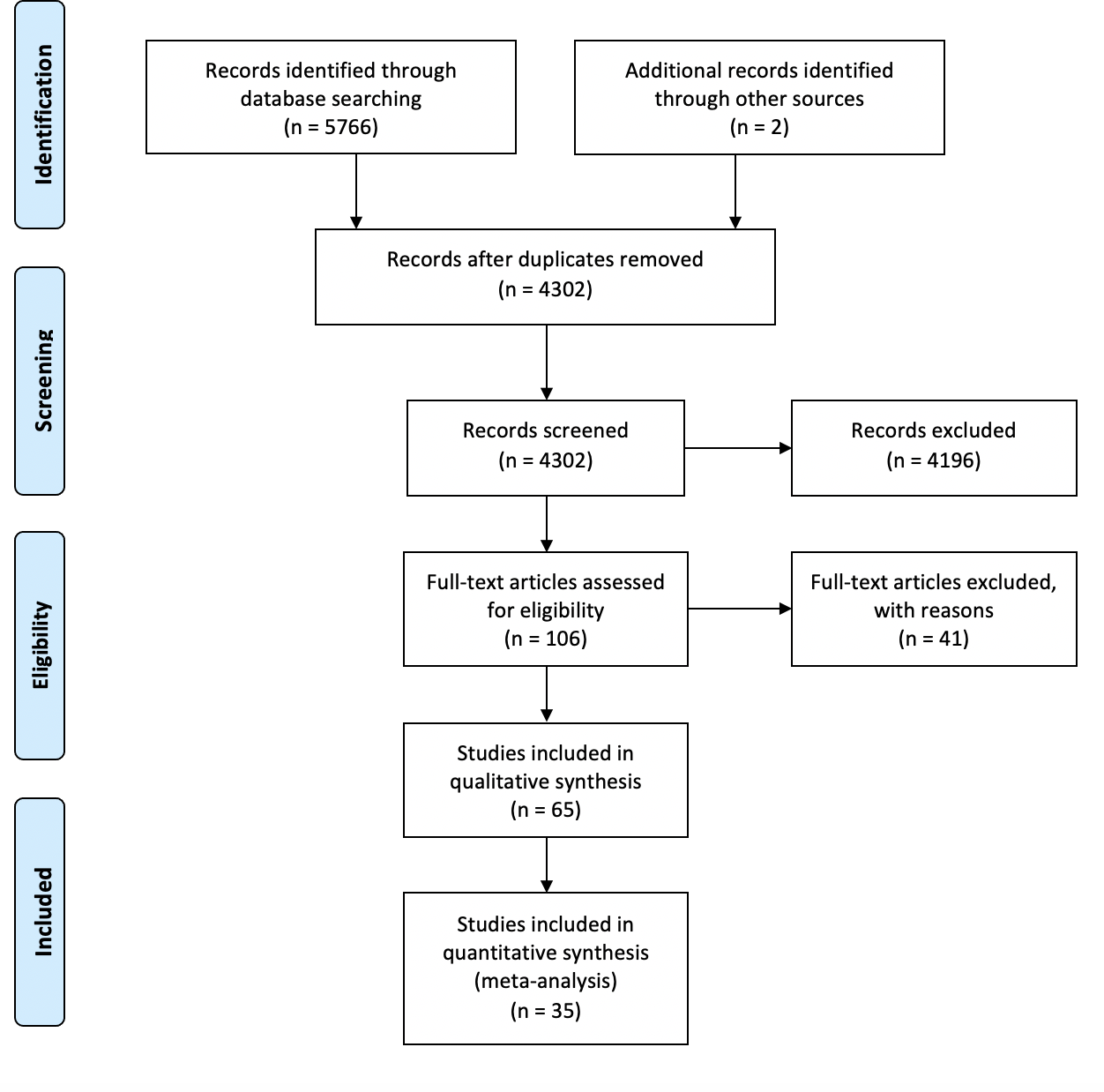


**4 Risk of bias in included studies**


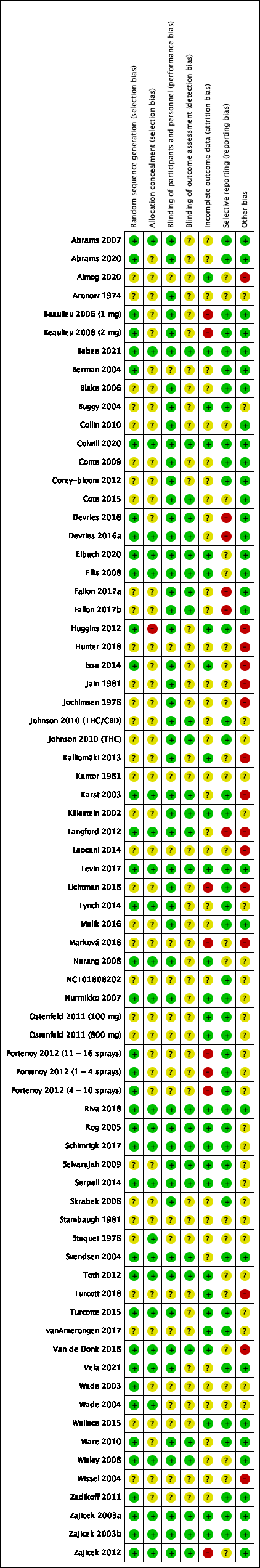


**5 All-cause mortality**


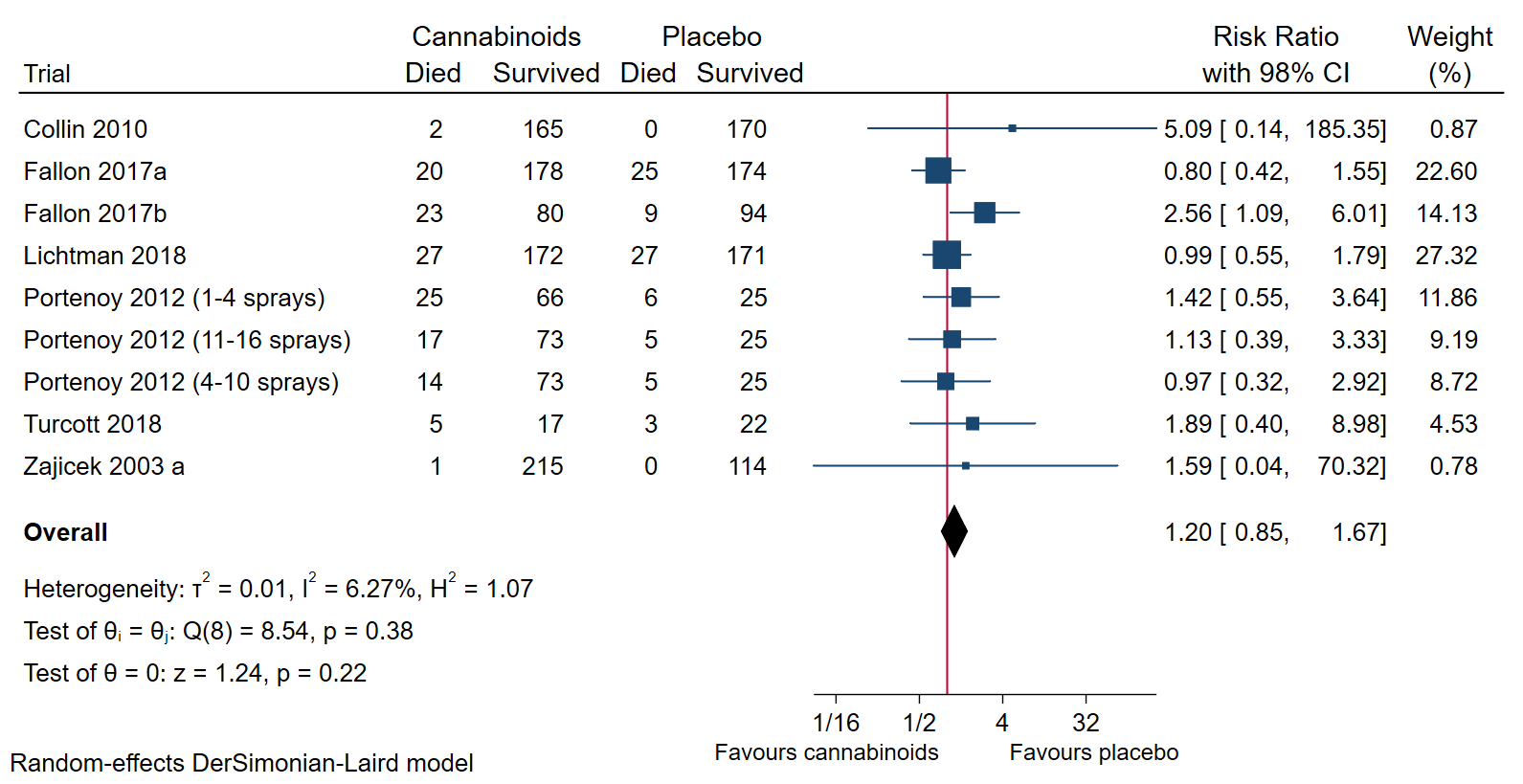


**Figure 1: Forest plot of the meta-analysis of all-cause mortality with 98% CI.** The meta-analysis showed no difference between cannabinoids versus placebo for pain on the outcome all-cause mortality.


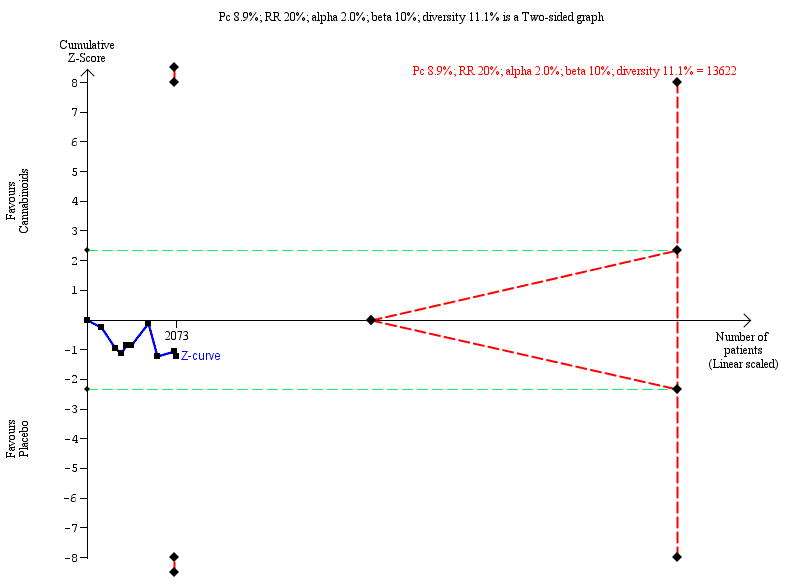


**Figure 2: Trial Sequential Analysis graph of cannabinoids versus placebo for pain on the outcome all-cause mortality using random-effects meta-analysis.** Trial Sequential Analysis showed the z-curve (the blue line) not breaching any boundary, showing that there was not enough information to confirm or reject that cannabinoids compared with placebo affected the risk of all-cause mortality by 20%.


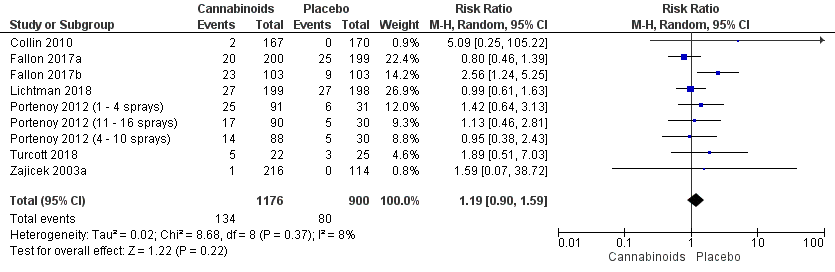


Figure 3: All-cause mortality best-worst case scenario


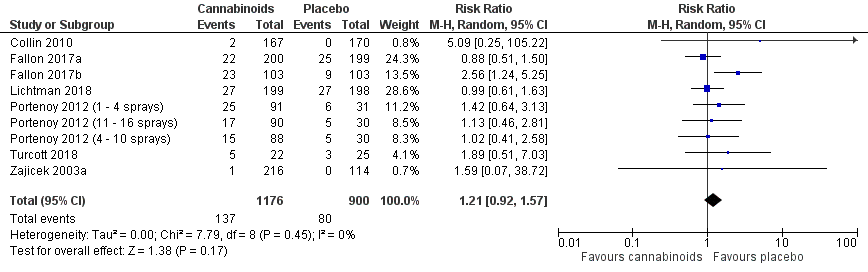


Figure 4: All-cause mortality worst-best case scenario


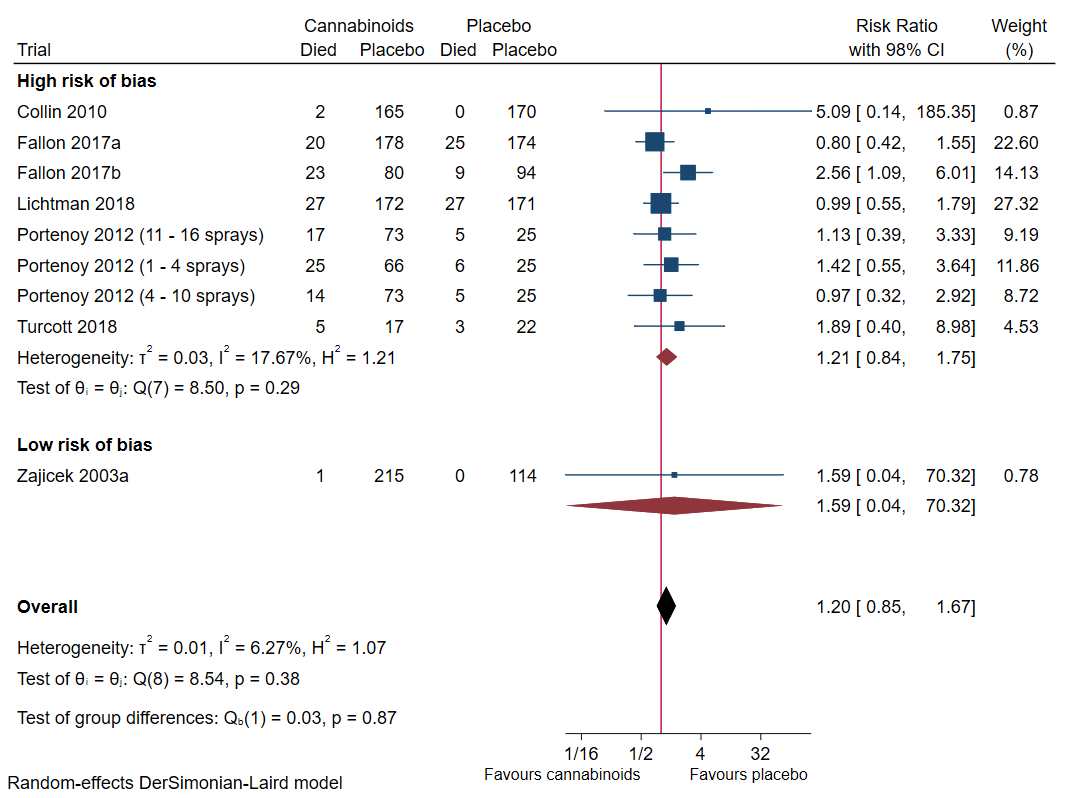


Figure 5: All-cause mortality subgroup analysis comparing trials assessed at high risk of bias to trials assessed at low risk of bias.


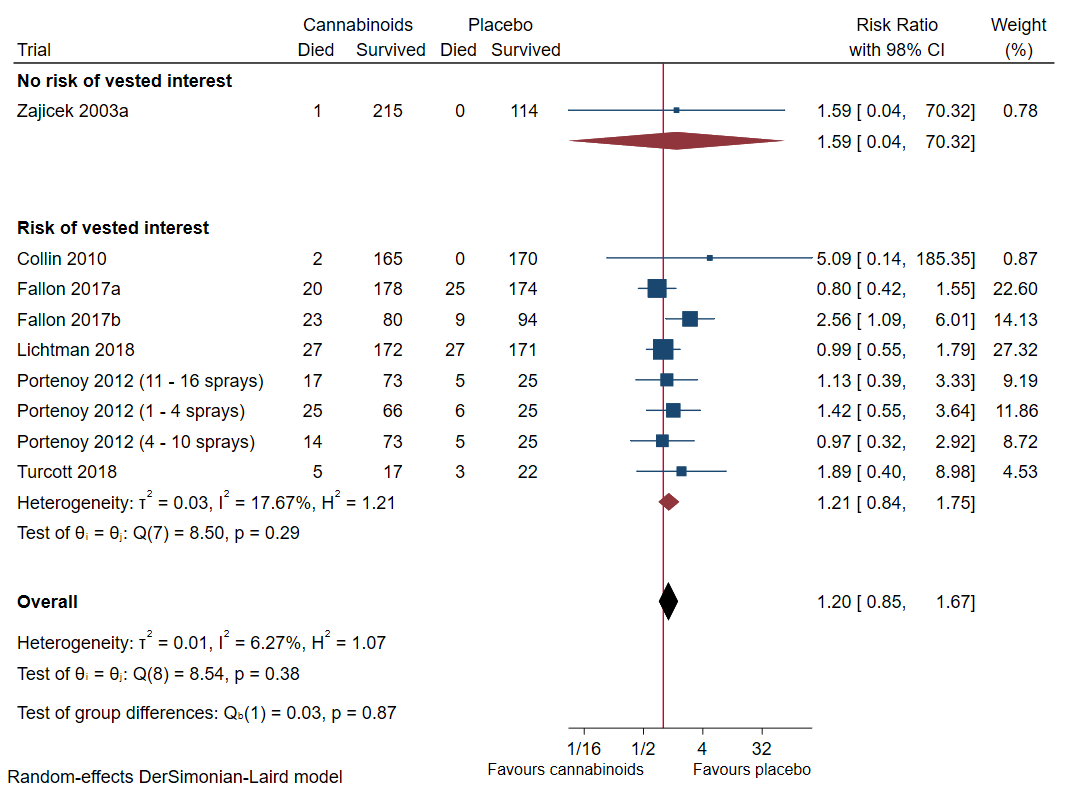


Figure 6: All-cause mortality subgroup analysis comparing trials at risk of vested interests and trials at no risk of vested interests.


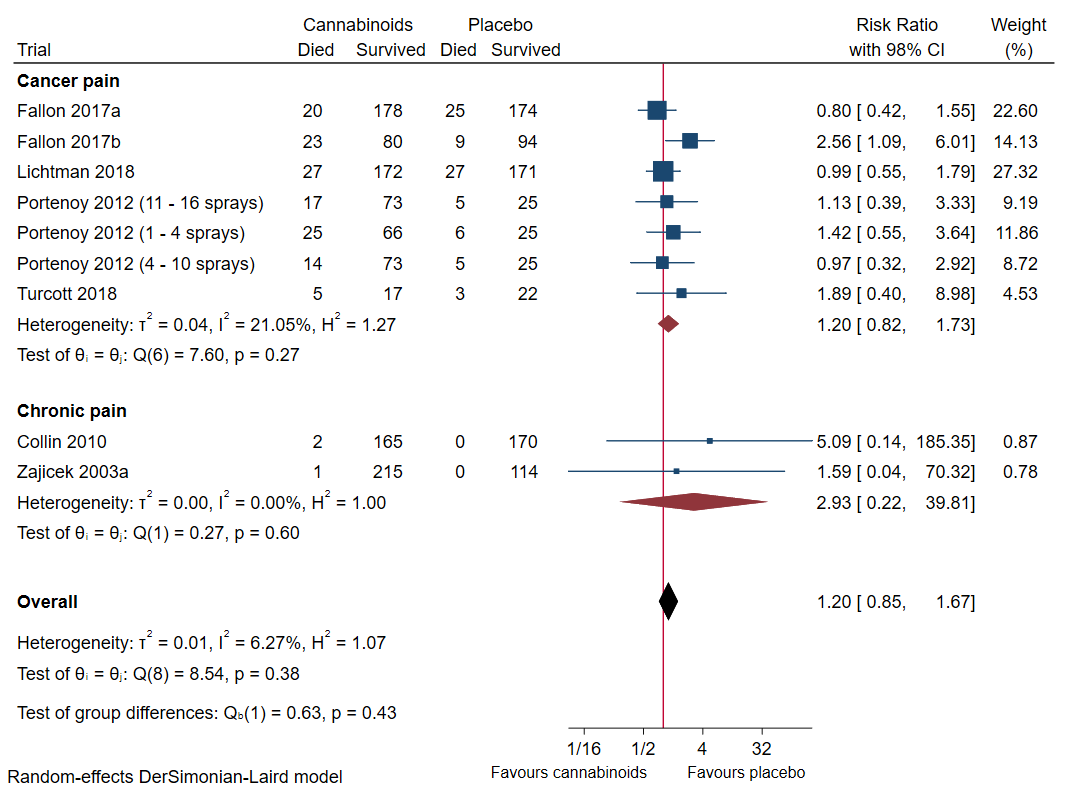


Figure 7: All-cause mortality subgroup analysis comparing trials randomising participants with cancer pain or chronic pain.


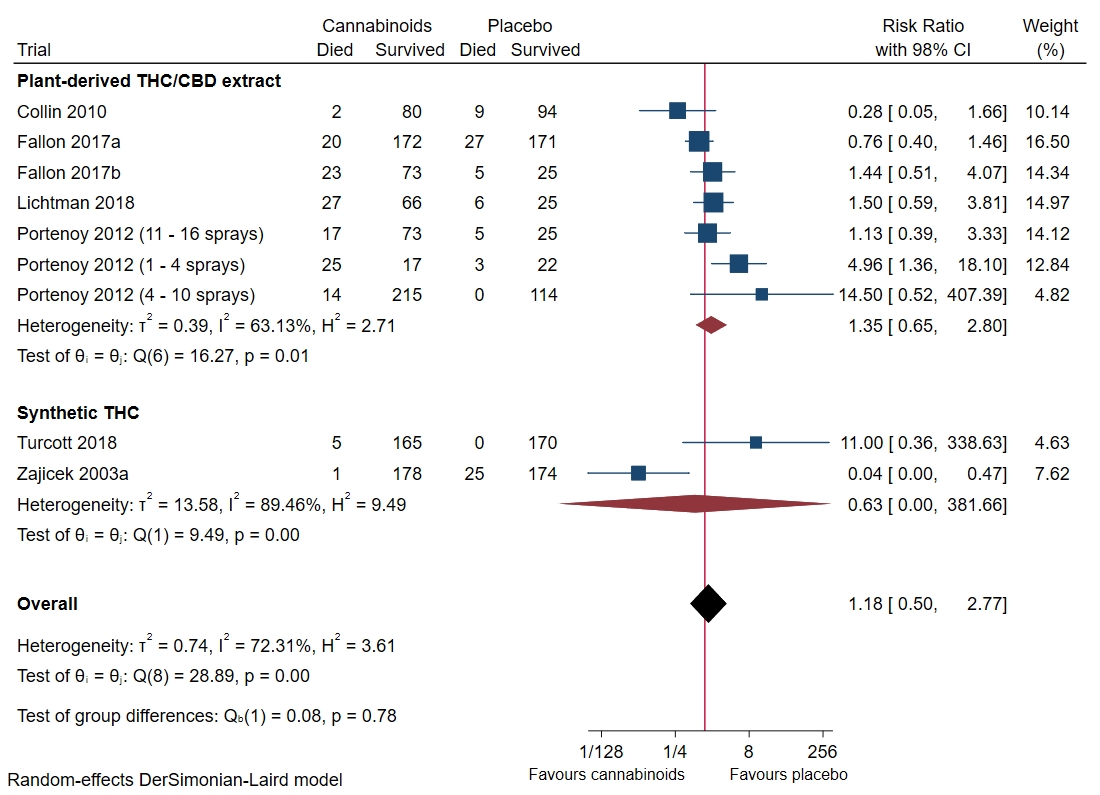


Figure 8: All-cause mortality subgroup analysis comparing plant-derived delta-9-tetrahydrocannabinol (THC)/ cannabidiol (CBD) with synthetic delta-9-tetrahydrocannabinol.

**6 Pain**


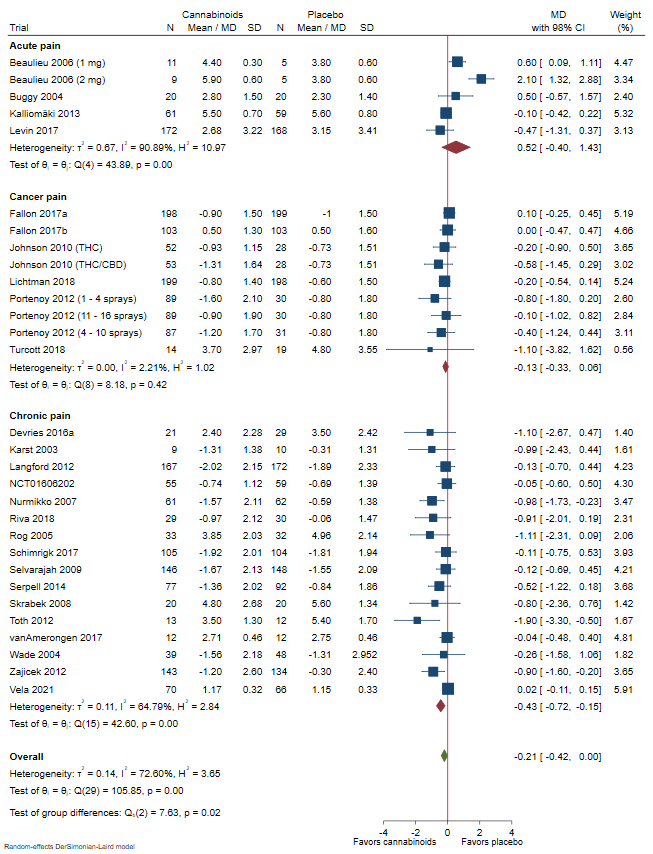


**Figure 9: Pain score subgroup analysis comparing trials randomising participants with acute pain, cancer pain or chronic pain. The subgroup analysis indicates a difference between the different types of pain (P=0.02)**

Acute pain


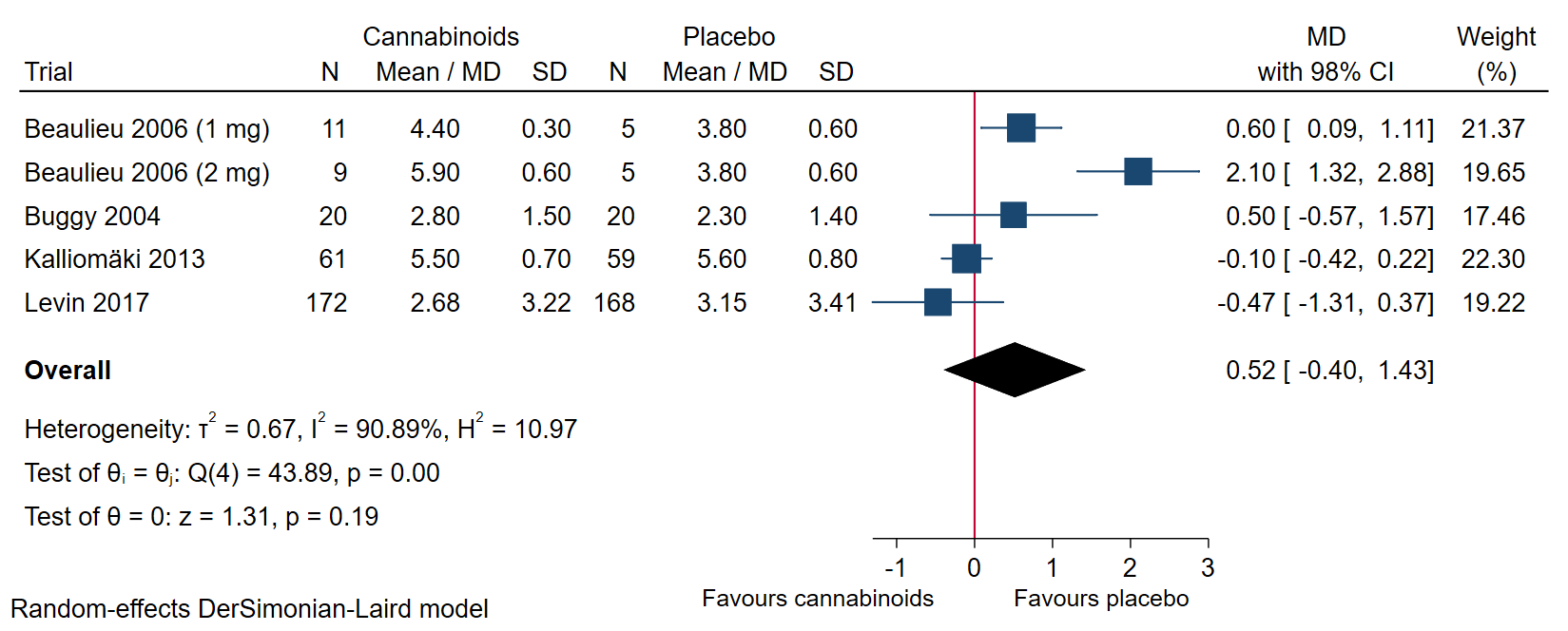


**Figure 10: Forest plot of the meta-analysis of acute pain with 98% CI.** The meta-analysis of acute pain showed no evidence of a statistically significant difference between cannabinoids and placebo. (SMD 0.42; 95% CI -0.14, 0.98; P=0.14)


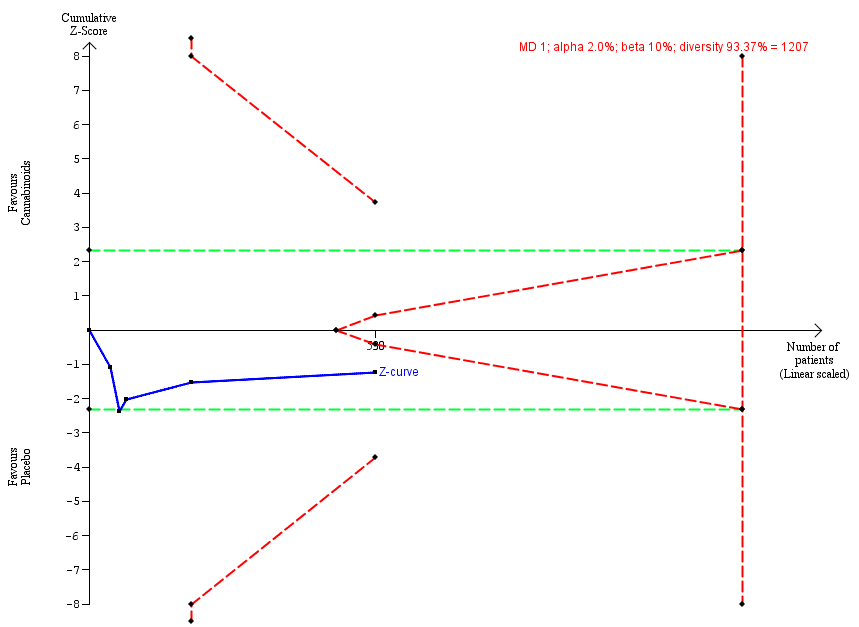


**Figure 11: Trial Sequential Analysis graph of acute pain using random-effects meta-analysis.** Trial Sequential Analysis showed the z-curve (the blue line) not breaching any boundary, showing that there was not enough information to confirm or reject that cannabinoids compared with placebo reduced acute pain (MD 0.52; 98% CI -1.03 to 2.06; P = 0.21).


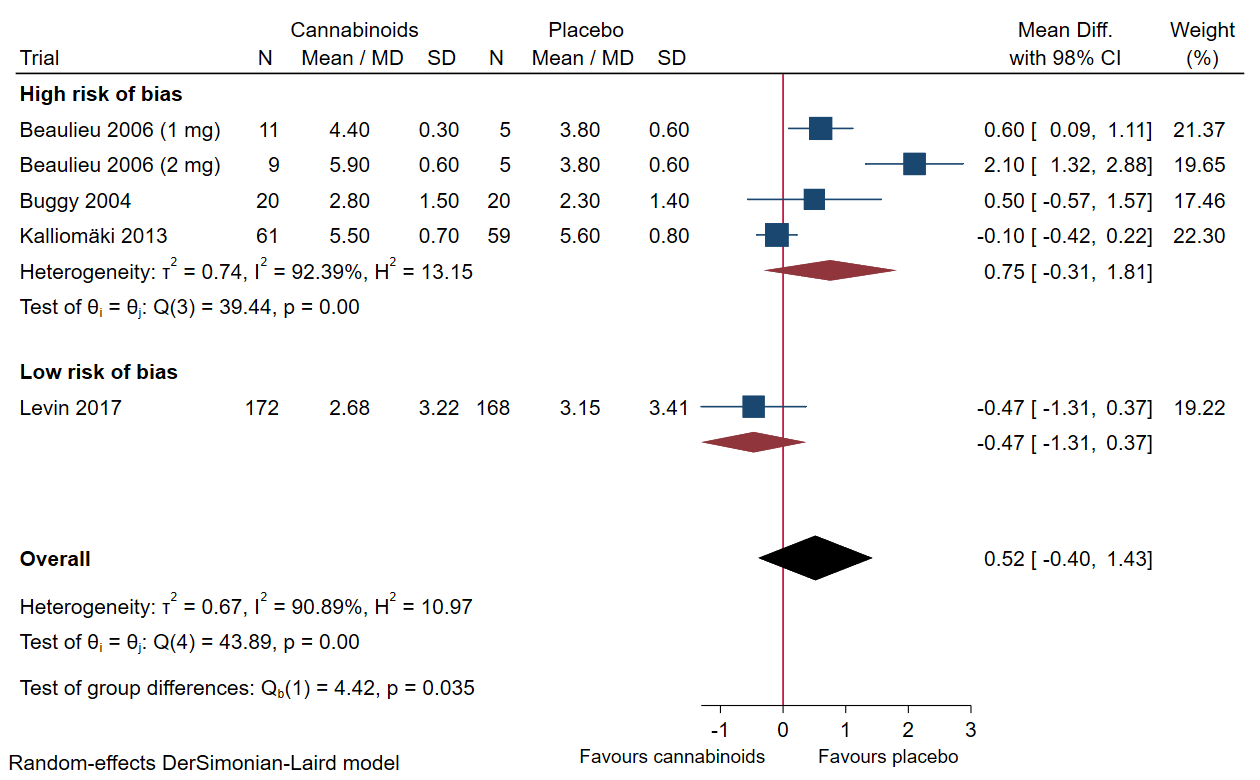


Figure 12: Acute pain subgroup analysis comparing trials assessed at high risk of bias to trials assessed at low risk of bias.


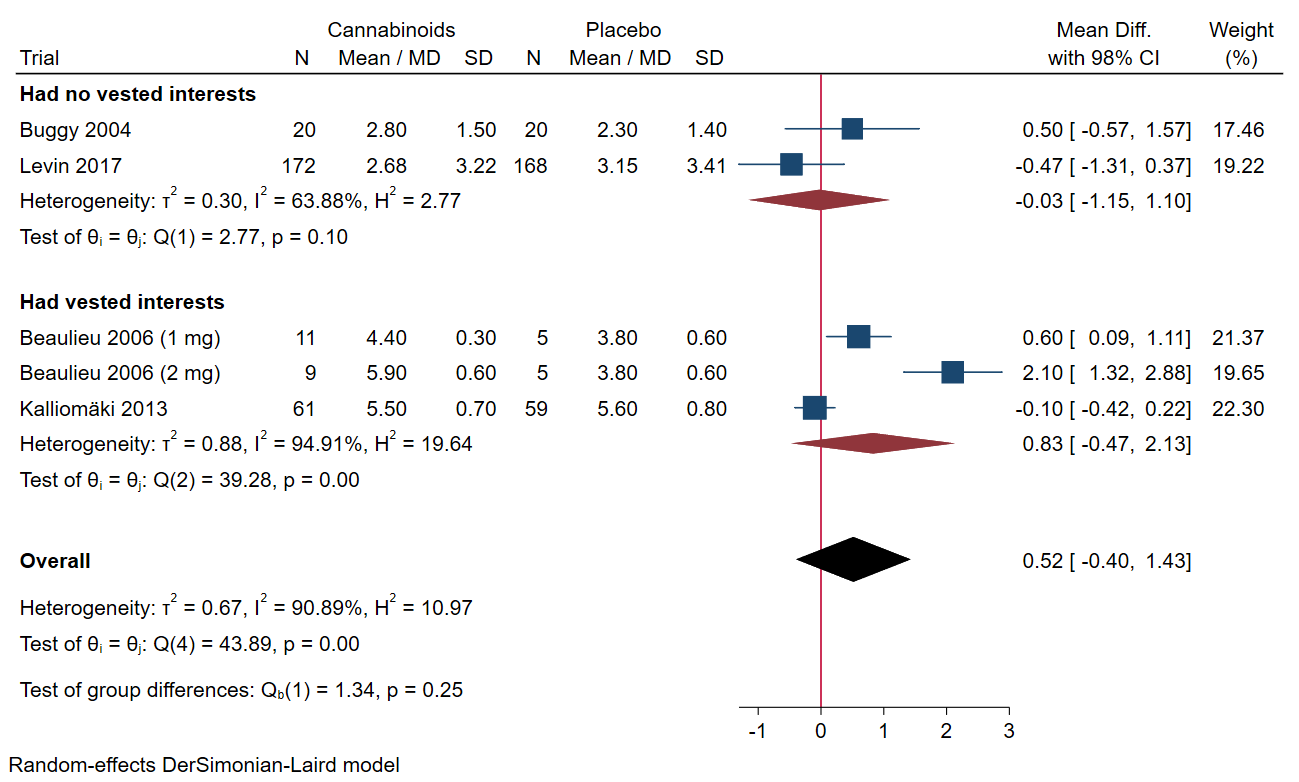


Figure 13: Acute pain subgroup analysis comparing trials at risk of vested interests and trials at no risk of vested interests.


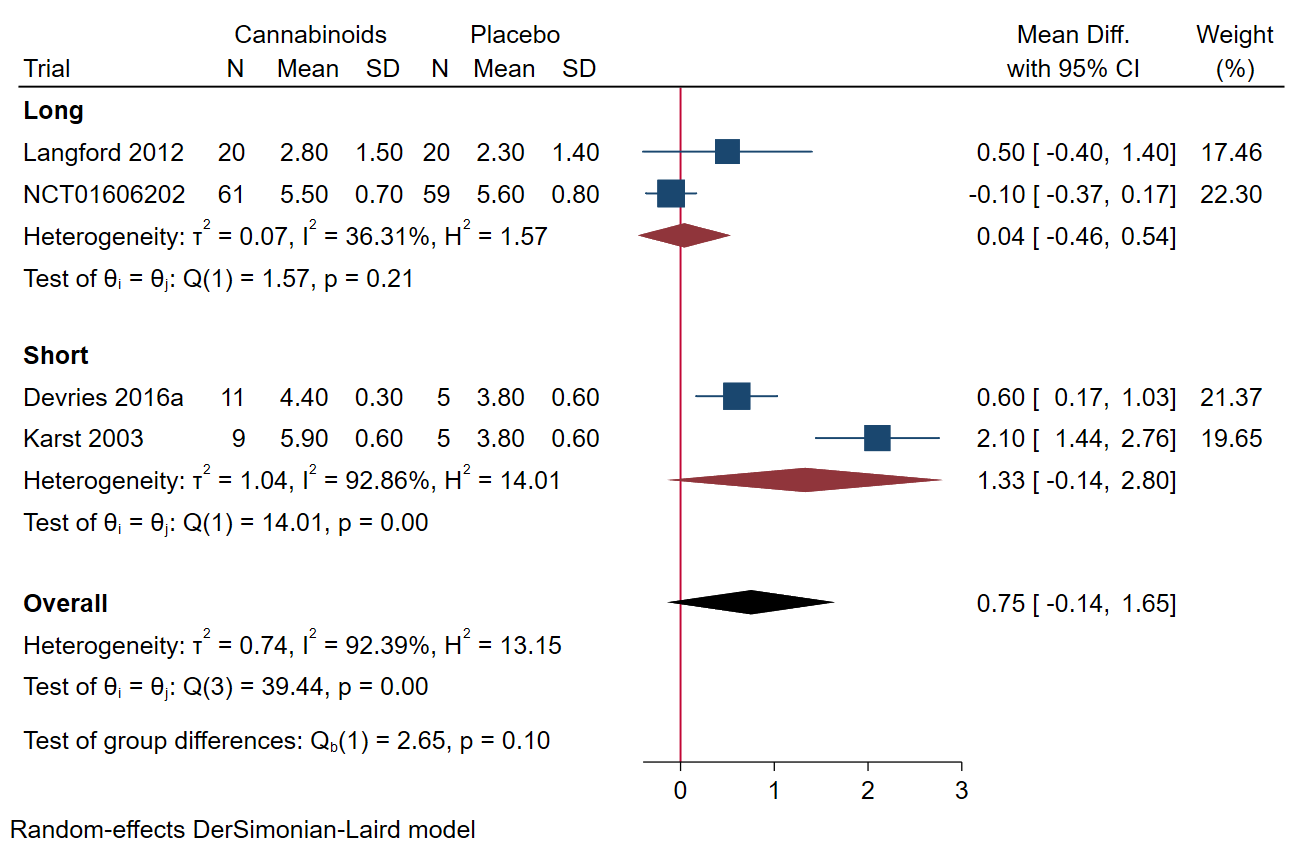


Figure 14: Forest plot of subgroup analysis comparing long-term follow-up with short-term follow-up in acute pain. Long-term defined as above the median and short-term defined below the median. The meta-analysis of acute pain showed no evidence of a statistically significant difference between long-term and short-term follow up in terms of acute pain (P=0.1).

Cancer pain


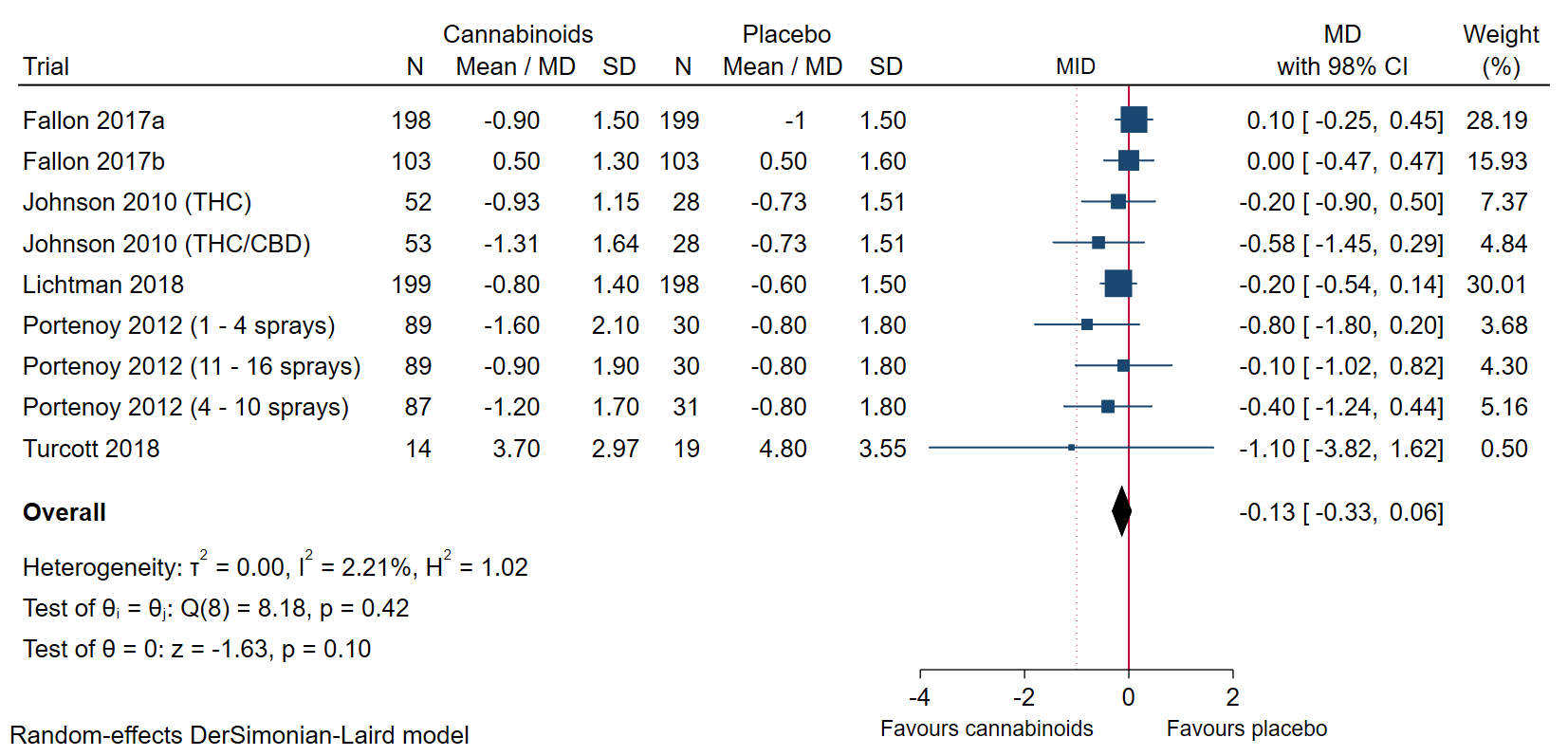


**Figure 15: Forest plot of the meta-analysis of cancer pain with 98% CI.** The meta-analysis of cancer pain showed no evidence of a statistically significant difference between cannabinoids and placebo. (SMD -0.09; 95% CI -0.20, 0.01; P= 0.07)

**
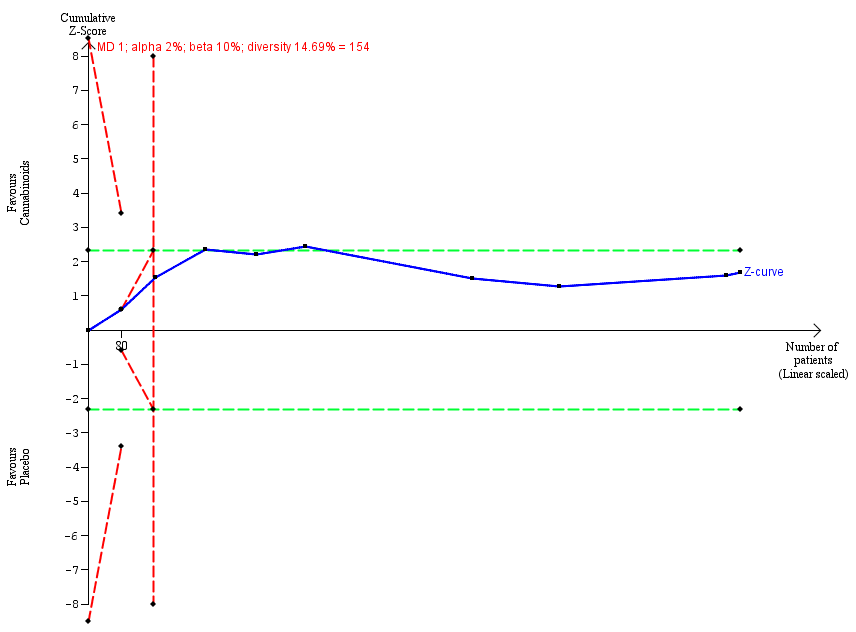
**

**Figure 16: Trial Sequential Analysis graph of cancer pain using random-effect meta-analysis.** Trial Sequential Analysis showed the z-curve (the blue line) breaching the boundary futility and showed that there was enough information to reject that cannabinoids compared with placebo reduced cancer pain.


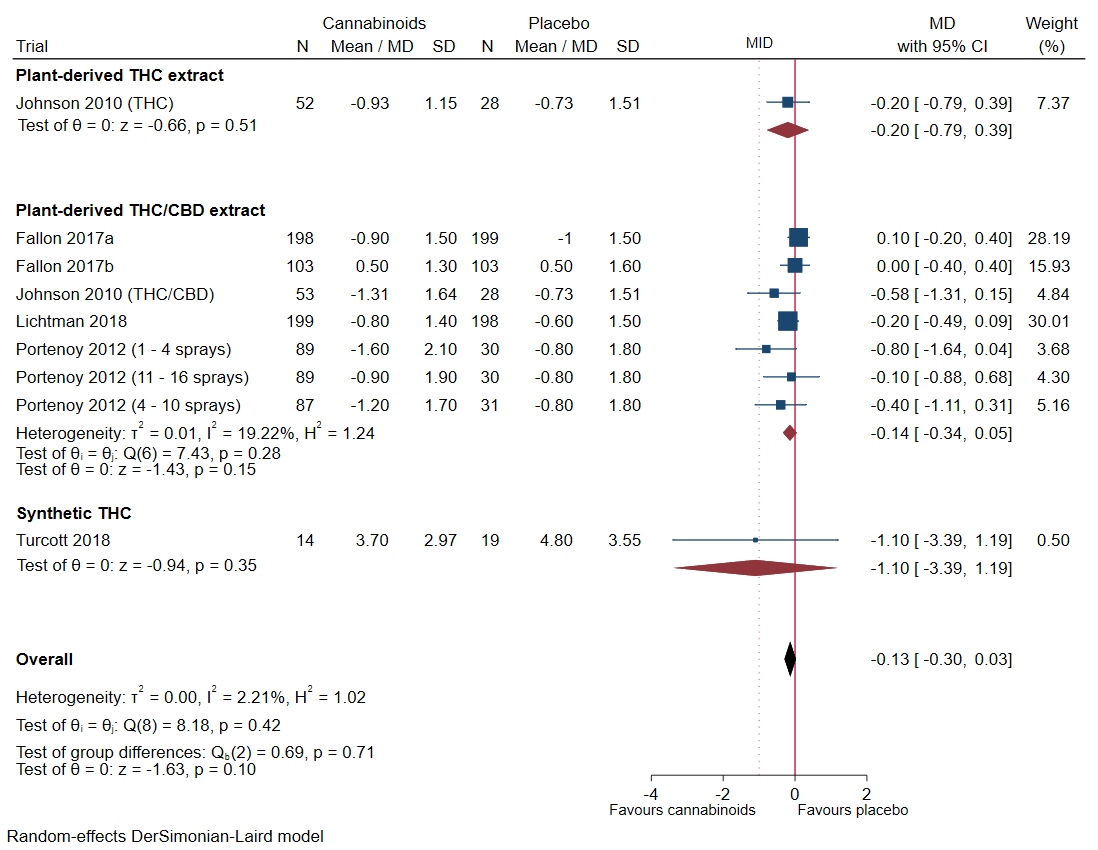


Figure 17: Cancer pain subgroup analysis comparing different types of cannabinoids.


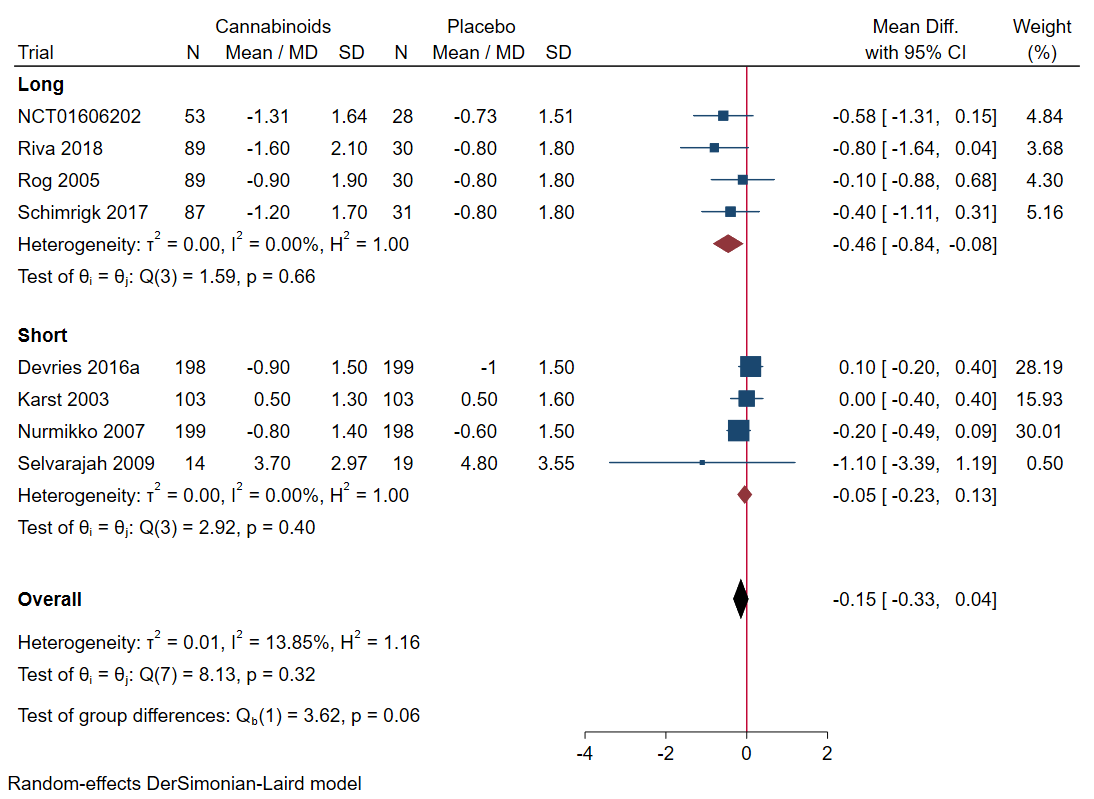


Figure 18: Forest plot of subgroup analysis comparing long-term follow-up with short-term follow-up in cancer pain. Long-term defined as above the median and short-term defined below the median. The meta-analysis of cancer pain showed no evidence of a statistically significant difference between long-term and short-term follow up in terms of cancer pain (P=0.06).

Chronic pain


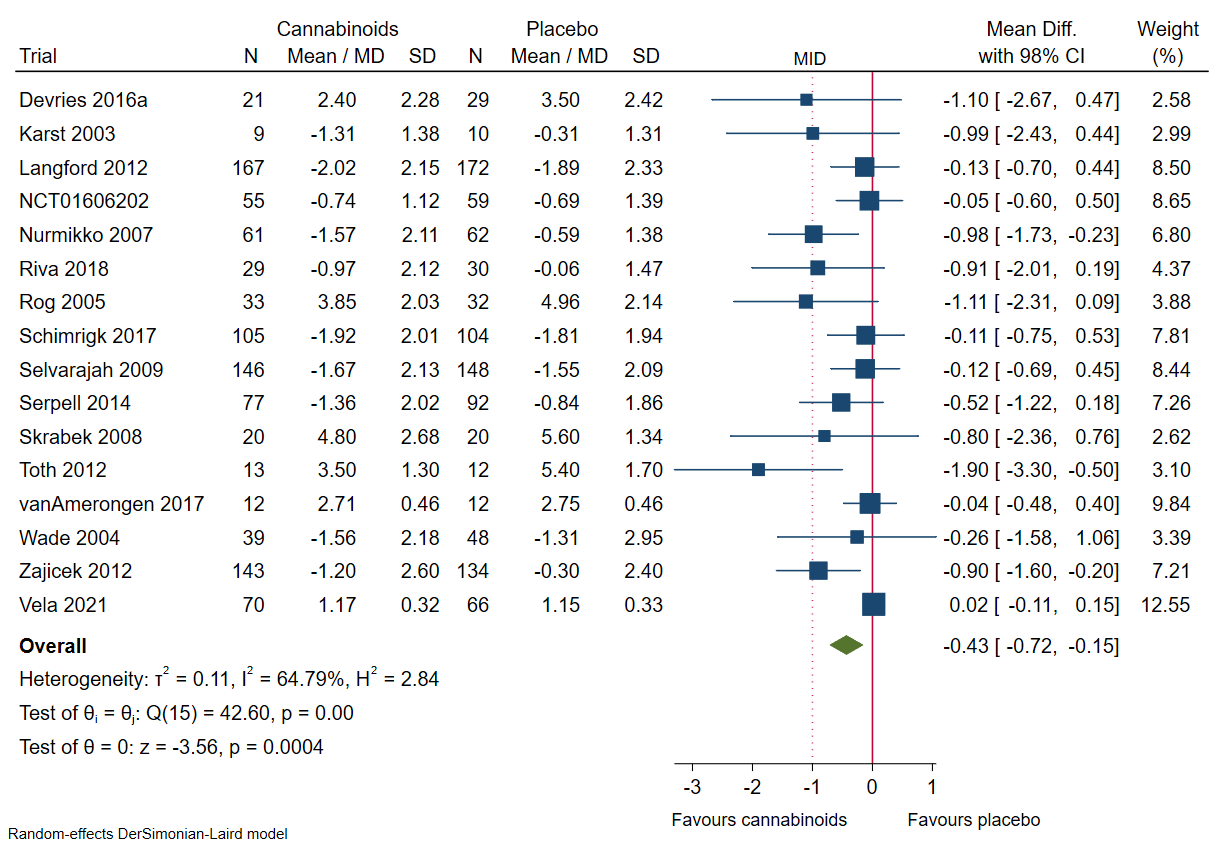


**Figure 19: Forest plot of the meta-analysis of chronic pain with 98% CI.** The meta-analysis of chronic pain showed evidence of a statistically significant difference between cannabinoids and placebo. (SMD -0.25; 95% CI -0.37, -0.13; P < 0.0001).


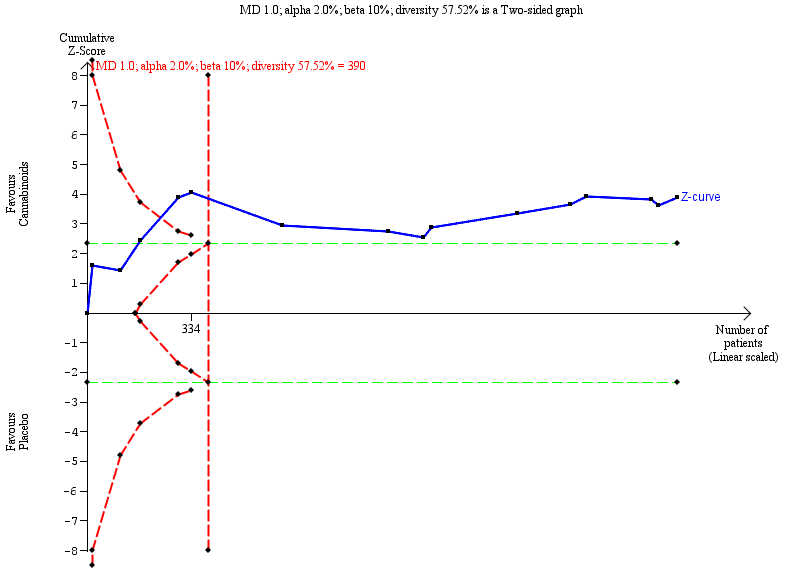


**Figure 20: Trial Sequential Analysis graph of chronic pain score using random-effects meta-analysis.** Trial Sequential Analysis showed the z-curve (the blue line) breaching the boundary of benefit, showing that there was enough information to confirm that cannabinoids compared with placebo reduced chronic pain.


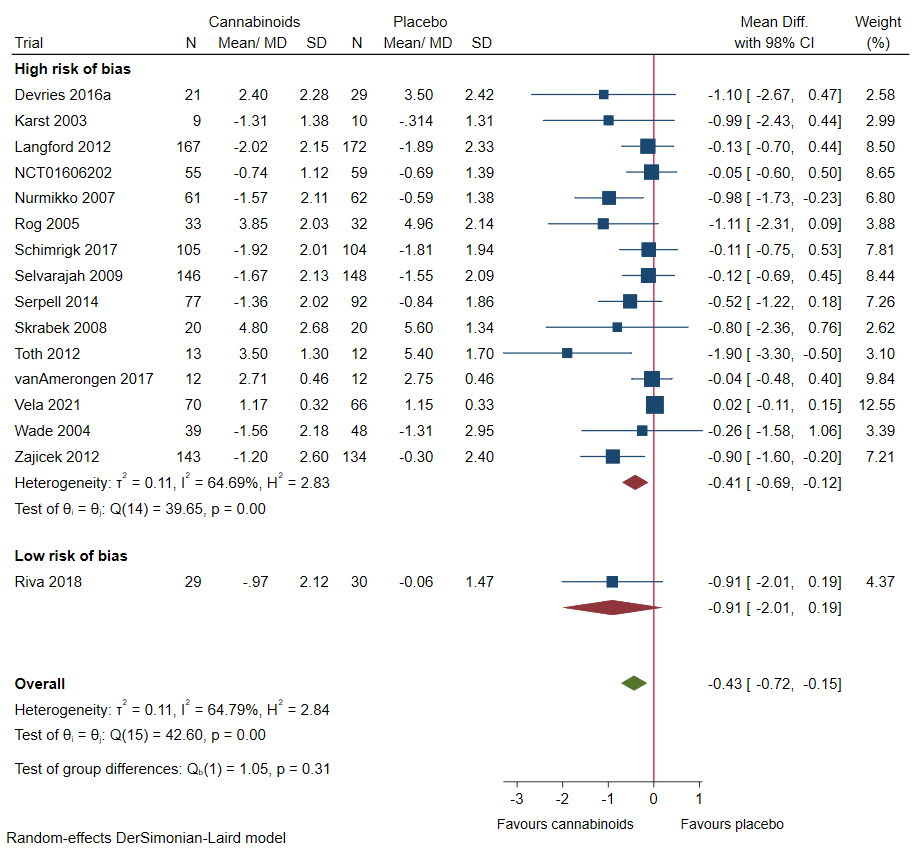


Figure 21: Chronic pain subgroup analysis comparing trials assessed at high risk of bias to trials assessed at low risk of bias.


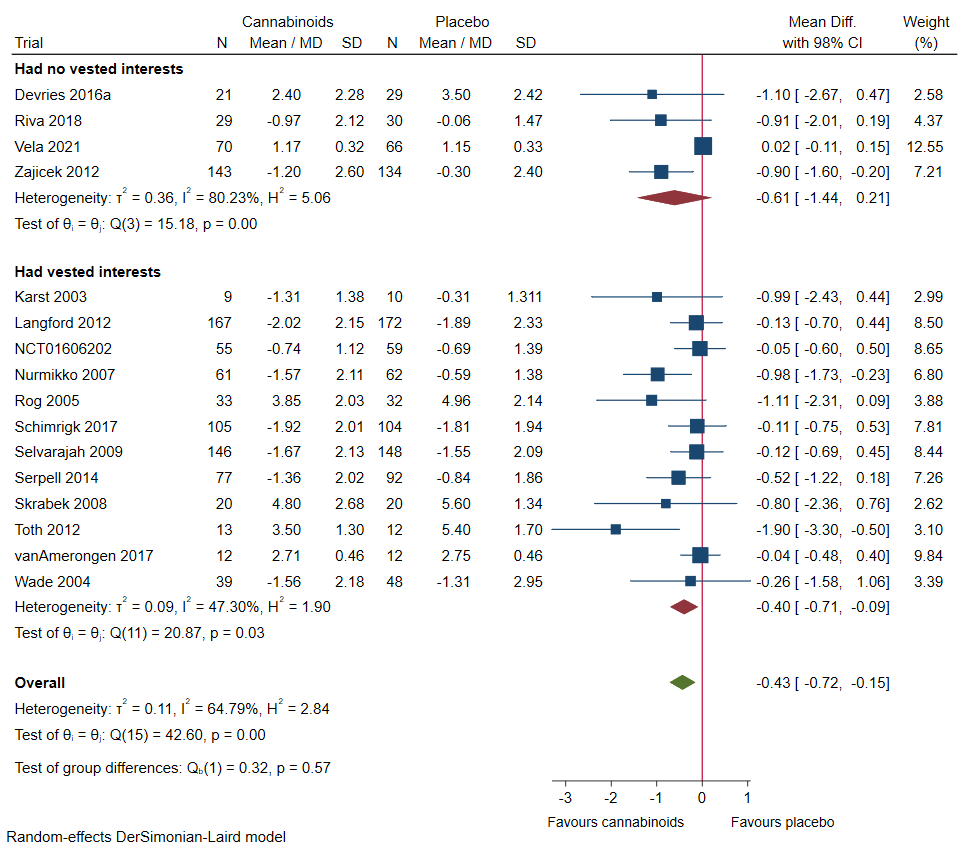


Figure 22: Chronic pain subgroup analysis comparing trials at risk of vested interests and trials at no risk of vested interests.


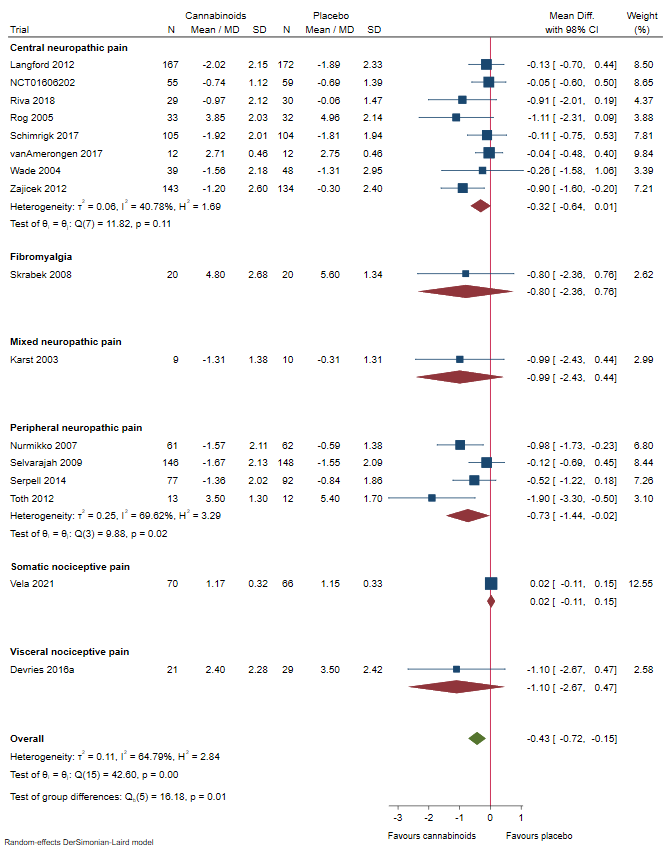


Figure 23: Chronic pain subgroup analysis comparing trials randomising participants with neuropathic pain (central, peripheral and mixed), fibromyalgia and visceral nociceptive pain.


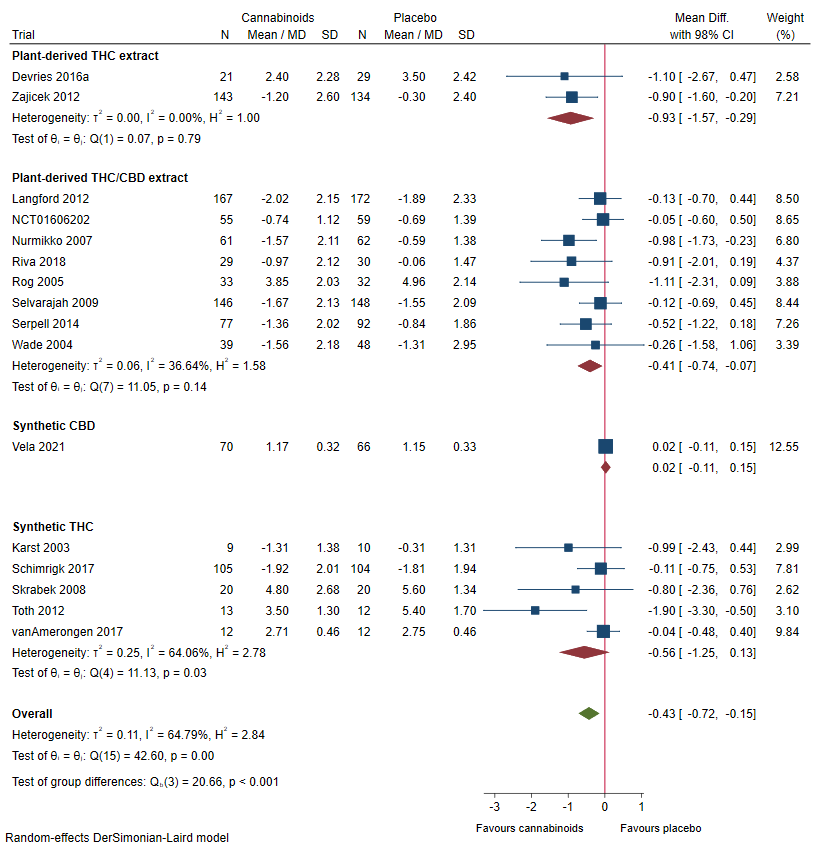


Figure 24: Chronic pain subgroup analysis comparing different types of cannabinoids.


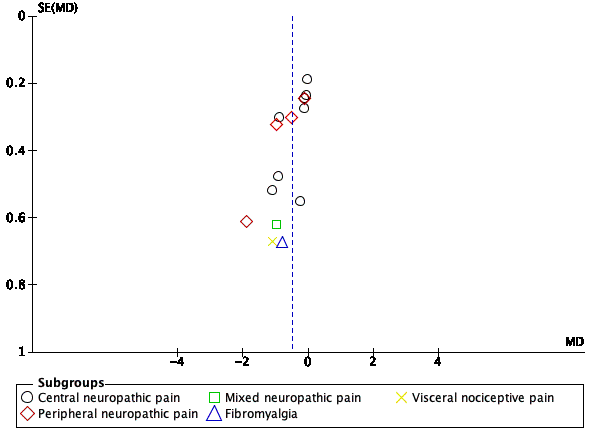


**Figure 25: Funnel plot of chronic pain.** The funnel plot showed no signs of small-study effects.

Regression-based Egger test for small-study effects: Random-effects model: Method: DerSimonian-Laird

H0: beta1 = 0; no small-study effects

beta1 = -2.61

SE of beta1 = 0.675

z = -3.87

Prob > |z| = 0.0001


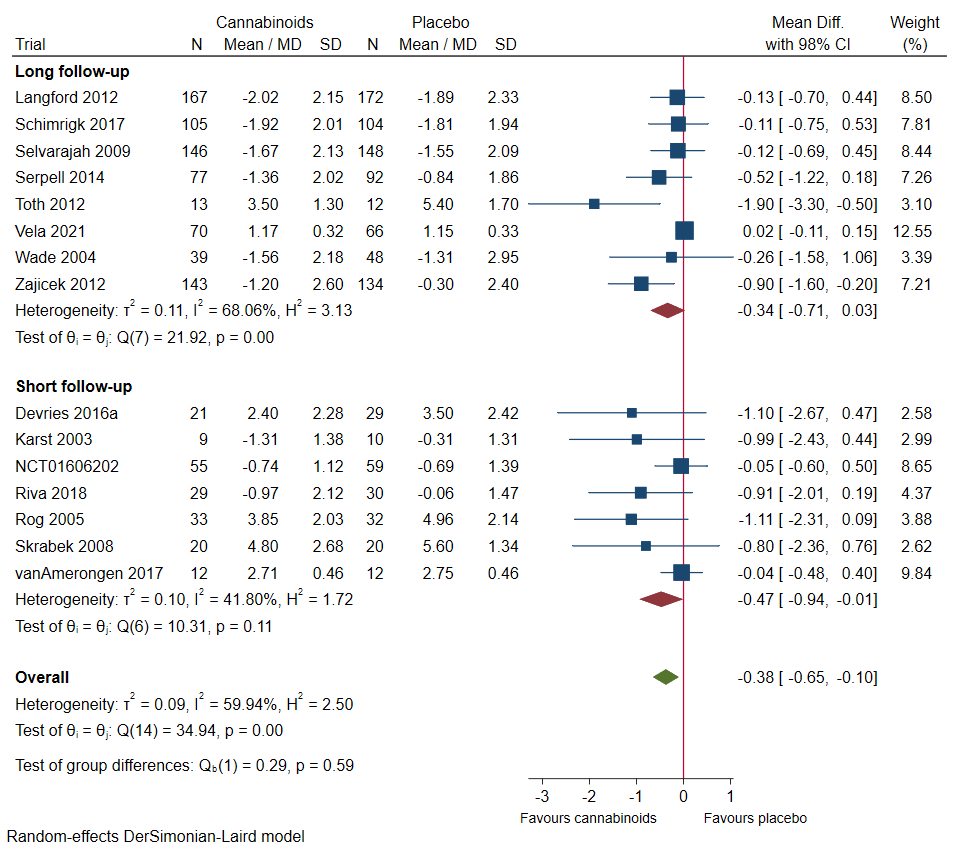


Figure 26: Forest plot of subgroup analysis comparing long-term follow-up with short-term follow-up in chronic pain**.** Long-term defined as above the median and short-term defined below the median. The meta-analysis of chronic pain showed no evidence of a statistically significant difference between long-term and short-term follow up in terms of chronic pain (P=0.59).

**7 Serious adverse events**


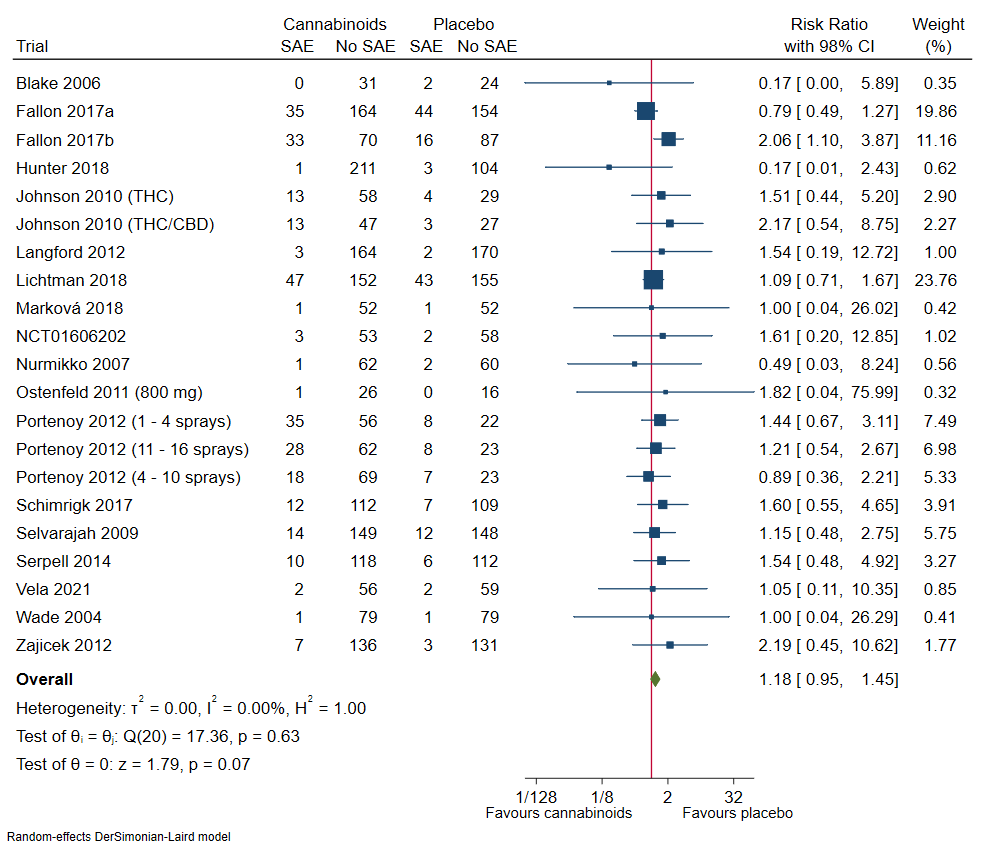


**Figure 27: Forest plot of the meta-analysis of serious adverse events with 98% CI.** The meta-analysis of serious adverse events showed no statistically significant evidence of a difference between cannabinoids and placebo.


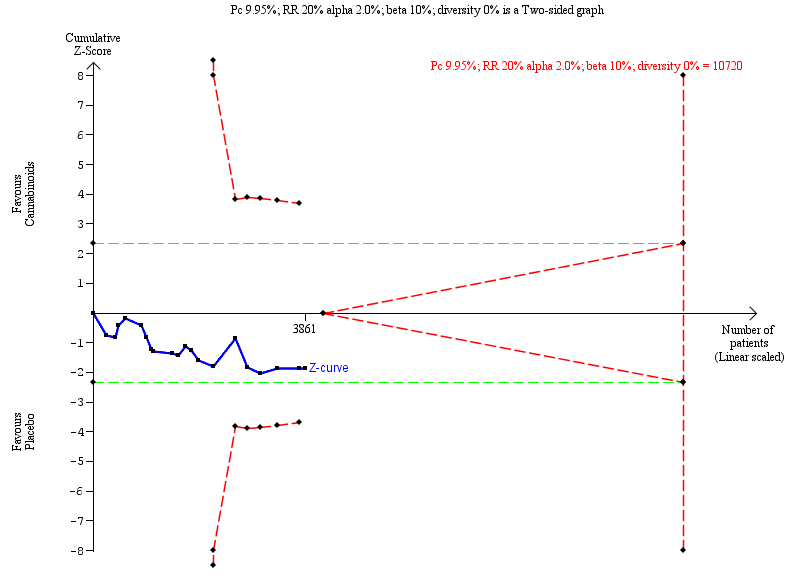


**Figure 28: Trial Sequential Analysis graph of serious adverse event using random-effects meta-analysis.** Trial Sequential Analysis showed the z-curve (the blue line) not breaching the boundary of harm, showing that there was not enough information to confirm or reject that cannabinoids compared with placebo increased the risk of serious adverse events by 20% (RR 1.18; 98% CI 0.95 to 1.46; P = 0.07).


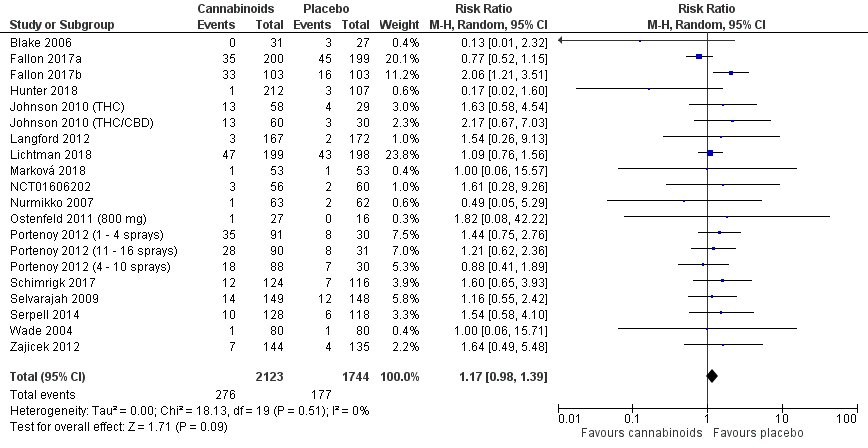


Figure 29: Serious adverse events best-worst case scenario


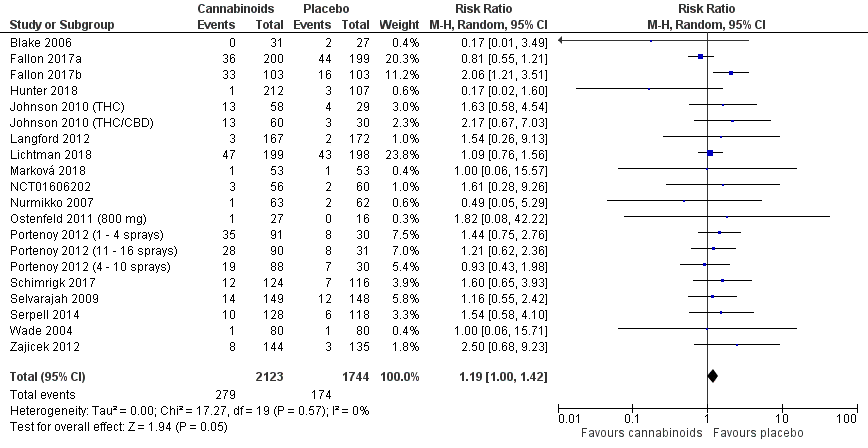


Figure 30: Serious adverse events worst-best case scenario


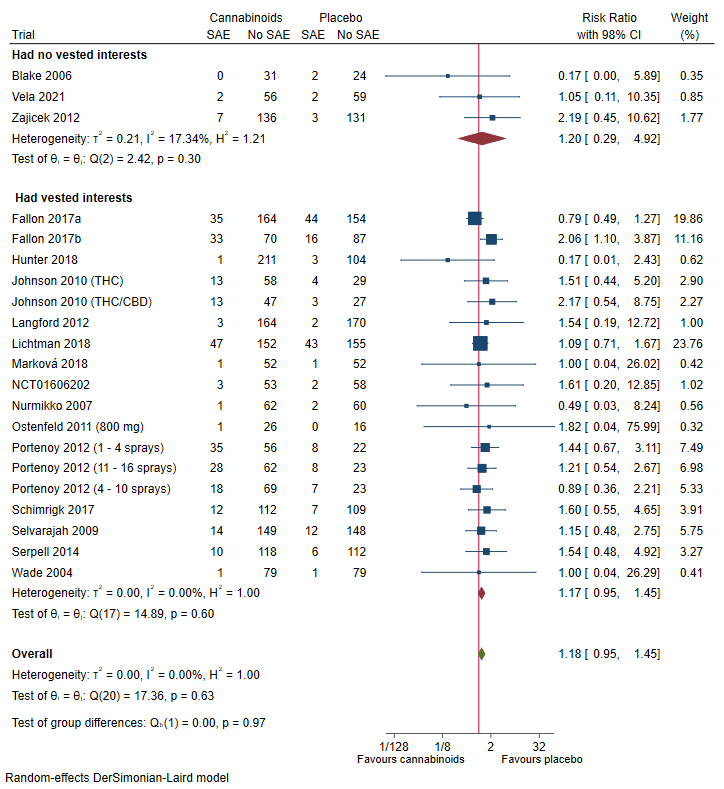


Figure 31: Serious adverse events subgroup analysis comparing trials at risk of vested interests and trials at no risk of vested interests.


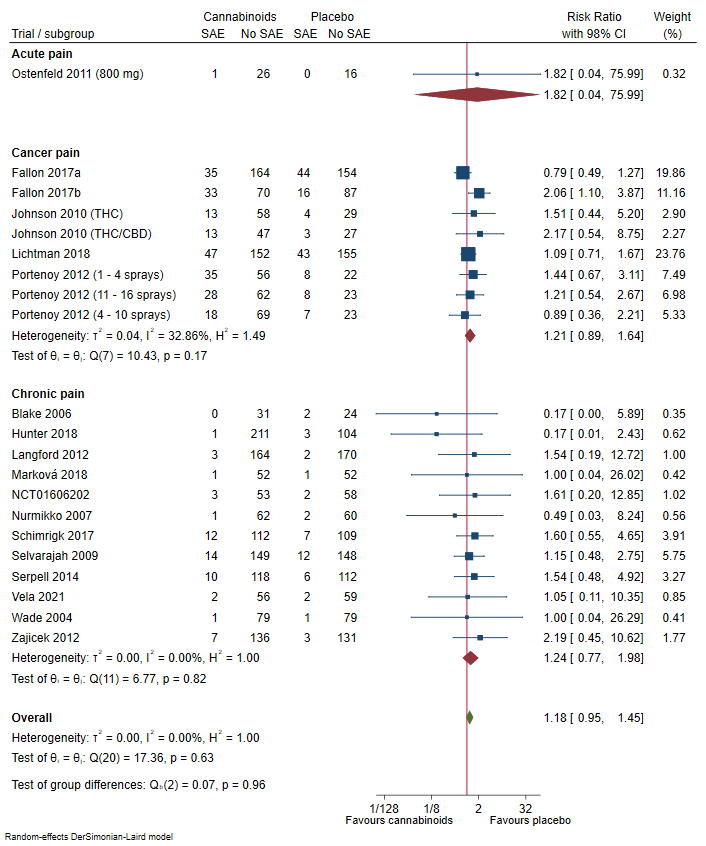


Figure 32: Serious adverse events subgroup analysis comparing trials randomising participants with acute pain, cancer pain or chronic pain.


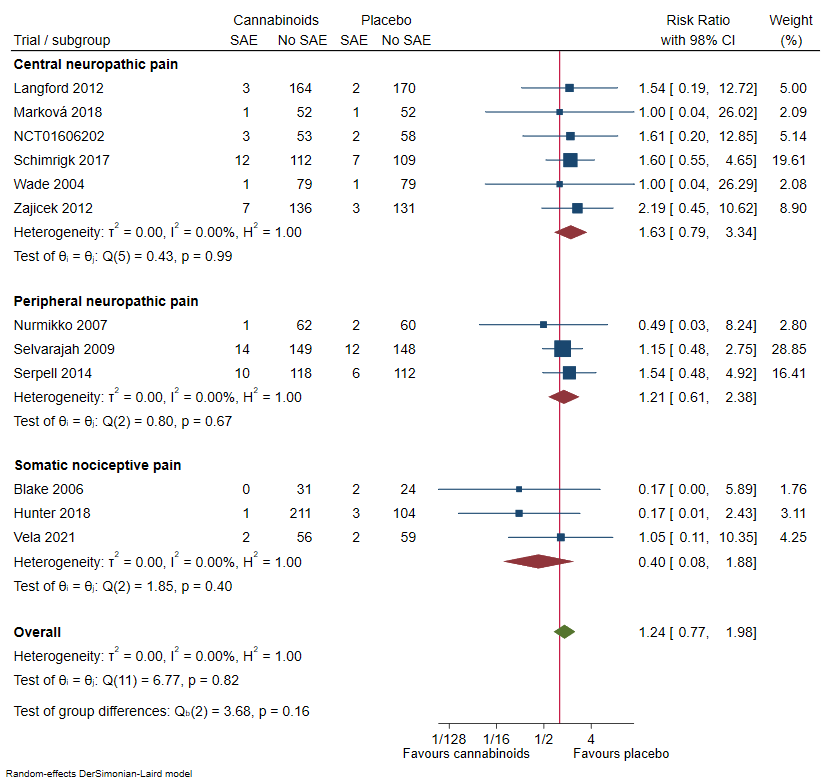


Figure 33: Serious adverse events subgroup analysis comparing trials randomising participants with neuropathic pain (central and peripheral) and somatic nociceptive pain.


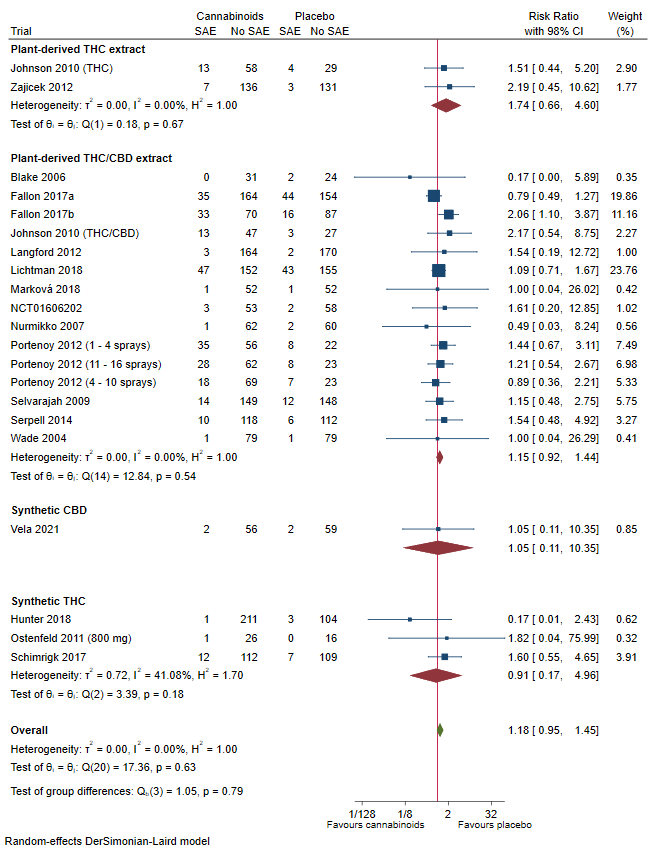


Figure 34: Serious adverse events subgroup analysis comparing different types of cannabinoids**.**


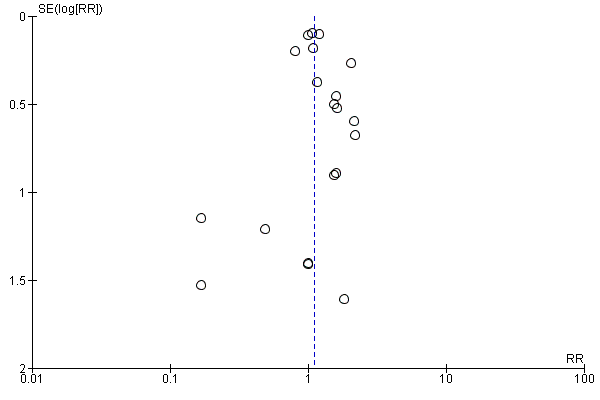
**Figure 35: Funnel plot of serious adverse events.** The funnel plot showed no signs of small-study effects.

Regression-based Harbord test for small-study effects: Random-effects model: Method: DerSimonian-Laird

H0: beta1 = 0; no small-study effects

beta1 = -0.15

SE of beta1 = 0.427

z = -0.36

Prob > |z| = 0.7171

**8 Quality of life**


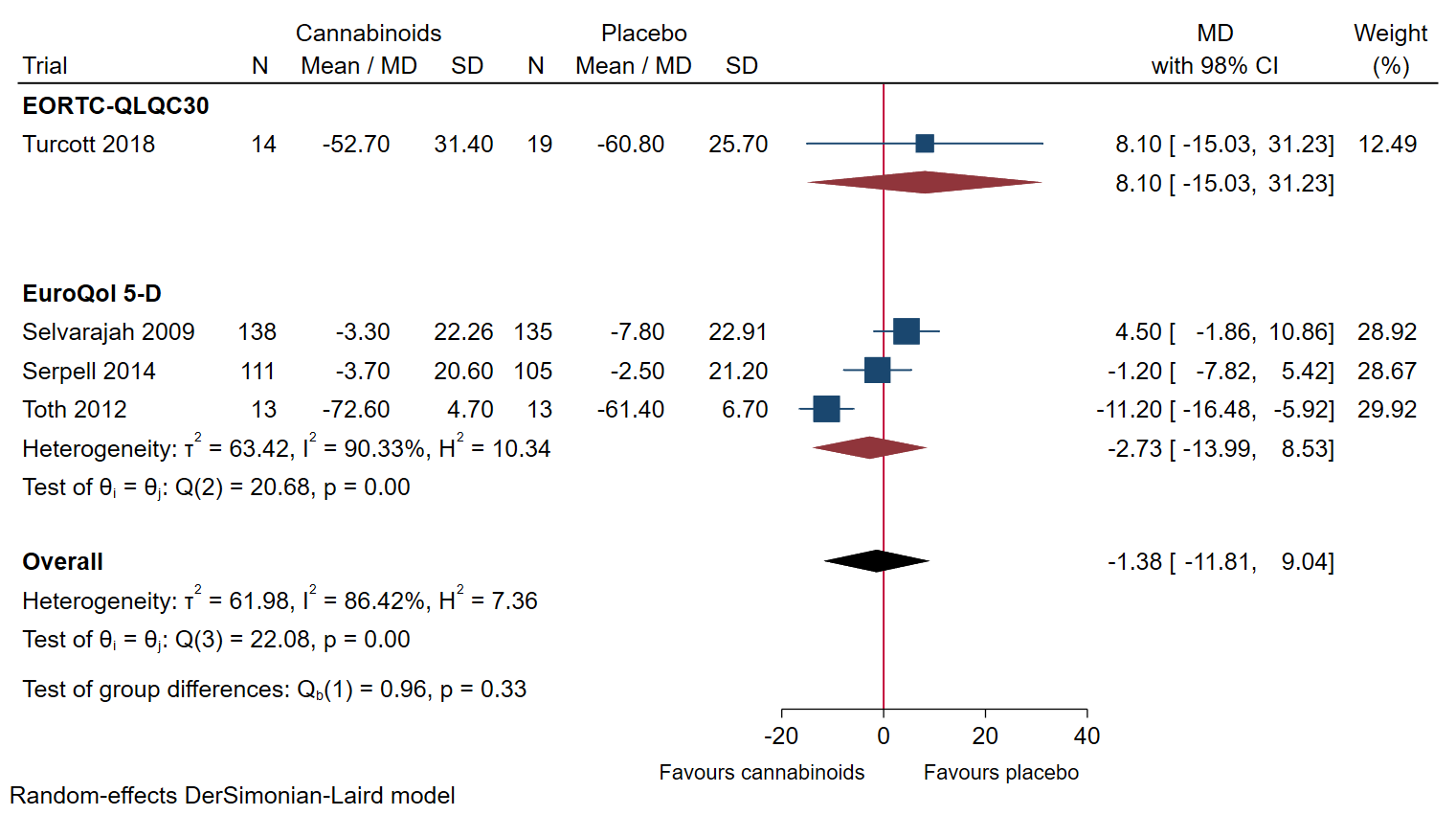


**Figure 36: Forest plot of the meta-analysis of quality of life with 98% CI.** The meta-analysis of quality of life showed no statistically significant evidence of a difference between cannabinoids and placebo. (SMD -0.19; 95% CI -0.70, 0.32; P= 0.46)


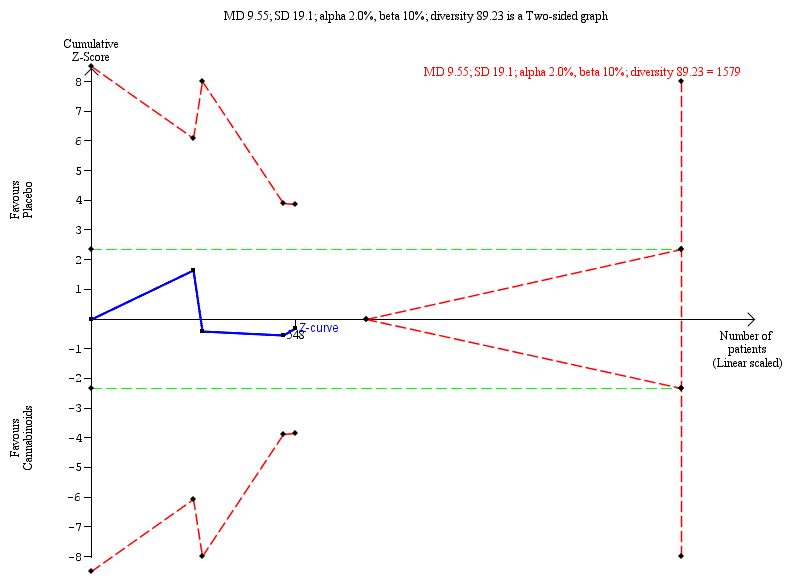


**Figure 37: Trial Sequential Analysis graph of quality of life using random-effects meta-analysis.** Trial Sequential Analysis showed the z-curve (the blue line) not breaching any boundary, showing that there was not enough information to confirm or reject that cannabinoids compared with placebo improved quality of life (MD -1.43; 98% CI -11.81 to 9.04; P = 0.75).


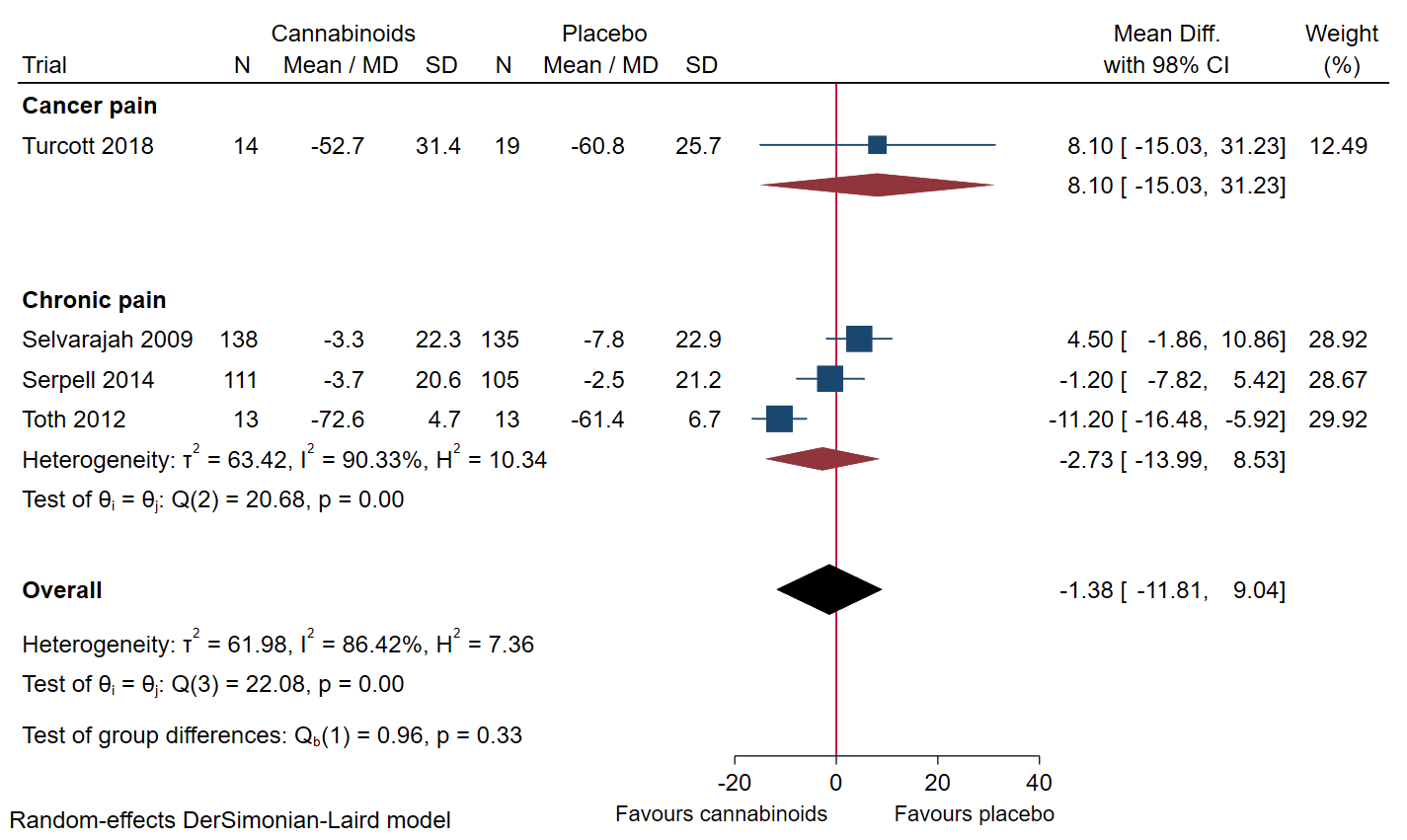


Figure 38: Quality of life subgroup analysis comparing trials randomising participants with cancer pain and chronic pain.


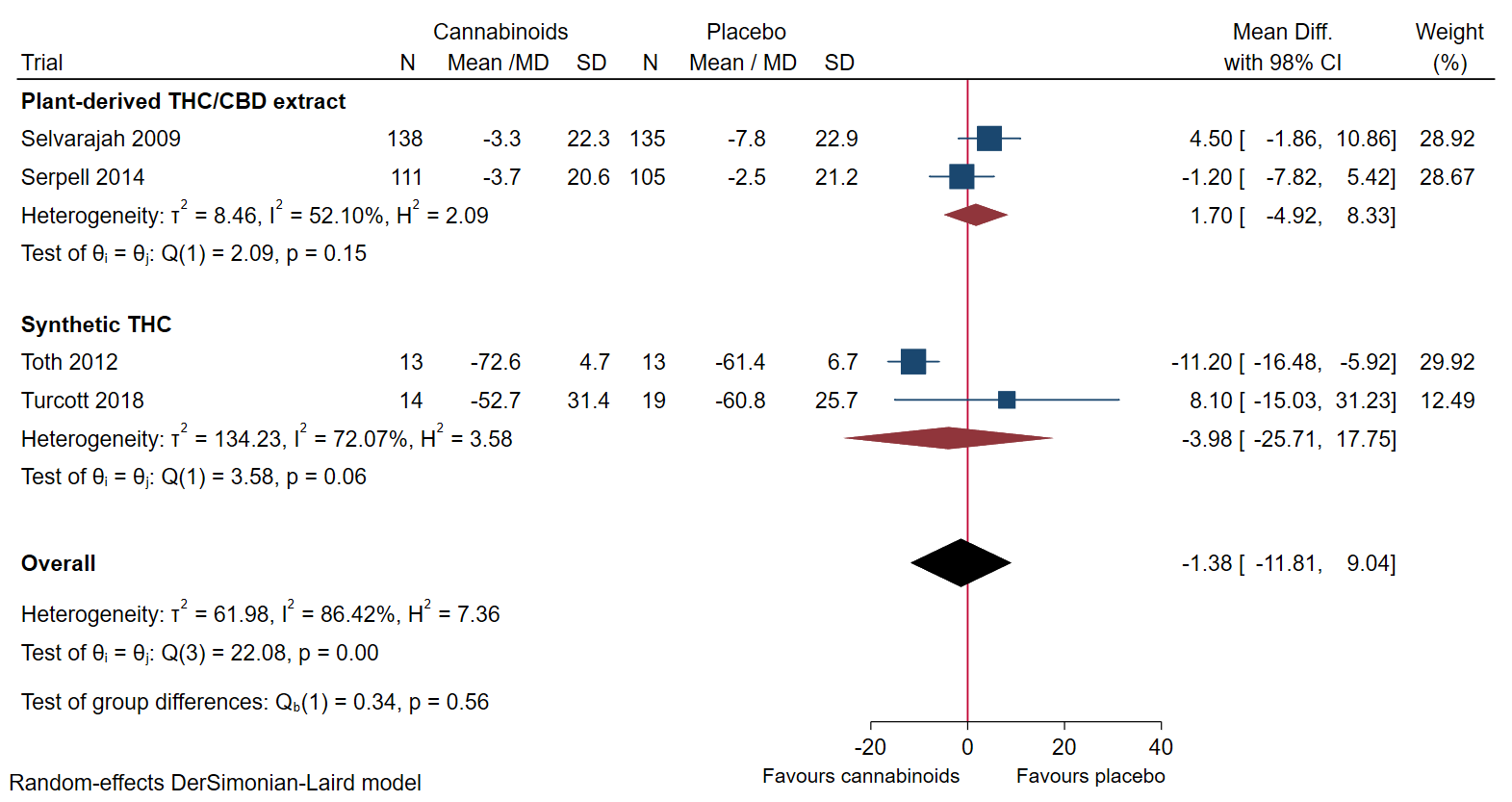


Figure 39: Quality of life subgroup analysis comparing different types of cannabinoids.

**9 Non-serious adverse events**


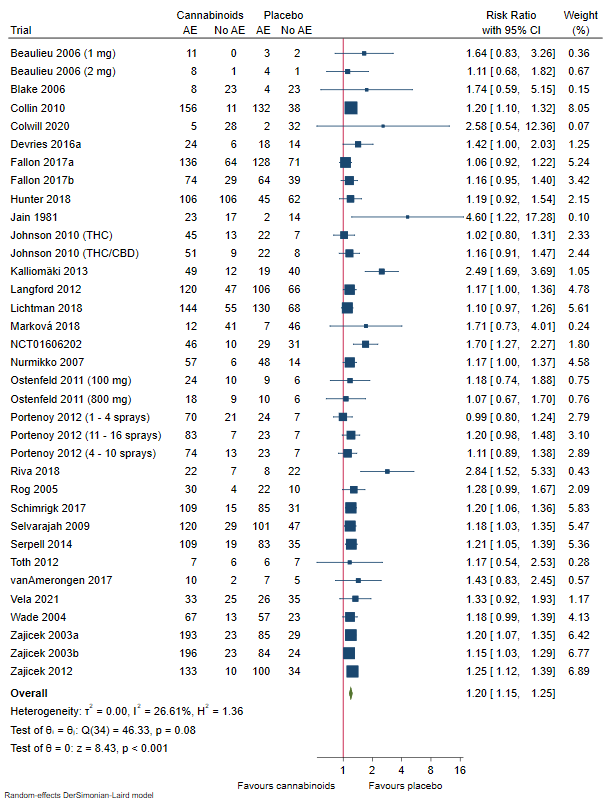


**Figure 40: Forest plot of the meta-analysis of non-serious adverse events with 95% CI.** The meta-analysis of non-serious adverse events showed evidence of a statistically significant difference between cannabinoids and placebo.


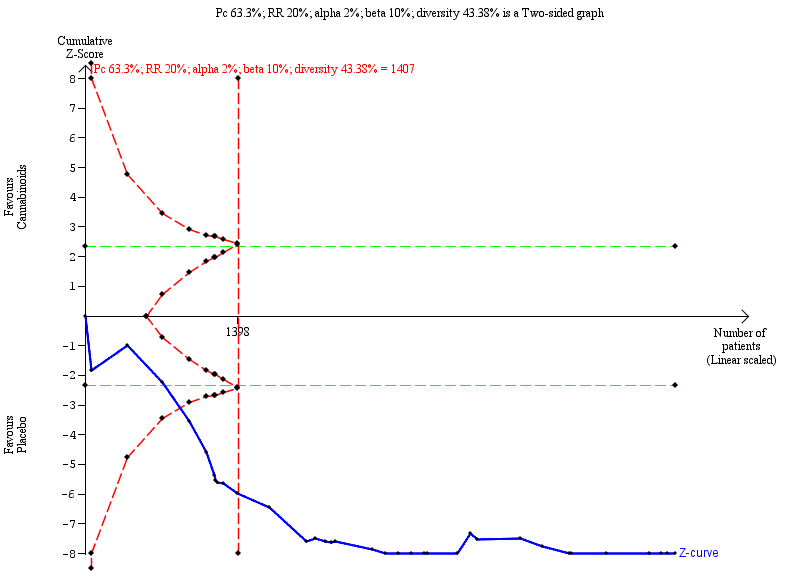


**Figure 41: Trial Sequential Analysis graph of non-serious adverse event using random-effects meta-analysis.** Trial Sequential Analysis showed the z-curve (the blue line) breaching the boundary of harm, showing that there was enough information to confirm that cannabinoids compared with placebo increased the risk of non-serious adverse events by 20%.


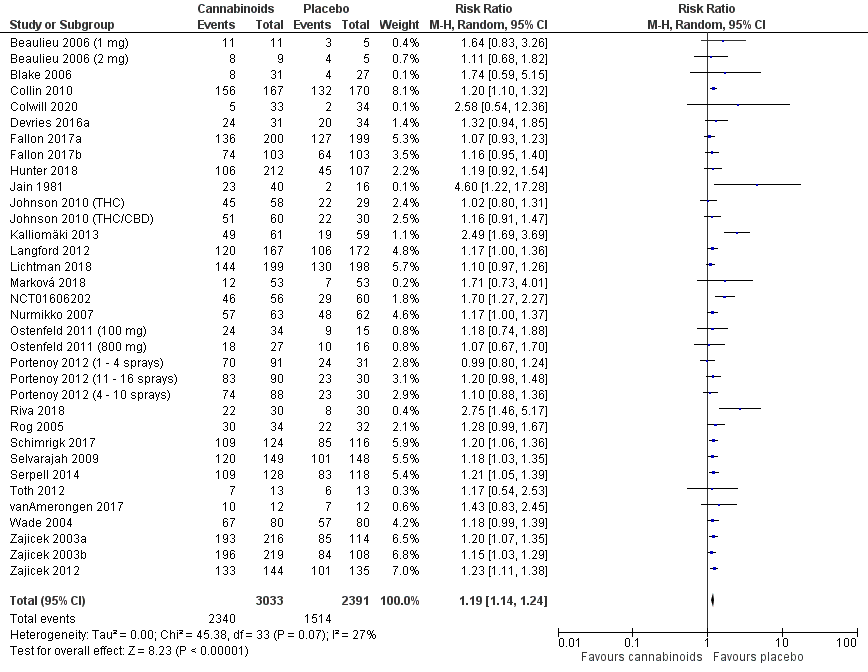


Figure 42: Non-serious adverse events best-worst case scenario


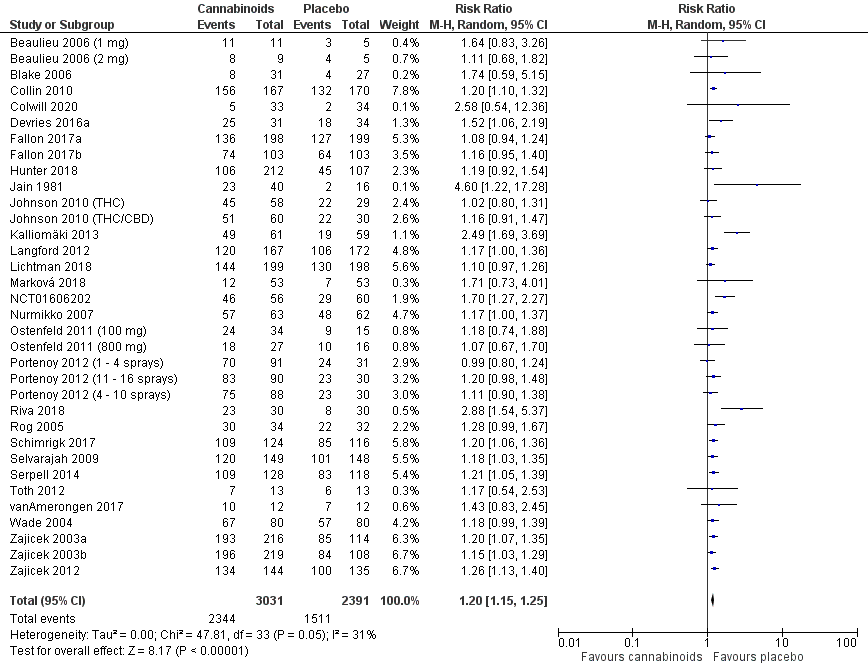


Figure 43: Non-serious adverse events worst-best case scenario


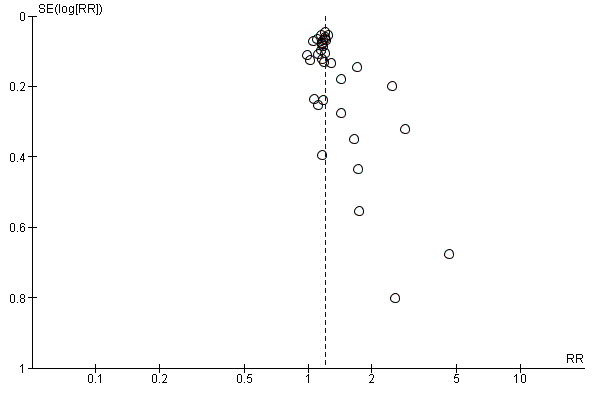


**Figure 44: Funnel plot of non-serious adverse events.** The funnel plot showed signs of small-study effects.

Regression-based Harbord test for small-study effects: Random-effects model: Method: DerSimonian-Laird

H0: beta1 = 0; no small-study effects

beta1 = 1.22

SE of beta1 = 0.366

z = 3.34

Prob > |z| = 0.0008

**10 Quality of sleep**


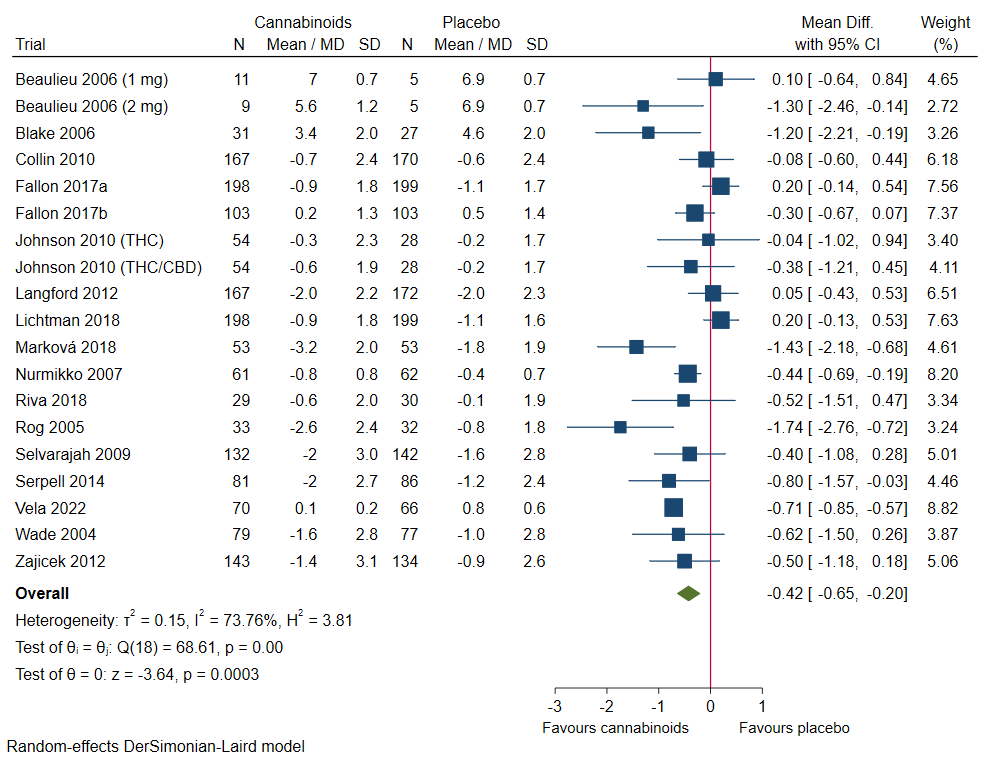


**Figure 45: Forest plot of the meta-analysis of quality of sleep with 95% CI.** The meta-analysis of quality of sleep showed evidence of a statistically significant difference between cannabinoids and placebo. (SMD -0.21; 95% CI -0.34, -0.08; P=0.001).


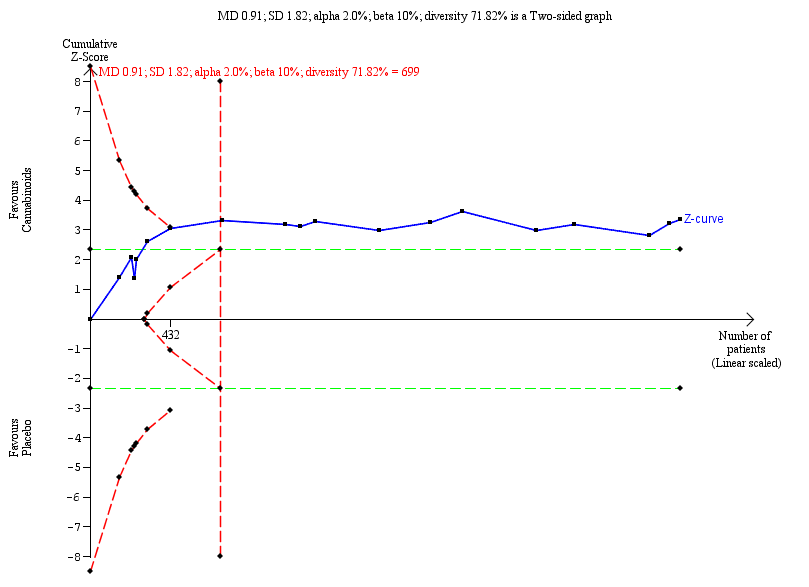


**Figure 46: Trial Sequential Analysis graph of quality of sleep using random-effects meta-analysis.** Trial Sequential Analysis showed the z-curve (the blue line) breaching the boundary of benefit, showing that there was enough information to confirm that cannabinoids compared with placebo improved quality of sleep.


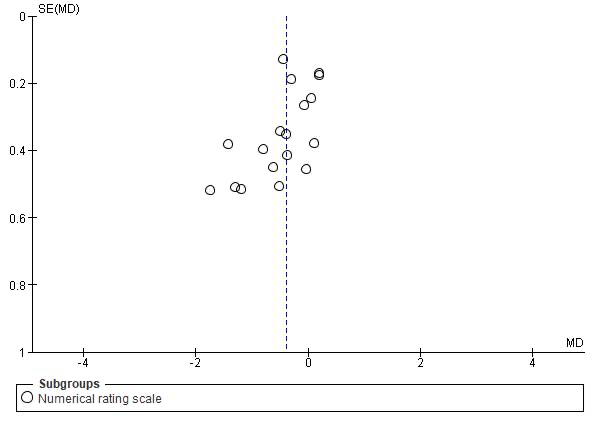


**Figure 47: Funnel plot of quality of sleep.** The funnel plot showed no signs of small-study effects.

Regression-based Egger test for small-study effects: Random-effects model: Method: DerSimonian-Laird

H0: beta1 = 0; no small-study effects

beta1 = -2.35

SE of beta1 = 0.798

z = -2.95

Prob > |z| = 0.0032

**11 Twenty-four hours morphine consumption**


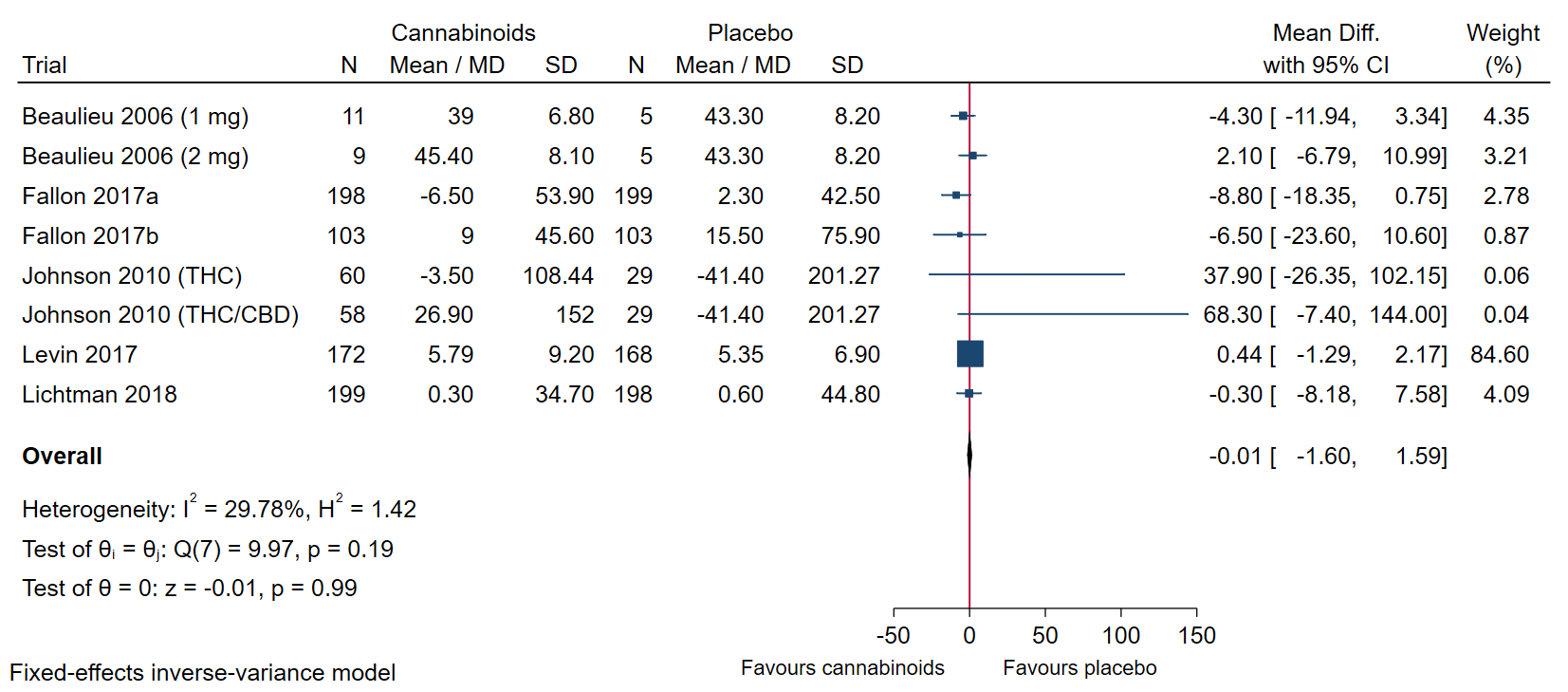


**Figure 48: Forest plot of the meta-analysis of 24 hour morphine consumption with 95% CI.** The meta-analysis of 24 hour morphine consumption showed no statistically significant evidence of a difference between cannabinoids and placebo. (SMD -0.02; 95% CI -0.12, 0.08; P=0.69).


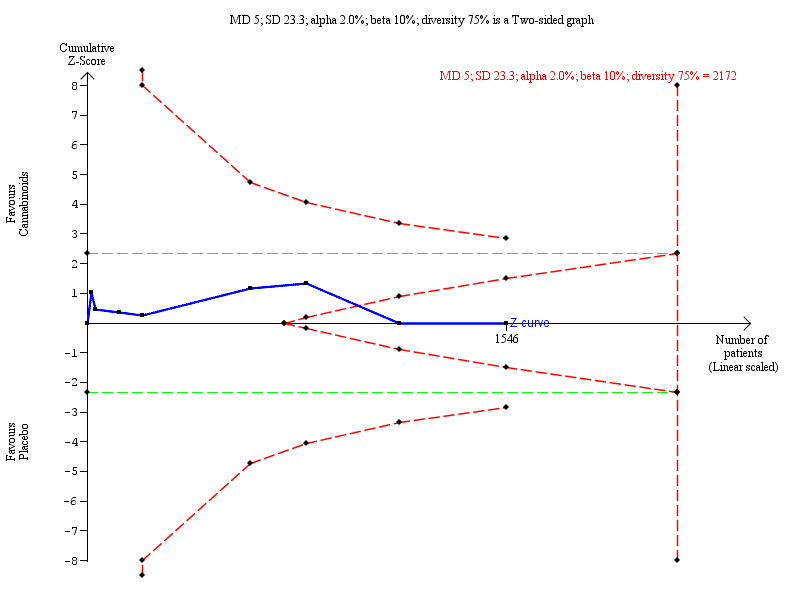


**Figure 49: Trial Sequential Analysis graph of 24 hour morphine consumption using fixed-effects meta-analysis.** Trial Sequential Analysis showed the z-curve (the blue line) breaching the boundary of futility, showing that there was enough information to reject that cannabinoids compared with placebo decreased the 24 hour morphine consumption by more than a mean difference of 5 mg.

**12 Physical function**


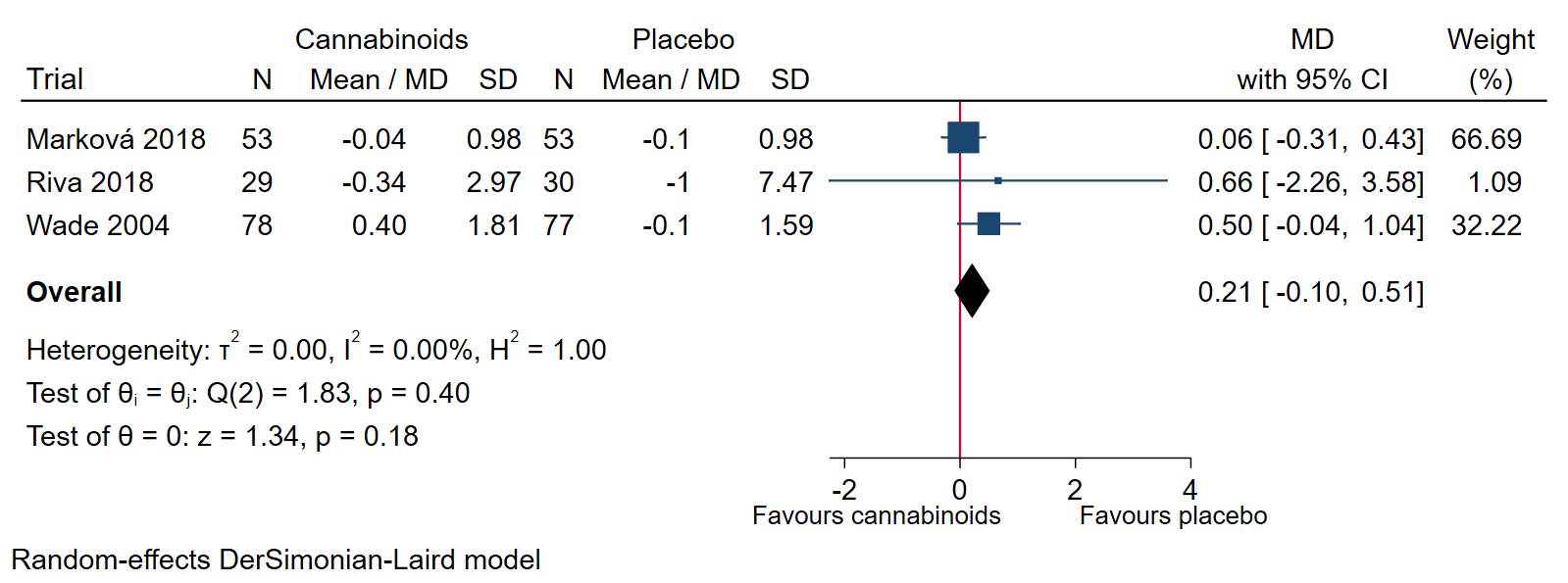


**Figure 50: Forest plot of the meta-analysis of physical function with 95% CI.** The meta-analysis of physical function showed no statistically significant evidence of a difference between cannabinoids and placebo. (SMD 0.18; 95% CI -0.04, 0.40; P= 0.10).

**13 Depressive symptoms**


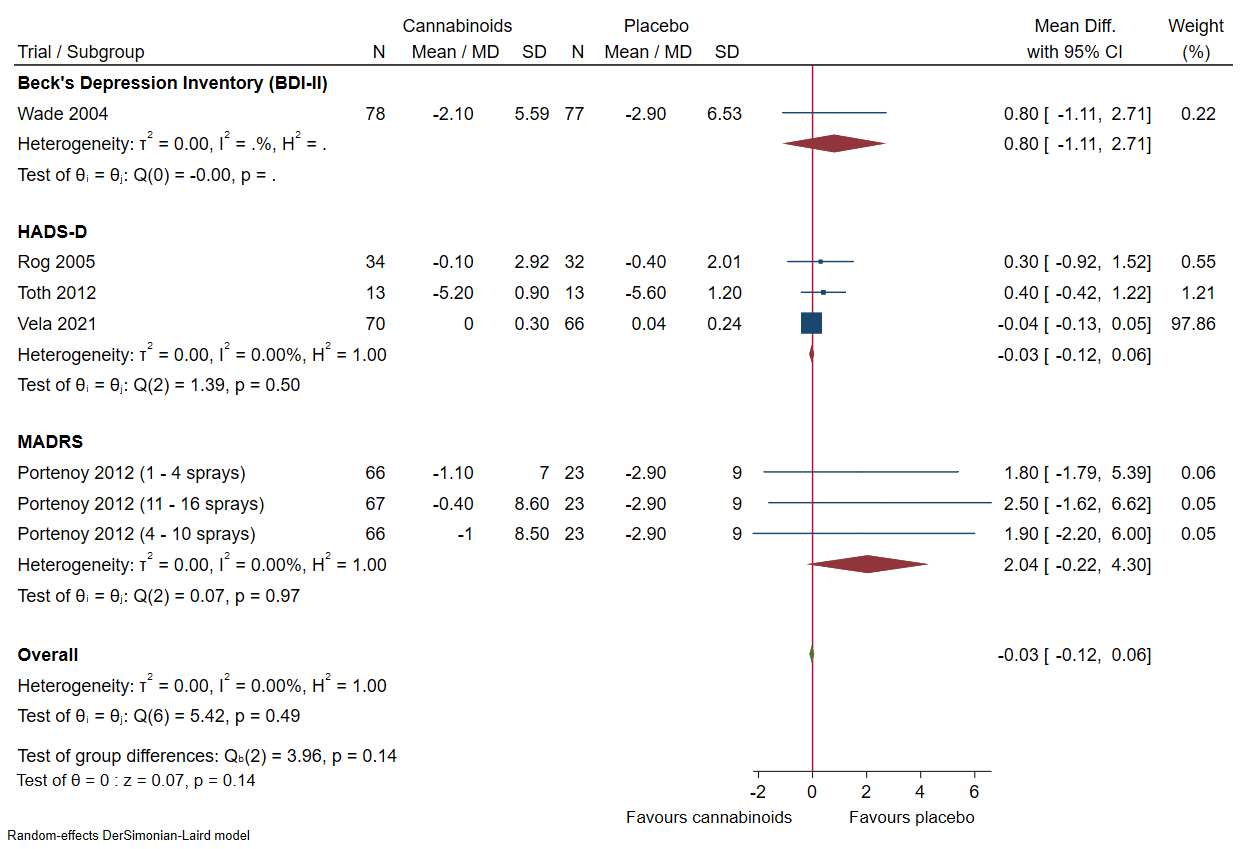


**Figure 51: Forest plot of the meta-analysis of depressive symptoms with 95% CI.** The meta-analysis of depressive symptoms showed no statistically significant evidence of a difference between cannabinoids and placebo. (SMD 0.19; 95% CI 0.01, 0.38; P= 0.04).


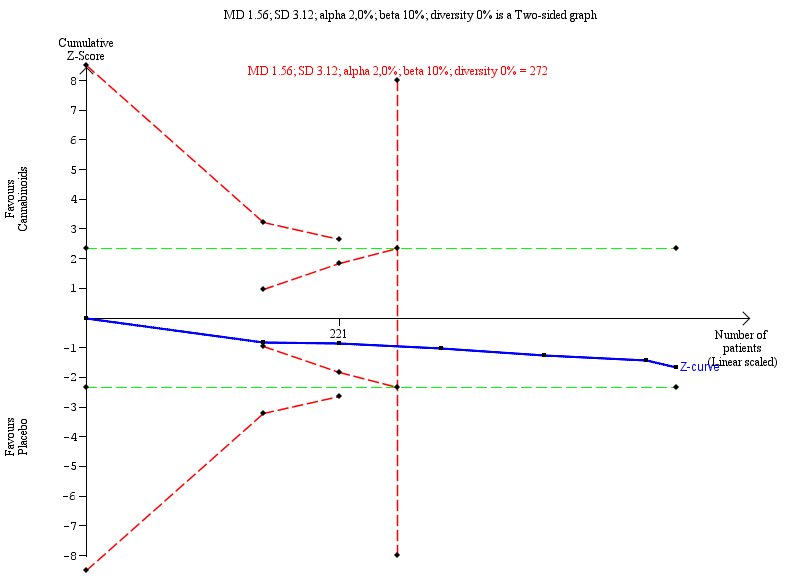


**Figure 52: Trial Sequential Analysis graph of depressive symptoms using random-effect meta-analysis.** Trial Sequential Analysis showed the z-curve (the blue line) breaching the boundary futility, showing that there was enough information to reject that cannabinoids compared with placebo decreased depressive symptoms.

| **14 Summary of findings:** | | | | | | |
| --- | --- | --- | --- | --- | --- | --- |
| **Cannabinoids compared with placebo for pain** | | | | | | |
| **Patient or population**: Pain  **Intervention**: Cannabinoids  **Comparison**: Placebo | | | | | | |
| Outcomes | **Anticipated absolute effects^*^** (95% CI) | | Relative effect (95% CI) | № of participants  (studies) | Certainty of the evidence (GRADE) | Comments |
|  | **Risk with Placebo** | **Risk with Cannabinoids** |  |  |  |  |
| All cause mortality follow up: mean 2 months | 89 per 1.000 | **107 per 1.000** (80 to 141) | **RR 1.20** (0.90 to 1.59) | 2073 (7 RCTs) | ⨁⨁◯◯ LOW ^a,b^ | DARIS = 13622 |
| Acute pain score difference assessed with: NRS |  | MD **0.52 Points higher** (0.25 lower to 1.29 higher) | - | 530 (4 RCTs) | ⨁◯◯◯ VERY LOW ^a,b,c^ | DARIS = 1207 |
| Cancer pain score difference assessed with: NRS |  | MD **0.13 Points lower** (0.30 lower to 0.03 higher) | - | 1550 (6 RCTs) | ⨁⨁◯◯ LOW ^a,b^ | DARIS = 154 |
| Chronic pain score difference assessed with: NRS |  | MD **0.43 Points lower** (0.72 lower to 0.15 lower) | - | 2030 (16 RCTs) | ⨁⨁◯◯ LOW ^a,d^ | DARIS = 390 |
| Serious adverse events assessed with: RR | 100 per 1.000 | **118 per 1.000** (99 to 141) | **RR 1.18** (0.95 to 1.45) | 3980 (18 RCTs) | ⨁⨁◯◯ LOW ^a,e^ | DARIS = 10720 |
| Quality of life Scale from: 0 to 100 follow up: mean 2 months |  | MD **1.38 points lower** (11.8 lower to 9.04 higher) | - | 548 (4 RCTs) | ⨁◯◯◯ VERY LOW ^a,b,c^ | DARIS = 1579 |
| Adverse events not considered serious assessed with: RR | 633 per 1.000 | **760 per 1.000** (728 to 791) | **RR 1.20** (1.15 to 1.25) | 5536 (29 RCTs) | ⨁◯◯◯ VERY LOW ^a,f,g^ | DARIS = 1407 |
| Quality of sleep assessed with: NRS follow up: mean 2 months |  | MD **0.42 points lower** (0.65 lower to 0.20 lower) | - | 3291 (17 RCTs) | ⨁⨁◯◯ LOW ^a,d^ | DARIS = 699 |
| Twenty four hour morphine consumption assessed with: mg |  | MD **0.01 mg lower** (1.60 lower to 1.59 higher) | - | 1546 (6 RCTs) | ⨁⨁◯◯ LOW ^a,h^ | DARIS = 2172 |
| Physical function (measured by activities of daily living) assessed with: Barthel index |  | MD **0.21 points higher** (0.1 lower to 0.51 higher) | - | 320 (3 RCTs) | ⨁◯◯◯ VERY LOW ^a,i^ | TSA could not be performed due to inadequate information size |
| Depressive symptoms assessed with: HADS-D, MADRS and BDI-II |  | MD **0.03 points lower** (0.12 lower to 0.06 higher) | - | 651 (5 RCTs) | ⨁⨁◯◯ LOW ^a,b^ | DARIS = 272 |
| ***The risk in the intervention group** (and its 95% confidence interval) is based on the assumed risk in the comparison group and the **relative effect** of the intervention (and its 95% CI).   **CI:** Confidence interval; **RR:** Risk ratio; **MD:** Mean difference; **DARIS**: Diversity adjusted required information size | | | | | | |
| **GRADE Working Group grades of evidence** **High certainty:** We are very confident that the true effect lies close to that of the estimate of the effect **Moderate certainty:** We are moderately confident in the effect estimate: The true effect is likely to be close to the estimate of the effect, but there is a possibility that it is substantially different **Low certainty:** Our confidence in the effect estimate is limited: The true effect may be substantially different from the estimate of the effect **Very low certainty:** We have very little confidence in the effect estimate: The true effect is likely to be substantially different from the estimate of effect | | | | | | |

#### Explanations

a. Trials were generally assessed as high risk of bias. If the majority of the included trials were assessed as high risk of bias the outcome was assessed as high risk of bias, as well.

b. The required information size recommended by the Trial Sequential Analysis was not achieved.

c. Visual inspection of the forest plot, I^2^ – statistic and Tau^2^ – statistic indicated high heterogeneity of the overall meta-analysis.

d. Visual inspection of the forest plot, I^2^ – statistic and Tau^2^ – statistic indicated moderate heterogeneity of the overall meta-analysis.

e. There was a broad variety of the definition of a serious adverse event.

f. There was suspicion of small study effects according to the funnel plot for this outcome.

g. There was a broad variety of the definition of a non-serious adverse event.

h. Morphine consumption is an indirect outcome of the analgesic effect of cannabinoids.

i. Trial Sequential Analysis could not be performed due inadequate information size.
