# Supplementary material for: Cannabinoids versus placebo for pain: A systematic review with meta-analysis and Trial Sequential Analysis
Source: PLoS One. 2023 Jan 30;18(1):e0267420. doi: 10.1371/journal.pone.0267420 (PMC9886264; doi:10.1371/journal.pone.0267420)
Supplement: S4 File — (DOCX) [file pone.0267420.s005.docx]

***Abrams 2007***

| **Methods** | Randomised clinical trial. Conducted in the USA |
| --- | --- |
| **Participants** | **Inclusion criteria**  "Patients were adults with HIV infection and symptomatic HIV-SN with an average daily pain score ofat least 30 mm on the 100 mm visual analog scale during the outpatient pre-intervention phase."  "All patients were required to have prior experience smoking cannabis (defined as six or more times in their lifetime), so that they would know how to inhale and what neuropsychologic effects to expect."  **Exclusion criteria**  None specified |
| **Interventions** | **Experimental intervention**  Active cannabis cigarettes contained 3.56% delta-9-tetrahydrocannabinol (delta-9-THC), which was somoked 3 times daily. Intervention lasted 5 days.  **Control Intervention** Identically looking placebo, smoked 3 times daily. |
| **Outcomes** | Primary outcome: Daily diary pain VAS.  Secondary outcomes: percent change in total mood disturbance and percent change in the six subscales of the Profile of Mood States |
| **Notes** |  |

Risk of bias table

| **Bias** | **Authors' judgement** | **Support for judgement** |
| --- | --- | --- |
| Random sequence generation (selection bias) | Low risk | "Randomization (1:1) to cannabis or placebo cigarettes was computer-generated by the study statistician and managed by an independent research pharmacist." |
| Allocation concealment (selection bias) | Low risk | "Randomization (1:1) to cannabis or placebo cigarettes was computer-generated by the study statistician and managed by an independent research pharmacist." |
| Blinding of participants and personnel (performance bias) | Low risk | "identically appearing pre-rolled cannabis and placebo cigarettes weighing on average 0.9 g" and the treatment was described as double blind. |
| Blinding of outcome assessment (detection bias) | Unclear risk | Not described. |
| Incomplete outcome data (attrition bias) | Unclear risk | "Statistical analyses were conducted on a modified intent-to treat (ITT) sample. All patients who remained in the study at each time point were included in the analyses."  The trial did not include all participants in the final analysis, more than 5% of participants had incomplete data and the trial did not use appropriate methods to handle missing data |
| Selective reporting (reporting bias) | Low risk | If there is no protocol or the protocol was published after the trial has begun, reporting pain assessment on VAS or NRS and serious adverse events will grant the trial a grade of low risk of bias. |
| Other bias | Low risk | The trial appeared free of other types of bias. |

***Abrams 2020***

| **Methods** | Randomised placebo-controlled crossover trial |
| --- | --- |
| **Participants** | Inclusion Criteria:   - Sickle cell disease, including sickle cell anemia (SS), sickle-hemoglobin C disease (SC), and sickle beta thalassemia disease (Sb). - Ongoing opioid analgesic therapy for chronic sickle cell disease-associated pain. - Subjects must be on a stable dose of analgesic medication (opioid or other) for at least 2 weeks before enrollment. - All men and women in this study must agree to use adequate birth control during this study. Acceptable barrier birth control methods are a male condom, female condom, diaphragm, or intra-uterine (IUD). - All women of reproductive potential (who have not reached menopause or undergone hysterectomy, oophorectomy, or tubal ligation) must have a negative urine b-HCG pregnancy test performed before initiating the protocol-specified medication. - Prior history of use of cannabis. Subjects must have smoked cannabis on at least 6 occasions in their lifetime prior to enrollment. - Subjects will self-report abstaining from smoking or ingesting cannabis for one week prior to their enrollment into the study. - Able to understand and follow the instructions of the investigator, including completing the pain intensity rating scales. - Karnofsky Performance Scale >60. - Able and willing to provide informed consent. - Able and willing to spend two separate periods of 5 days and 4 nights in the Clinical Research Center at SFGH.   Exclusion Criteria:   - Severe coronary artery disease, uncontrolled hypertension, cardiac ventricular conduction abnormalities, orthostatic mean blood pressure drop greater than 24 mmHg, severe chronic obstructive pulmonary disease. - Evidence of clinically significant hepatic or renal dysfunction based on judgment of physician. - Positive serum THC level on Day 1 of study. - Active substance abuse (e.g., alcohol or injection drugs) as determined by urine toxicity screening. - Neurologic dysfunction or psychiatric disorder severe enough to interfere with assessment of pain or sensory systems. - Current use of smoked tobacco products. - Women who are pregnant or breast-feeding may not take part in this study. - Unable to read or speak English. |
| **Interventions** | Experimental: Cannabis  Contents of 1 cannabis cigarette (4.7% THC/5.1% CBD) will be vaporized and inhaled at 12pm on Day 1; 8am, 2pm and 8pm on Days 2-4; and 8am on Day 5.  Placebo Comparator: Placebo  Contents of 1 placebo cigarette (0% THC/0% CBD) will be vaporized and inhaled at 12pm on Day 1; 8am, 2pm and 8pm on Days 2-4; and 8am on Day 5.  Each participant was exposed to a period of 5 inpatient days of either vaporized cannabis or vaporized placebo, then a month-long washout period out of the hospital, followed by a second 5-day admission with the opposite treatment. Thus, each participant was randomly assigned to the sequence active-placebo (indicating active drug in the first period and placebo in the second period), or placebo-active (indicating placebo in the first period and active drug in the second period). |
| **Outcomes** | Primary Outcome Measures  :   1. Pain Rating Using Visual Analog Scale at Day 1 and Day 5 [ Time Frame: Day 1 and Day 5 ]Visual analog scale (VAS) used to assess pain. The scale range is 0-100, lower score means lower pain, higher score means higher pai |
| **Notes** | ClinicalTrials.gov Identifier:  [NCT01771731](https://clinicaltrials.gov/ct2/show/NCT01771731) |

Risk of bias table

| **Bias** | **Authors' judgement** | **Support for judgement** |
| --- | --- | --- |
| Random sequence generation (selection bias) | Low risk | Randomization order of cannabis and placebo was computer-generated by the study statistician and managed by an independent research pharmacist. |
| Allocation concealment (selection bias) | Unclear risk | No described clearly |
| Blinding of participants and personnel (performance bias) | Low risk | Treatment was double-blind. |
| Blinding of outcome assessment (detection bias) | Unclear risk | Not described clearly |
| Incomplete outcome data (attrition bias) | Unclear risk | There was insufficient information to assess whether missing data were likely to induce bias on the results. |
| Selective reporting (reporting bias) | Low risk | This trial is registered with ClinicalTrials.gov, number NCT01771731 |
| Other bias | Low risk | his work was funded by grant No. UO1HL117664 from the National Institutes of Health (NIH) National Heart, Lung and Blood Institute (Dr Gupta). The Zuckerberg San Francisco General Clinical Research Center was funded award No. UL1TR000004 from the NIH National Center for Advancing Translational Sciences. |

***Almog 2020***

| **Methods** | Randomised, three‐arms, double‐blind, placebo‐controlled, cross‐over study |
| --- | --- |
| **Participants** | Inclusion criteria:   1. Adult patients (18 years of age or above), 2. Capable of giving informed consent, 3. Suffering from chronic pain with a baseline pain intensity of 6 or above on a 10-cm visual analog scale (VAS), 4. Licensed by the Israeli Ministry of Health to receive cannabis-based medicines. 5. Active users had to agree to abstain from cannabis-based medicines 12 hr before study intervention. 6. Women of fertile age had to declare using con-traception.   Exclusion criteria:  Had a history of severe, uncontrolled cardiac disease, pulmonary disease, hepatic disease, neurologic dysfunction.  Personal or family history of psychotic illness, substance abuse, or if they were pregnant, breastfeeding, or did not use adequate birth control |
| **Interventions** | A single inhalation of 9-THC: 0.5mg, 1mg, or a placebo. |
| **Outcomes** | The primary objectives of the study were:  (I) studying the Δ^9^‐THC pharmacokinetics following administration of a single inhalation of 0.5 mg and 1.0 mg aerosolized Δ^9^‐THC compared to a placebo, using the meter‐dosed Syqe Inhaler  (II) evaluating their analgesic efficacy (change in pain intensity from baseline) in patients with chronic pain.  The secondary objectives were  Study the safety and tol-erability of two doses of aerosolized medical cannabis and a placebo, over a post inhalation period of 150min |
| **Notes** |  |

Risk of bias table

| **Bias** | **Authors' judgement** | **Support for judgement** |
| --- | --- | --- |
| Random sequence generation (selection bias) | Unclear risk | Not described |
| Allocation concealment (selection bias) | Unclear risk | Not described |
| Blinding of participants and personnel (performance bias) | Unclear risk | Not described |
| Blinding of outcome assessment (detection bias) | Unclear risk | Not described |
| Incomplete outcome data (attrition bias) | Low risk | Safety analysis was performed using the intention-to-treat (ITT) population, which included all study patients. |
| Selective reporting (reporting bias) | Unclear risk | If no protocol was published and the outcome pain assessment on VAS or NRS and serious adverse events were not reported on. |
| Other bias | High risk | Funding or sponsor not mentioned |

***Aronow 1974***

| **Methods** | Randomised trial, crossover design. Conducted in the USA. |
| --- | --- |
| **Participants** | **Inclusion criteria**  Ten men who had classic stable exertional angina pectoris and angiographic evidence of severe coronary-artery disease with >75 per cent narrowing of at least one major coronary vessel.  **Exclusion criteria:**  No stated exclusioncriteria. |
| **Interventions** | **Experimental intervention**  Each patient smoked on two mornings one marihuana cigarette. The subjects inhaled 10 puffs of smoke.The experimental cigarette contained 19.8 mg of delta-9-THC.  **Control intervention**  Two mornings of consuming one placebo marihuana cigarette. The subjects inhaled 10 puffs of smoke. The control cigarette contained 0.05 mg of delta-9-THC. |
| **Outcomes** | Heart rate, blood pressure, venous carboxyhemoglobin, exercise performance. |
| **Notes** | Participants received both the experimental and control intervention on alternating days. No contactinformation could be found. |

Risk of bias table

| **Bias** | **Authors' judgement** | **Support for judgement** |
| --- | --- | --- |
| Random sequence generation (selection bias) | Unclear risk | Not described |
| Allocation concealment (selection bias) | Unclear risk | Not described |
| Blinding of participants and personnel (performance bias) | Low risk | The study was described as double blinded. The aroma from the placebocigarette was described as being similar to the experimental intervention |
| Blinding of outcome assessment (detection bias) | Unclear risk | Not described |
| Incomplete outcome data (attrition bias) | Unclear risk | Not described |
| Selective reporting (reporting bias) | Unclear risk | No protocol could be obtained and trial did not report serious adverse events |
| Other bias | Unclear risk | The trial appeared free of other bias |

***Beaulieu 2006 (1 mg)***

| **Methods** | A double-blind, Randomised, placebo-controlled, parallel-group pilot trial. Conducted from November 2003 to April 2005 in Canada. |
| --- | --- |
| **Participants** | **Inclusion criteria**  Patients of the American Society of Anesthesiologists I, II or III; aged 18 to 75 yr, and scheduled for major surgery, using a PCA device postoperatively. Furthermore, a urine test for the detection of Δ9-THC was performed on the day of the operation to ensure that patients had not been recently under the influence of cannabis.  **Exclusion criteria** Patients using cannabis or subject to other substance abuse; alcoholics; patients where morphine was not the drug of choice for PCA; and patients with planned concomitant medication during the study with any of the following: non-steroidal anti-inflammatory drugs, acetaminophen, more than 300 mg acetylsalicylic acid per day, sedatives, anticonvulsants or antidepressants. Finally, patients with ischemic heart disease, cardiac arrhythmia or cardiac failure, patients with a history of gastric or duodenal ulcers, renal insufficiency or asthma, and patients with chronic pain conditions and/or receiving chronic opioid therapy, patients with a history of psychiatric illnessand pregnant or lactating women were also excluded. |
| **Interventions** | **Experimental**  Group 1: 1 mg nabilone (n = 11)  Group 2: 2 mg nabilone (n = 9)  Group 3: 50 mg ketoprofen (n = 11)  All interventions were given at given at eight-hour intervals for 24 hr  **Control**  Group 4: Placebo given at eight-hour intervals for 24 hr |
| **Outcomes** | The primary outcome was cumulative (24 hr) morphine consumption via morphine patient-controlled analgesia (PCA).  Secondary outcomes consisted of the following: pain intensities at rest and on movement; anti-emetic properties of nabilone; assessment of mood and euphoria; quality of sleep and incidence of side effects such as sedation and psychotic episodes  Serious adverse events were assessed. |
| **Notes** | We did not use data from group 3. The authors were contacted by email: pierre.beaulieu@umontreal.ca to ask for additional information to assess the risk of bias. |

Risk of bias table

| **Bias** | **Authors' judgement** | **Support for judgement** |
| --- | --- | --- |
| Random sequence generation (selection bias) | Low risk | "The randomization list (random blocks) was generated by a computer program which assigned each patient a number in one of the four different treatment groups." |
| Allocation concealment (selection bias) | Unclear risk | Not described |
| Blinding of participants and personnel (performance bias) | Low risk | "The two capsules administered to the patients were identically-appearing capsules given´ to ward nurses with patients’ name and time of administration written on it. Ward and research nurses were therefore completely blinded to the study medication delivered to the patients." |
| Blinding of outcome assessment (detection bias) | Unclear risk | "The two capsules administered to the patients were identically-appearing capsules given´ to ward nurses with patients’ name and time of administration written on it. Ward and research nurses were therefore completely blinded to the study medication delivered to the patients."  "A data and safety monitoring board was established, its principal mandate being to review adverse event reports, but also with the responsibility of reviewing dropouts, monitoring data collection, and protocol violations."  It was unclear whether the data and safety monitoring board was blinded. |
| Incomplete outcome data (attrition bias) | High risk | "In case of a missing value, this was replaced by the last previous valid value (“last value carried forward option”)." |
| Selective reporting (reporting bias) | Low risk | The trial reported serious adverse events and pain. |
| Other bias | Low risk | The trial appeared free of other bias |

***Beaulieu 2006 (2 mg)***

| **Methods** | A double-blind, Randomised, placebo-controlled, parallel-group pilot trial. Conducted from November 2003 to April 2005 in Canada. |
| --- | --- |
| **Participants** | **Inclusion criteria**  Patients of the American Society of Anesthesiologists I, II or III; aged 18 to 75 yr, and scheduled for major surgery, using a PCA device postoperatively. Furthermore, a urine test for the detection of Δ9-THC was performed on the day of the operation to ensure that patients had not been recently under the influence of cannabis.  **Exclusion criteria** Patients using cannabis or subject to other substance abuse; alcoholics; patients where morphine was not the drug of choice for PCA; and patients with planned concomitant medication during the study with any of the following: non-steroidal anti-inflammatory drugs, acetaminophen, more than 300 mg acetylsalicylic acid per day, sedatives, anticonvulsants or antidepressants. Finally, patients with ischemic heart disease, cardiac arrhythmia or cardiac failure, patients with a history of gastric or duodenal ulcers, renal insufficiency or asthma, and patients with chronic pain conditions and/or receiving chronic opioid therapy, patients with a history of psychiatric illnessand pregnant or lactating women were also excluded. |
| **Interventions** | **Experimental**  Group 1: 1 mg nabilone (n = 11)  Group 2: 2 mg nabilone (n = 9)  Group 3: 50 mg ketoprofen (n = 11)  All interventions were given at given at eight-hour intervals for 24 hr  **Control**  Group 4: Placebo given at eight-hour intervals for 24 hr |
| **Outcomes** | The primary outcome was cumulative (24 hr) morphine consumption via morphine patient-controlled analgesia (PCA).  Secondary outcomes consisted of the following: pain intensities at rest and on movement; anti-emetic properties of nabilone; assessment of mood and euphoria; quality of sleep and incidence of side effects such as sedation and psychotic episodes  Serious adverse events were assessed. |
| **Notes** | We did not use data from group 3. The authors were contacted by email: pierre.beaulieu@umontreal.ca to ask for additional information to assess the risk of bias. |

Risk of bias table

| **Bias** | **Authors' judgement** | **Support for judgement** |
| --- | --- | --- |
| Random sequence generation (selection bias) | Low risk | "The randomization list (random blocks) was generated by a computer program which assigned each patient a number in one of the four different treatment groups." |
| Allocation concealment (selection bias) | Unclear risk | Not described |
| Blinding of participants and personnel (performance bias) | Low risk | "The two capsules administered to the patients were identically-appearing capsules given´ to ward nurses with patients’ name and time of administration written on it. Ward and research nurses were therefore completely blinded to the study medication delivered to the patients." |
| Blinding of outcome assessment (detection bias) | Unclear risk | "The two capsules administered to the patients were identically-appearing capsules given´ to ward nurses with patients’ name and time of administration written on it. Ward and research nurses were therefore completely blinded to the study medication delivered to the patients."  "A data and safety monitoring board was established, its principal mandate being to review adverse event reports, but also with the responsibility of reviewing dropouts, monitoring data collection, and protocol violations."  It was unclear whether the data and safety monitoring board was blinded. |
| Incomplete outcome data (attrition bias) | High risk | "In case of a missing value, this was replaced by the last previous valid value (“last value carried forward option”)." |
| Selective reporting (reporting bias) | Low risk | The trial reported serious adverse events and pain. |
| Other bias | Low risk | The trial appeared free of other bias |

***Bebee 2021***

| **Methods** | The CANBACK trial was a single centre, randomised, double blinded, placebo-controlled clinical trial. |
| --- | --- |
| **Participants** | **Inclusion criteria**: People aged 18 years or more who pre- sented with acute non-traumatic low back pain — that is, pain and discomfort localised below the cos- tal margin and above the inferior gluteal folds, as assessed by the treating physician — of less than 30 days’ duration.  **Exclusion criteria**: People who reported using cannabis or CBD in the preceding seven days, those with abnormal neurological ex- amination findings (apart from subjective sensory changes), fever (exceeding 37.6°C), a history of malig- nancy, or a non-musculoskeletal cause of back pain, and women who were pregnant (urinary β-human chorionic gonadotropin testing of all women under 60 years of age). |
| **Interventions** | Patients received either the placebo (control group) or CBD (intervention group) (online Supporting Information). CBD (> 99.9% synthetic) 400 mg and placebo were purchased as colour-matched medications from GD Pharma (South Australia). |
| **Outcomes** | **The primary outcome** measure was verbal numerical pain score (range, 0–10) two hours after administration of CBD or placebo.  **Secondary outcome** measures were emergency department length of stay (including in the short stay unit), need for rescue analgesia, and any adverse event. |
| **Notes** |  |

Risk of bias table

| **Bias** | **Authors' judgement** | **Support for judgement** |
| --- | --- | --- |
| Random sequence generation (selection bias) | Low risk | Consenting participants were randomised 1:1 to the intervention and control groups. We used an online permutation generator (twjc.co.uk/combinations. html) to generate permutation blocks (six patients per block); the blocks were then randomised (using the generator at random.org) to provide the final allocation list (maximum, 120 patients). |
| Allocation concealment (selection bias) | Low risk | The study syringes were numbered consecutively by pharmacy staff and strictly allocated to patients as they were recruited. A list with the treatment break code was securely stored in the hospital pharmacy. The allocation sequence was generated and study medications prepared by a clinical trials pharmacist not involved in study design or re- cruitment. The content of each treatment pack (CBD or placebo) was known only to central hospital pharmacy staff. |
| Blinding of participants and personnel (performance bias) | Low risk | The treating clinicians, nursing staff, investigators, and participants were blinded to the study intervention. |
| Blinding of outcome assessment (detection bias) | Low risk | The treating clinicians, nursing staff, investigators, and participants were blinded to the study intervention. |
| Incomplete outcome data (attrition bias) | Low risk | Used intention-to treat approach |
| Selective reporting (reporting bias) | Low risk | The trial was registered with the Australian New Zealand Clinical Trials Registry on 4 April 2018 (ACTRN12618000487213). |
| Other bias | Low risk | The trial appeared free of other types of bias. |

***Berman 2004***

| **Methods** | Single centre, double-blind, randomised, placebo-controlled, three period crossover study. Conducted between December 2001 and July 2002 at the Royal National Orthopaedic Hospital (RNOH) in Stanmore, UK. |
| --- | --- |
| **Participants** | **Inclusion criteria**  Men and women aged 18 years or more, with at least one avulsed brachial plexus root and with the injury occurring R18 months previously were included. Patients who scored four or above on a zero to 10 eleven point ordinal pain severity scale at Visits 1 and 2 and had a pattern of pain that in the investigator’s opinion had been stable over the previous 4 weeks were eligible. All permitted concurrent medication had to have been stable during the previous 4 weeks and was to remain stable during the study.  **Exclusion criteria**  Patients with a history of any of the following were ineligible: schizophrenia, other psychotic illness or significant psychiatric illness, other than depression associated with chronic illness; serious cardiovascular disease; significant renal or hepatic impairment; epilepsy or convulsions; significant history of substance abuse; known adverse reaction to cannabis or the product excipients; surgery within 2 months (6 months for nerve repair). Female patients who were pregnant, lactating or at risk of pregnancy were also excluded. |
| **Interventions** | **Experimental**  Group 1: GW-1000-02 (Sativex) containing D9tetrahydrocannabinol (THC):cannabidiol (CBD) in an approximate 1:1 ratio (27 mg/ml THC and 25 mg/ml CBD pr. spray).  Group 2: GW-2000-02 containing only THC (27 mg/ml THC)  **Control**  Placebo  **Co-intervention**  Patients were instructed on how to self-medicate and were monitored in clinic over 4 h, on the dosing day at the start of each period, while they took up to four initial doses. Patients returned for their next visit 2 weeks later for end of period assessments and to receive the next medication. This process was repeated at a fourth visit, 2 weeks later. Two weeks after this, patients attended for a fifth and final visit that included. Depending upon their clinical response to the dose of study medication given in the clinic each patient was advised to take between four and eight sprays the next day. Patients were instructed not to increase their total daily number of sprays by more than 50% of the number taken during the previous 24 h. |
| **Outcomes** | Pain on Box Scale-11, sleep quality on Box Scale-11, SLeep disturbances as a catagorical variable, The short form McGill questionnaire (SF-MPQ), Pain Disability Index (PDI) and General Health Questionnaire-12 (GHQ-12), adverse events, VAS. |
| **Notes** | We could not use the data from the trial publication, as it did not report the data from the first period before crossover, as dictated by our protocol. The trial author was contacted by email: jonathan.berman@rnoh.nhs.uk on 06.12.2019 asking for: data from before participants crossed over including the number of actual participants with data at this time point and if any imputation such as last observation carried forward were used, If both participants, treatment providers and outcome assessors were blinded and how this blinding was ensured (trial described as double-blinded), if there was a pre-published protocol. |

Risk of bias table

| **Bias** | **Authors' judgement** | **Support for judgement** |
| --- | --- | --- |
| Random sequence generation (selection bias) | Low risk | Patients were randomly allocated by a computer generated list |
| Allocation concealment (selection bias) | Unclear risk | It was not described how the randomisation list was kept concealed. |
| Blinding of participants and personnel (performance bias) | Unclear risk | The trial was described as double blinded but it was not described who was blinded and how the blinding was maintained. |
| Blinding of outcome assessment (detection bias) | Unclear risk | The trial was described as double blinded but it was not described who was blinded and how the blinding was maintained. |
| Incomplete outcome data (attrition bias) | Unclear risk | The following statements support a low risk of bias for incomplete outcome data but is not sufficient for us to determine: "Data were therefore available for 48 patients who received placebo, 46 who received GW-1000-02 and 47 who received GW-2000-02." "The intention to treat population consisted of all the patients who entered into the study, were randomised and had on treatment study data collected for at least 3 days from a treatment period". "Any patients who received at least one dose of study drug were included in the safety analysis population." |
| Selective reporting (reporting bias) | Low risk | There was no indication of a pre published protocol but the trial reported adverse events and VAS. |
| Other bias | Low risk | The trial appeared free of other bias |

***Blake 2006***

| **Methods** | Multicentre, double-blind, randomised, parallel-group trial. |
| --- | --- |
| **Participants** | **Inclusion**  Eligible patients had a diagnosis of RA meeting ACR criteria, with active arthritis not adequately controlled by standard medication. NSAID and prednisolone regimes had to have been stabilized for 1 month and DMARDs for 3 months prior to enrolment, and were maintained constant throughout the study.  **Exclusion**  Exclusion criteria included a history of psychiatric disorders or substance misuse, severe cardiovascular, renal or hepatic disorder, or a history of epilepsy. |
| **Interventions** | **Experimental**  CBM (Sativex). Sativex was administered by oromucosal spray, each activation delivering 2.7 mg THC and 2.5 mg CBD. The maximum number of actuations were 6.  **Control**  Placebo. The maximum number of actuations were 6. |
| **Outcomes** | Primary outcome: NRS.  Secondary outcomes included NRS measures of pain at rest, sleep quality and morning stiffness, the Short-Form McGill Pain Questionnaire (SF-MPQ) and the 28-joint disease activity score (DAS28). |
| **Notes** | Author was contacted by email: David.Blake@rnhrd-tr.swest.nhs.uk on 20.12.2020 to ask for the information needed to assess bias risk domains. |

Risk of bias table

| **Bias** | **Authors' judgement** | **Support for judgement** |
| --- | --- | --- |
| Random sequence generation (selection bias) | Unclear risk | Not described |
| Allocation concealment (selection bias) | Unclear risk | Not described |
| Blinding of participants and personnel (performance bias) | Low risk | The trial was described as double blind and the trial used a placebo. |
| Blinding of outcome assessment (detection bias) | Unclear risk | Not described |
| Incomplete outcome data (attrition bias) | Unclear risk | More than 5% withdrew from the study (3 in placebo, 1 in the active group) and it was unclear which participants had complete data. |
| Selective reporting (reporting bias) | Low risk | There was no prepublished protocol. The trial reported pain and serious adverse events. |
| Other bias | Low risk | The trial appeared free of other types of bias. |

***Buggy 2004***

| **Methods** | Randomised, parallel group, double-blind. Conducted at Leicester Royal Infirmary and Leicester General Hospital, UK. |
| --- | --- |
| **Participants** | **Inclusion**  American Society of Anesthesiologists classification I or II) scheduled for elective total abdominal hysterectomy  **Exclusion criteria:**  Patients with a history of drug or alcohol abuse, previous cannabis use and past or present psychiatric illness were excluded. |
| **Interventions** | **Experimental**  oral d-9-THC 5 mg started on the 2nd day post operation.  **Control**  Placebo  **Co-intervention**  Patient-controlled analgesia (PCA) using morphine (bolus dose 1 mg, lockout-time 5 min) was provided for the first 24 postoperative hours in all patients. |
| **Outcomes** | "The primary outcome measure was summed pain intensity difference (SPID) at 6 h after administration of study medication derived from visual analogue pain scores on movement and at rest. Secondary outcome measures were time to rescue medication and adverse effects of study medication." |
| **Notes** | The author was contacted regarding the missing information to assess bias as well as missing outcomes on email: djr8@le.ac.uk on date XX. |

Risk of bias table

| **Bias** | **Authors' judgement** | **Support for judgement** |
| --- | --- | --- |
| Random sequence generation (selection bias) | Unclear risk | Not described |
| Allocation concealment (selection bias) | Unclear risk | Not described |
| Blinding of participants and personnel (performance bias) | Low risk | single, identical capsule and trial was described as double blinded. |
| Blinding of outcome assessment (detection bias) | Unclear risk | single, identical capsule, but it was not described if the outcome assessors were blinded |
| Incomplete outcome data (attrition bias) | Low risk | "No patient withdrew from the study after randomization." It appears the trial had full data on all participants. |
| Selective reporting (reporting bias) | Low risk | The trial reported pain on VAS and adverse events |
| Other bias | Unclear risk | The trial appeared free of other bias |

***Collin 2010***

| **Methods** | Randomised, parallel group, double-blind. Conducted at 15 centers in the UK and eight in the Czech Republic |
| --- | --- |
| **Participants** | **Inclusion criteria**  Eligible subjects had any disease subtype of MS of at least 6-months duration, and at least a 3-month history of spasticity due to MS, which was not wholly relieved with current therapy.  During the last 6 days of the baseline period, these scores had to sum to at least 24 (i.e. subjects had to have a minimum mean daily score of 4 out of 10: moderate spasticity) to be eligible to participate.  **Exclusion criteria**  Subjects with symptoms of spasticity not due to MS were excluded. Any person with a concurrent history of significant psychiatric, renal, hepatic, cardiovascular or convulsive disorders was also excluded due to lack of knowledge of the tolerability of cannabinoids in these scenarios. |
| **Interventions** | **Experimental intervention**  Study medication was delivered using a pump action oromucosal spray. Each 100 ml actuation of active medication delivered a dose containing 2.7 mg THC and 2.5 mg CBD (Sativex). The maximum permitted dose of study medication was eight actuations in any 3-hour period, and 24 actuations in any 24-hour period.  **Control intervention**  Study medication was delivered using a pump action oromucosal spray. Each actuation of placebo delivered 100 ml of vehicle containing excipients plus colourants. The maximum permitted dose of study medication was eight actuations in any 3-hour period, and 24 actuations in any 24-hour period |
| **Outcomes** | "The primary endpoint was a spasticity 0–10 numeric rating scale (NRS)"  Secondary: responders, functional assessment (10-meter walk test, the Barthel activity of Daily living index and caregiver’s global impression of change (CGIC)), rating ease of; transfer, dressing and perineal hygiene, the modified Ashworth scale, an ordinal scale that measures resistance to passive movement, was included together with two quality of life scales, the EQ-5D and MSQoL-54 |
| **Notes** | Number of participants with serious adverse events in the control group, how was the allocation sequence generated, how was it concealed, were outcome assessors blinded, was there a pre-published protocol, standard deviation for NRS, [christine.collin@royalberkshire.nhs. uk] |

Risk of bias table

| **Bias** | **Authors' judgement** | **Support for judgement** |
| --- | --- | --- |
| Random sequence generation (selection bias) | Unclear risk | Not described |
| Allocation concealment (selection bias) | Unclear risk | Not described |
| Blinding of participants and personnel (performance bias) | Low risk | Identical placebo |
| Blinding of outcome assessment (detection bias) | Unclear risk | Not described |
| Incomplete outcome data (attrition bias) | Unclear risk | The trial seemed to have above 5% of participants without full data at the end of the trial and the trial did not specify how it handled missing data, although it used a modified form of intention to treat. |
| Selective reporting (reporting bias) | Unclear risk | The trial did not report serious adverse events for the control group and no pre-published protocol was available. |
| Other bias | Low risk | The trial appeared free of other bias |

***Colwill 2020***

| **Methods** | Randomised, double-blind, placebo-controlled trial |
| --- | --- |
| **Participants** | Inclusion Criteria:   - Aged 21 years or older - Consented for elective medical abortion - Pregnancy with intrauterine gestational sac up to 10 0/7 weeks, dated by ultrasound - Able and willing to receive text messages via phone - English speaking - Able and willing to give informed consent and agree to the study terms - Have assistance at home; no motor vehicle use while taking study medications   Exclusion Criteria:   - Desires to continue pregnancy or currently breastfeeding - Lack of access to cell phone and texting capabilities - Prior participation in this study - Early pregnancy failure - Contraindications to the study medications: Marinol or marijuana derivatives, sesame oil, Ibuprofen - Contraindications to medical abortion with Mifepristone or Misoprostol - History of methadone, buprenorphine or heroin use within the last year - History of a seizure disorder - Used marijuana 5 or more days in the last week - History of any adverse effects associated with prior use of recreational or medical marijuana products, or sensitivity/allergy to Marinol. |
| **Interventions** | Experimental: Gestational age up to 10w0d - Dronabinol  Women with gestational age up to 10 weeks 0 days will be Randomised to Dronabinol 5mg Cap or placebo  Placebo Comparator: Gestational age up to 10w0d - Placebo  Women with gestational age up to 10 weeks 0 days will be Randomised to Dronabinol 5mg Cap or placebo |
| **Outcomes** | **Primary Outcome Measures:**   1. Maximum Self-reported Pain Score on a Numeric Rating Scale [ Time Frame: 24 hours after misoprostol administration ]Women will text responds to surveys within 24 hours after misoprostol administration indicating their maximum self-reported pain using an 11-point numeric rating scale (NRS 0-10) where 0=no pain and 10=worst possible pain.   **Secondary Outcome Measures:**   1. Pain scores 2. Maximum anxiety 3. Nausea score 4. Use of additional pain medication 5. Reported side effects 6. Self-assessment of study arm assignment 7. Satisfaction with pain management |
| **Notes** | Sponsors and Collaborators Oregon Health and Science University Society of Family Planning |

Risk of bias table

| **Bias** | **Authors' judgement** | **Support for judgement** |
| --- | --- | --- |
| Random sequence generation (selection bias) | Low risk | Oregon Health & Research University Research Pharmacy staff generated, assigned, and maintained the computer generated 1:1 randomization scheme of blocks of 8 or 10. |
| Allocation concealment (selection bias) | Low risk | The study drugs, dronabinol or identically matched placebo, were labelled in identical sequentially numbered packets. |
| Blinding of participants and personnel (performance bias) | Low risk | Study staff, participants, and care providers were blinded to treatment assignments; the randomization sequence was unblinded only after completion of all data collection and entry |
| Blinding of outcome assessment (detection bias) | Low risk | Study staff, participants, and care providers were blinded to treatment assignments; the randomization sequence was unblinded only after completion of all data collection and entry |
| Incomplete outcome data (attrition bias) | Low risk | Used intention-to treat approach and analyzed all Randomised women |
| Selective reporting (reporting bias) | Low risk | This trial is registered with ClinicalTrials.gov, number NCT03604341 |
| Other bias | Low risk | Sponsors and Collaborators Oregon Health and Science University Society of Family Planning |

***Conte 2009***

| **Methods** | Randomised, crossover, double-blind study. |
| --- | --- |
| **Participants** | **Inclusion criteria** stable disease and no systemic corticosteroid therapy for at least 30 days before study entry; lower-limb spasticity; antispastic- agents (dose, frequency and route of administration) stable for at least 4 weeks before study entry.  **Exclusion criteria**  no disease modifying therapies prescribed in the 6 months before study; no history of epilepsy of alcohol or substance abuse (cannabis-naı¨ve patients) and no major medical illnesses including renal, hepatic, cardiac, active thyroid disease, or diabetes mellitus; no psychiatric or cognitive impairment that precluded safe participation in the study; and no concomitant therapy with antidepressants, psychoactive drugs or corticosteroids. We also excluded female patients who were pregnant, lactating or planning pregnancy during the course of the study. Participants had to be willing to comply with the protocol throughout the study. Patients suffering from acute or chronic pain were excluded because pain might increase spasticity. |
| **Interventions** | **Experimental intervention**  Each cannabinoid oromucosal spray delivered 2.7 mg of THC and 2.5 mg of CBD.The maximum permitted dose was 8 sprays in 3 hours and a total dose of 48 sprays over 24 hours.  **Control intervention**  Each placebo spray delivered excipients plus colorants. The maximum permitted dose was 8 sprays in 3 hours and a total dose of 48 sprays over 24 hours |
| **Outcomes** | Pain on VAS, serious adverse events, adverse events. |
| **Notes** | EUDRACT number 2005-000393-47. The authors were contacted by email: maurizio.inghilleri@uniroma1.it for data at the end of the wash out period (5 weeks). We only used data on serious adverse events. |

Risk of bias table

| **Bias** | **Authors' judgement** | **Support for judgement** |
| --- | --- | --- |
| Random sequence generation (selection bias) | Unclear risk | Not described |
| Allocation concealment (selection bias) | Unclear risk | Not described |
| Blinding of participants and personnel (performance bias) | Low risk | Described as double blind and used Identical placebo |
| Blinding of outcome assessment (detection bias) | Unclear risk | Not described |
| Incomplete outcome data (attrition bias) | Unclear risk | Above 5% dropped out and the trial did not report how it handled missing data. |
| Selective reporting (reporting bias) | Low risk | The trial was registered before the trial began but the expected outcomes was not reported. The trial reported pain and adverse events. |
| Other bias | Low risk | The trial appeared free of other bias |

***Corey-bloom 2012***

| **Methods** | Randomised, placebo-controlled, crossover trial |
| --- | --- |
| **Participants** | **Inclusion critieria**  Adult patients with multiple sclerosis and spasticity (score ≥ 3 points on the modified Ashworth scale 5 at the elbow, hip or knee).  **Exclusion criteria**  We excluded patients with a history of major psychiatric disorder (other than depression) or substance abuse, substantial neurologic disease other than multiple sclerosis (e.g., epilepsy, head trauma) and severe or unstable medical illnesses, known pulmonary disorders (tuberculosis, asthma), patients who used benzodiazepines to control spasticity or high doses of narcotic medications for pain, and women who were pregnant or breastfeeding. |
| **Interventions** | **Experimental intervention**  (smoked cannabis, once daily for three days. Cigarettes were 800 mg and contained about 4% delta-9-tetrahydrocannabinol (delta-9-THC) by weight.  **Control Intervention**  Identical placebo cigarettes, once daily for three days |
| **Outcomes** | The modified Ashworth scale, daily for pain (using a visual analogue scale), physical performance (using a timed walk) and cognitive function (the PASAT). |
| **Notes** | The authors were contacted on information to judge the missing bias domains (random sequence generation, allocation concealment, blinding of outcome assessment, incomplete outcome data) as well as asked about data for participants at the end of the first wash out period (14 days after randomisation) on email: jcoreybloom@ucsd.edu on 17th of December 2019. |

Risk of bias table

| **Bias** | **Authors' judgement** | **Support for judgement** |
| --- | --- | --- |
| Random sequence generation (selection bias) | Unclear risk | Not described |
| Allocation concealment (selection bias) | Unclear risk | Not described |
| Blinding of participants and personnel (performance bias) | Low risk | Described as double blind with identical placebo |
| Blinding of outcome assessment (detection bias) | Unclear risk | Not described |
| Incomplete outcome data (attrition bias) | Unclear risk | The trial randomised 37 participants but only reported on 30. It appeared to exclude participants with missing data. |
| Selective reporting (reporting bias) | Low risk | The trial reported adverse events and pain |
| Other bias | Low risk | The trial appeared free of other type of bias |

***Cote 2015***

| **Methods** | Randomised, placebo-controlled, parallel group trial. Conducted at Hôtel-Dieu de Québec hospital, department of radio-oncology (Quebec City, QC, Canada), from May 2005 to August 2007 |
| --- | --- |
| **Participants** | **Inclusion criteria**  Histological diagnosis of squamous cell carcinoma of the oral cavity, the oropharynx, the hypopharynx, and/or the larynx; (2) treated by radiotherapy alone, postoperative radiotherapy, radiochemotherapy alone, or postoperative radiochemotherapy; (3) aged 18 to 80 years; (4) no other cancer diagnosis in the past 5 years, except for basal cell and squamous cell carcinoma of the skin; and (5) provided a written informed consent.  **Exclusion criteria**  (1) metastatic disease, (2) history of radiotherapy in the head and neck region, (3) Karnofsky score <60, (4) cognitive impairment, (5) hepatic insufficiency, (6) pregnant or breastfeeding woman, (7) history of hypersensitivity or adverse reactions to marijuana or other cannabinoids, or (8) history of schizophrenia or any other form of psychosis. |
| **Interventions** | **Experimental intervention**  Nabilone as 0.5 mg pills PO.  Nabilone’s administration began the day before the first radiotherapy treatment, with 1 pill at bedtime. The same dose was kept for the entire first week. For the second week, the dose was increased to 2 pills a day. From the third week until the end of radiotherapy treatments, the dose was adjusted by the radio-oncologist to a maximum of 4 pills a day.  **Control intervention**  The corresponding procedure was done in the control group, using the placebo.  **Co intervention**  Concomitant use of anti-emetics(metoclopramide only) and antalgics (only acetaminophen/ codeine, hydromorphone, or transdermal fentanyl) was permitted. |
| **Outcomes** | quality of life scale (items 29-30) of the EORTC QLQ-C30 Secondary endpoints included a 10 cm visual analog scale (VAS) 28 for pain and a count of other antalgic medica- tions used; fluctuation of weight, total days without feeding tube or gastrostomy, and an independent questionnaire to evaluate appetite; an independent questionnaire for nausea and a count of anti-emetic medication used; and an indepen- dent questionnaire to evaluate the toxicity of nabilone. |
| **Notes** | The authors were contacted on information to judge the missing bias domains (random sequence generation, allocation concealment, incomplete outcome data) as well as asked about data for participants for all relevant outcomes by email mathieu.trudel.1@gmail.com on 17th of December 2019. |

Risk of bias table

| **Bias** | **Authors' judgement** | **Support for judgement** |
| --- | --- | --- |
| Random sequence generation (selection bias) | Unclear risk | Not described |
| Allocation concealment (selection bias) | Unclear risk | Not described |
| Blinding of participants and personnel (performance bias) | Low risk | The physicians, nurse, and subjects were blinded. Placebo identical |
| Blinding of outcome assessment (detection bias) | Low risk | The physicians, nurse, and subjects were blinded. Placebo identical. |
| Incomplete outcome data (attrition bias) | Unclear risk | The trial describes multiple dropouts, but it is unclear how it handled missing data |
| Selective reporting (reporting bias) | Unclear risk | The trial reported that "There was no modification of study endpoints during the trial." However, no prepublished protocol was available and the trial only reported adverse effects, not adverse events. |
| Other bias | Low risk | The trial appeared free of other types of bias |

***Devries 2016***

| **Methods** | Randomised, placebo controlled, cross-over study. The study took place at the Radboud University Medical Centre, The Netherlands, from October 2011 to May 2013. |
| --- | --- |
| **Participants** | **Inclusion criteria**  Eligible patients were adults (age > 18 years) diagnosed with CP according to the Marseille and Cambridge Classification System. All patients had chronic abdominal pain, persistent or intermittent on a daily basis during the past 3 months, and considered their pain as severe enough for medical treatment (numeric rating scale (NRS) ≥ 3).  **Exclusion criteria**  Key exclusion criteria were cannabis use in previous year, history of hypersensitivity to THC, BMI <18.0 or >31.2 kg m2, serious painful conditions other than CP, significant medical disorder or concomitant medicationthat may interfere with the study or may pose a risk for the patient, major psychiatric illness in history, epileptic seizure in history, diabetic neuropathy, significant exacerbation in illness within 2 weeks, more than 1 daily defined dose (DDD) benzodiazepines 6 h prior to or following intake of study medication in the opioid subgroup or more than 1 DDD benzodiazepines according to prescription in the non-opioid subgroup (1 DDD was defined as 20 mg oxazepam), positive urine drug screen or alcohol test at screening or on study days, clinically relevant abnormalities in ECG or laboratory results, pregnantor breastfeeding females, intending to conceive achild or participation in another investigational drug study within 90 days before study entry |
| **Interventions** | **Experimental intervention**  Δ9-THC (Namisol® 8 mg simultaneously with placebo diazepam)  **Control intervention**  single dose diazepam (placebo Namisol® simultaneously with diazepam (5 mg non-opioid group/10 mg opioid group)).  **Co intervention**  "Patients used their regular medication, including painkillers, according to prescription on both study days." |
| **Outcomes** | Pain (VAS), adverse events, pharmokinitec and dynamic measurements. |
| **Notes** | NCT01318369. The author was contacted by email: marjan.devries@radboudumc.nl on 18.12.2019 regarding missing outcomes from clinicaltrials.gov, data on outcomes at the end of the wash out period. |

Risk of bias table

| **Bias** | **Authors' judgement** | **Support for judgement** |
| --- | --- | --- |
| Random sequence generation (selection bias) | Low risk | "Computergenerated list of random numbers" |
| Allocation concealment (selection bias) | Unclear risk | Not described |
| Blinding of participants and personnel (performance bias) | Low risk | "Patients, staff and investigators were all blinded by a double dummy design" |
| Blinding of outcome assessment (detection bias) | Low risk | "Patients, staff and investigators were all blinded by a double dummy design" |
| Incomplete outcome data (attrition bias) | Unclear risk | 3 participants out of 25 randomised seemed to be missing as part of the final analyses, but it was unclear how the missing data was handled. |
| Selective reporting (reporting bias) | High risk | The trial did not report on the outcomes registered at clinicaltrials.gov |
| Other bias | Low risk | The trial appeared free of other types of bias |

***Devries 2016a***

| **Methods** | Randomised, double-blind, placebo-controlled, parallel design. The study was executed at the Radboud university medical center, the Netherlands from October 2012 to July 2014 |
| --- | --- |
| **Participants** | **Inclusion criteria**  Adult patients (age >18 years) suffering from abdominal pain developed after a surgical procedure or resulting from chronic pancreatitis were eligible for participation, if they had persistent or intermittent abdominal pain (on a daily basis for at least 3 months) severe enough for medical treatment (average NRS ≥ 3).  **Exclusion criteria**  Key exclusion criteria were: daily cannabis use in past three years; history of hypersensitivity to THC; serious painful conditions other than PSP or CP; significant medical disorder or concomitant medication that may interfere with the study or may pose a risk for the patient; major psychiatric illness in history; epileptic seizure in history; affected sensory input such as diabetic neuropathy; BMI >36.0 kg/m2; significant exacerbation in illness within two weeks; positive urine drug screen or alcohol test at screening or on study days; clinically relevant abnormalities in ECG or laboratory results; pregnant or breastfeeding females; intending to conceive a child; or participation in another investigational drug study within 90 days before study entry. |
| **Interventions** | **Experimental intervention**  Tablets with standardized Δ9-THC content (Namisol®, Echo Pharmaceuticals, Weesp, the Netherlands). Subjects in the THC group were given the tablet first in a step-up phase (3 mg, 3 times daily for 5 days and then 5 mg, 3 times daily for 5 days) followed by a stable dose phase (8 mg, 3 times daily until day 50–52). |
| **Outcomes** | Pain on VAS, quality of life, adverse events, pharmakokinetics. |
| **Notes** | Clinicaltrials: NCT01562483 and NCT01551511. The trial author was contacted by email: marjan.devries@radboudumc.nl on 18.12.2019 about complete data for all participants on serious adverse events, adverse events, quality of life, pain as well as enquire about the missing outcomes stated on clinicaltrials.gov. |

Risk of bias table

| **Bias** | **Authors' judgement** | **Support for judgement** |
| --- | --- | --- |
| Random sequence generation (selection bias) | Low risk | "Independent pharmacists dispensed either active or placebo tablets according to a computer-generated randomization list stratified for opioid and non-opioid users using separate lists." |
| Allocation concealment (selection bias) | Low risk | "Treatment allocation was strictly concealed from participants, investigators, and all other study personnel involved in the study until end of study and database lock." |
| Blinding of participants and personnel (performance bias) | Low risk | "HC tablet or identical matching placebos" |
| Blinding of outcome assessment (detection bias) | Low risk | "Treatment allocation was strictly concealed from participants, investigators, and all other study personnel involved in the study until end of study and database lock." |
| Incomplete outcome data (attrition bias) | Unclear risk | The trial reported that no participants were lost to follow up. However, the pain data was only reported at follow up in a modified ITT analysis including only 50 patients out of 62. |
| Selective reporting (reporting bias) | High risk | The trial did not report on all prespecified outcomes on clinicaltrials.gov (missing data on EEG). |
| Other bias | Low risk | The trial appeared free of other types of bias |

***Eibach 2020***

| **Methods** | A Randomised, double-blinded, placebo-controlled Clinical Trial, Crossover assignment |
| --- | --- |
| **Participants** | Inclusion criteria:   1. Subjects were screened for age (18–65 years), 2. Vital signs, and pain intensity (≥4 on an 11-point numeric rating scale (NRS)). 3. The diagnosis of HIV-associated sensory neuropathy was confirmed by a clinician based on patient history, 4. The Douleur Neuropathique 4 inter-view (DN4i), 5. the Clinical HIV-associated Neuropathy Too   Exclusion criteria:   1. Pregnancy and lactation 2. Major psychiatric con-ditions, 3. Severe diseases of the central nervous system, 4. Hepatic, renal, or cardiovascular diseases, 5. Use of conventional cannabinoids (CBs) 6. Examined by blood test. |
| **Interventions** | Each patient was monitored for 13 weeks. After the screening phase, baseline values on pain scales, questionnaires, and medications were recorded during a 1-week phase. This was followed by 4-week treatment phase A with either placebo or CBDV. A subsequent 3-week washout phase was included to eliminate potential carry-over effects.  Thereafter, another 1-week baseline phase ensued, followed by treatment phase B. Patients were then followed up for another 3 weeks. |
| **Outcomes** | The primary outcome:  Pain intensity measured thrice a day by an 11-point NRS  The number of responders and nonresponders to each treatment was determined  Secondary outcome:   1. Pain characteristics, 2. Quality of life, 3. Quality of sleep   They used painDETECT, the Brief Pain Inventory, and the DN4i for evaluation of pain intensity and pain characteristics,  The Hospital Anxiety and Depression Scale to evaluate anxiety and depression,  And the 36-Item Short Form Survey, the Patient Global Impression of Change, and the Insomnia Severity Index for quality of life and sleep, |
| **Notes** |  |

Risk of bias table

| **Bias** | **Authors' judgement** | **Support for judgement** |
| --- | --- | --- |
| Random sequence generation (selection bias) | Low risk | Randomization to the sequence of treatments occurred in blocks of four by use of paper-based, computer-generated random lists, which were stored in a locked cabinet |
| Allocation concealment (selection bias) | Low risk | Allocation to the treatment sequence was documented and kept in sealed envelopes |
| Blinding of participants and personnel (performance bias) | Low risk | All patients and staff involved in patient contacts and assessment of outcomes were blinded until the end of the study |
| Blinding of outcome assessment (detection bias) | Low risk | All patients and staff involved in patient contacts and assessment of outcomes were blinded until the end of the study |
| Incomplete outcome data (attrition bias) | Low risk | Statistical analysis was based on the intention-to-treat principle (i.e., every patient who started treatment and had at least one post-baseline measurement of the primary end point was included in the full set for the efficacy analysis |
| Selective reporting (reporting bias) | Unclear risk | The study was registered at EudraCT (https://www.clinicaltrialsregister.eu/) under number 2014-005344-17. |
| Other bias | Low risk | EU-consortium: “NeuroPain—Neuropathic pain: biomarkers and druggable targets within the endogenous analgesia system” (EU FP7-HEALTH-2013-INNOVATION-1; No. 602891-2). C.S. was also supported by ‘Charite 3R - Replace Reduce Refine’ and by Deutsche Forschungsgemeinschaft (STE 477/19-1) |

***Ellis 2008***

| **Methods** | Randomised, phase II, single group, double-blind, placebo-controlled, crossover trial. |
| --- | --- |
| **Participants** | **Inclusion criteria**  Study participants were adults with documented HIV infection, neuropathic pain refractory to a least two previous analgesics, and an average score of 5 or higher on the pain intensity subscale of the Descriptor Differential Scale (DDS).  **Exclusion criteria**  Exclusion criteria were (1) current DSM-IV substance use disorders; (2) lifetime history of dependence on cannabis; (3) previous psychosis with or intolerance to cannabinoids; (4) concurrent use of approved cannabinoid medications (ie Marinol); (5) positive urine toxicology screen for cannabinoids during the wash-in week before initiating study treatment; and (6) serious medical conditions that might affect participant safety or the conduct of the trial. Individuals with a previous history of alcohol or other drug dependence were eligible provided that criteria for dependence had not been met within the last 12 months. Subjects were excluded if urine toxicology demonstrated ongoing use of nonprescribed, recreational drugs such as methamphetamine and cocaine. |
| **Interventions** | **Experimental intervention**  "Over four smoking sessions, each participant titrated to the dose (‘target dose’) affording the best achievable pain relief without unacceptable adverse effects. Titration was started at 4% THC or placebo and adjusted incrementally downwards (to 2 or 1%) if side effects were intolerable, or upwards (to 6 or 8%) if pain relief was incomplete." Maximum procentage was 8%.  **Control intervention**  Placebo  In the same manner as the group receiving THC, the placebo group "Over four smoking sessions, each participant titrated to the dose (‘target dose’) affording the best achievable pain relief without unacceptable adverse effects." Titration was startedand adjusted incrementally up or downwards. |
| **Outcomes** | Multiple pain-associated constructs (VAS), including analgesia, improvement in function, and relief of pain-associated emotional distress |
| **Notes** | The author was contacted by email: roellis@ucsd.edu on 19.12.2019 for data before participants crossed over and clarification of the trial had a prepublished protocol. |

Risk of bias table

| **Bias** | **Authors' judgement** | **Support for judgement** |
| --- | --- | --- |
| Random sequence generation (selection bias) | Low risk | "Randomization was performed by a research pharmacist using a random number generator, and the key to study assignment was withheld from investigators until completion statistical analyses." |
| Allocation concealment (selection bias) | Low risk | "Randomization was performed by a research pharmacist using a random number generator, and the key to study assignment was withheld from investigators until completion statistical analyses." |
| Blinding of participants and personnel (performance bias) | Low risk | "Placebo cigarettes were made from whole plant material with cannabinoids removed and were identical in appearance to active cigarettes." |
| Blinding of outcome assessment (detection bias) | Low risk | "Randomization was performed by a research pharmacist using a random number generator, and the key to study assignment was withheld from investigators until completion statistical analyses." |
| Incomplete outcome data (attrition bias) | Low risk | "A conservative ITT analysis was also performed, using multiple imputation (MI) for the six subjects with incomplete data."  "Secondary analyses were performed for study completers, except for the adverse event (AE) analysis, which included all Randomised subjects." |
| Selective reporting (reporting bias) | Unclear risk | The trial did not appear to have a prepublished protocol and the trial did not report adverse events comprehensively |
| Other bias | Low risk | The trial appeared free of other types of bias |

***Fallon 2017a***

| **Methods** | Phase 3, double-blind, Randomised, placebo-controlled trial. 101 centres in Belgium, Bulgaria, Czech Republic, Estonia, Germany, Hungary, Latvia, Lithuania, Poland, Romania, the United Kingdom and the United States |
| --- | --- |
| **Participants** | **Inclusion criteria**  Eligible patients enrolled in the study had an advanced incurable stage of cancer, were ≥18 years of age, and had a clinical diagnosis of cancer-related pain that was unalleviated by an optimized maintenance dose of Step 3 opioid therapy. Opioid therapy was considered optimized if (1) a dose increase was clinically inappropriate due to opioid-related side effects or (2) further efficacy benefit was not expected at higher doses (for the second definition, patients had to be receiving ≥90 mg morphine equivalents/day, inclusive of maintenance and breakthrough opioids). The maintenance opioid was preferably a sustained-release formulation, but an around-the-clock immediate-release formulation was acceptable. To be eligible, patients also had to fulfil the following criteria on each of three consecutive days during the screening period: ≤4 opioid breakthrough analgesic episodes per day (averaged over the 3 days), a stable maintenance opioid therapy dose, average pain ≥ 4 and ≤8 on a 0–10 NRS and average pain scores on the NRS that did not change by more than 2 points from the beginning to end of screening (i.e. no more than a 2-point difference between the highest and lowest scores, with all scores remaining between 4 and 8).  **Exclusion criteria**  Key exclusion criteria in study 1 included baseline use of morphine at >500 mg morphine equivalents/day (inclusive of maintenance and breakthrough opioids), current use of more than one type of breakthrough opioid analgesic, planned clinical interventions that would affect pain, and any history of schizophrenia or substance abuse including recreational use of cannabis product. |
| **Interventions** | **Experimental intervention**  Sativex oral mucosal spray (THC (27 mg/mL): CBD (25 mg/mL))  **Control Intervention**  Matching placebo  **Common for both interventions**  Treatment was initiated as a single spray in the evening of the first day of treatment and was gradually increased by one additional spray per day (15 minutes apart) according to a pre-specified dose escalation protocol (Supplementary Table 1) until patients experienced unacceptable side effects, received acceptable pain relief, or reached the maximum allowed daily dosage of 10 sprays per day (patients were advised to reach at least 3 sprays per day). |
| **Outcomes** | Primary: Percent improvement from baseline to the end of treatment in average pain NRS score.  Secondary: Mean change from baseline to the end of treatment in average pain NRS score, worst pain NRS score, sleep disruption NRS and maintenance, breakthrough, and total opioid use per day in morphine equivalents, Subject Global Impression of Change (SGIC), Patient Satisfaction Questionnaire (PSQ), Physician Global Impression of Change (PGIC) and a constipation NRS. |
| **Notes** | Two trials were reported in one publication. The results of study 1 are reported as Fallon 2017 (NCT01361607). |

Risk of bias table

| **Bias** | **Authors' judgement** | **Support for judgement** |
| --- | --- | --- |
| Random sequence generation (selection bias) | Unclear risk | Not described |
| Allocation concealment (selection bias) | Unclear risk | Not described |
| Blinding of participants and personnel (performance bias) | Low risk | Described as triple blinded and with matching placebo. |
| Blinding of outcome assessment (detection bias) | Low risk | Described as triple blinded and with matching placebo. |
| Incomplete outcome data (attrition bias) | Unclear risk | "All patients who were Randomised, received at least one dose of study medication, and had at least one post-Randomised efficacy endpoint comprised the intention-to-treat (ITT) analysis set." It was however, unclear how the trial handled missing data for the final analysis, i.e. did the trial use imputation or did it use last observation carried forward. There is a brief discription of some sort of imputation on clinicaltrials.gov, but not enough for us to properly understand what the trial authors did. |
| Selective reporting (reporting bias) | High risk | Many secondary outcomes on clinicaltrials.gov were added after the trial begun. |
| Other bias | Low risk | The trial appeared free of other types of bias |

***Fallon 2017b***

| **Methods** | Study 2 was conducted in 65 centres in Australia, Bulgaria, Germany, Hungary, India, Israel, Italy, Lithuania, Poland, Romania, Spain, Taiwan and the United Kingdom |
| --- | --- |
| **Participants** | **Inclusion criteria (identical to study 1)**  Eligible patients enrolled in the study had an advanced incurable stage of cancer, were ≥18 years of age, and had a clinical diagnosis of cancer-related pain that was unalleviated by an optimized maintenance dose of Step 3 opioid therapy. Opioid therapy was considered optimized if (1) a dose increase was clinically inappropriate due to opioid-related side effects or (2) further efficacy benefit was not expected at higher doses (for the second definition, patients had to be receiving ≥90 mg morphine equivalents/day, inclusive of maintenance and breakthrough opioids). The maintenance opioid was preferably a sustained-release formulation, but an around-the-clock immediate-release formulation was acceptable. To be eligible, patients also had to fulfil the following criteria on each of three consecutive days during the screening period: ≤4 opioid breakthrough analgesic episodes per day (averaged over the 3 days), a stable maintenance opioid therapy dose, average pain ≥ 4 and ≤8 on a 0–10 NRS and average pain scores on the NRS that did not change by more than 2 points from the beginning to end of screening (i.e. no more than a 2-point difference between the highest and lowest scores, with all scores remaining between 4 and 8).  In addition participants had to demonstrate an improvement of 15% or more on pain NRS score in the first part of study 2.  **Exclusion criteria**  Key exclusion criteria in study 1 included baseline use of morphine at >500 mg morphine equivalents/day (inclusive of maintenance and breakthrough opioids), current use of more than one type of breakthrough opioid analgesic, planned clinical interventions that would affect pain, and any history of schizophrenia or substance abuse including recreational use of cannabis product. |
| **Interventions** | **Experimental intervention**  Participants received their self-titrated doses of Sativex oral mucosal spray (THC (27 mg/mL): CBD (25 mg/mL)).  **Control Intervention**  Matching placebo  **Co-intervention**  In the first part of the study, all patients underwent Sativex titration during a single-blind treatment period lasting 10 days followed by 4 days of therapy at the titrated dose |
| **Outcomes** | The primary endpoint was the mean change from the randomization baseline to the end of treatment in average pain NRS score  Secondary: Mean change from baseline to the end of treatment in average pain NRS score, worst pain NRS score, sleep disruption NRS and maintenance, breakthrough, and total opioid use per day in morphine equivalents, Subject Global Impression of Change (SGIC), Patient Satisfaction Questionnaire (PSQ), Physician Global Impression of Change (PGIC) and a constipation NRS. |
| **Notes** | The results of study 2 are reported as Fallon 2017a (NCT01424566) |

Risk of bias table

| **Bias** | **Authors' judgement** | **Support for judgement** |
| --- | --- | --- |
| Random sequence generation (selection bias) | Unclear risk | Not described |
| Allocation concealment (selection bias) | Unclear risk | Not described |
| Blinding of participants and personnel (performance bias) | Low risk | Described as triple blinded and with matching placebo. |
| Blinding of outcome assessment (detection bias) | Low risk | Described as triple blinded and with matching placebo. |
| Incomplete outcome data (attrition bias) | Unclear risk | "All patients who were Randomised, received at least one dose of study medication, and had at least one post-Randomised efficacy endpoint comprised the intention-to-treat (ITT) analysis set." It was however, unclear how the trial handled missing data for the final analysis, i.e. did the trial use imputation or did it use last observation carried forward. |
| Selective reporting (reporting bias) | High risk | Many secondary outcomes on clinicaltrials.gov were added after the trial begun. |
| Other bias | Low risk | The trial appeared free of other types of bias |

***Hunter 2018***

| **Methods** | A Phase 2A, Randomised, double-blind, placebo-controlled, multiple-dose study assessed ZYN002 administered twice daily for 12 weeks to adults with knee pain due to OA |
| --- | --- |
| **Participants** | Patients met ACR criteria for osteoarthritis of the knee and underwent a 1-week washout to stop current anti-inflammatory agents/other analgesics (except paracetamol) followed by a 7 to 10-day baseline period capturing daily worst pain ratings using a 0 to 10 numeric rating scale. |
| **Interventions** | Experimental: ZYN002 250mg  Experimental: ZYN002 500mg  Control: placebo |
| **Outcomes** | Primary outcome: change from baseline in the weekly mean of the 24-hour average worst pain score at week 12  Secondary outcome: responder analysis defined as average weekly improvement in worst pain score of 30% or more |
| **Notes** | Sponsered by Zynerba Pharmaceuticals |

Risk of bias table

| **Bias** | **Authors' judgement** | **Support for judgement** |
| --- | --- | --- |
| Random sequence generation (selection bias) | Unclear risk | Quote: "randomised"  Judgement: probably done, but no mention of random sequence generation |
| Allocation concealment (selection bias) | Unclear risk | Not mentioned. |
| Blinding of participants and personnel (performance bias) | Unclear risk | Quote: "double-blind" |
| Blinding of outcome assessment (detection bias) | Unclear risk | Not mentioned. |
| Incomplete outcome data (attrition bias) | Unclear risk | Not mentioned. |
| Selective reporting (reporting bias) | Unclear risk | No protocol obtained. No reporting of serious adverse events. |
| Other bias | High risk | Zynerba Pharmaceuticals |

***Issa 2014***

| **Methods** | Multidose, randomised, double-blind, placebo-controlled, crossover trial. |
| --- | --- |
| **Participants** | Thirty chronic non-cancer pain participants on opioid therapy were recruited through Brigham and Women’s Hospital. The ages of participants ranged from 21 to 67 years (median age, 43.5 ± 11.8), 53% were female, and the majority selfidentified as white (96.7%). Most participants (66.7%) reported chronic pain >5 years, and low back pain (66.7%) was the most prevalent. The mean morphine equivalent dose per day was 68.1 mg (range, 7.5 to 228.0 mg). Baseline pain intensity was 6.9/10 (± 1.3). Nineteen participants (63.3%) reported using marijuana in the past, 8 of whom reported using within the past year. All participants were required to abstain from use for 1 month before and during enrollment. Participants were also required to have stable opioid analgesic dose for >6 months and report pain of at least 4 on a 0 to 10 numeric rating scale. They were asked to reschedule their visit if their average pain over the past 24 hours was <4/10, assessed on the morning of the anticipated treatment.  Exclusion: pain associated with cancer; opioid dosing more frequently than every 8 hours; use of transdermal patch or intrathecal opioids (as it would be difficult to titrate their opioid dose during the trial); significant depression and/or anxiety symptoms (scores >11 on the Hospital Anxiety and Depression Scale [HADS]); current substance abuse by selfreport and psychosocial history; and any involvement in active litigation, compensation, or disability issues. |
| **Interventions** | Experimental: dronabinol 10mg  Experimental: dronabinol 20mg  Control: placebo  Study drugs were given over three study sessions, where the participants were crossed over to one of the other groups until having received dronabinol 10mg, dronabinol 20mg, and placebo. The study sessions were separated by a minimum of three days. |
| **Outcomes** | Primary outcome: Addiction Research Center Inventory (ARCI). |
| **Notes** |  |

Risk of bias table

| **Bias** | **Authors' judgement** | **Support for judgement** |
| --- | --- | --- |
| Random sequence generation (selection bias) | Low risk | Quote: "Randomisation scheme generated by the Investigational Drug Service hospital pharmacy"  Judgement: probably done |
| Allocation concealment (selection bias) | Unclear risk | Not mentioned |
| Blinding of participants and personnel (performance bias) | Low risk | Quote: "Both personnel and participants were blinded" and "each participant received identically appearing placebo"  Judgement: probably done |
| Blinding of outcome assessment (detection bias) | Unclear risk | Not mentioned. |
| Incomplete outcome data (attrition bias) | Low risk | One participant did not complete the trial. It was unclear if there were complete data for all participants. |
| Selective reporting (reporting bias) | Unclear risk | No protocol obtained. No reporting of pain on NRS or VAS and no reporting of serious adverse events. |
| Other bias | High risk | Sponsored partly by a grant from Solvay Pharmaceuticals Inc. |

***Jain 1981***

| **Methods** | Randomised, double-blind, multiple parallel groups. |
| --- | --- |
| **Participants** | Patients with moderate to severe acute postoperative fracture or trauma pain requiring parenteral analgesic.  Exclusion criteria: nursing or pregnant women; head injury; heart, live, gastrointestinal, renal, or hematological disease; history of drug abuse or addiction; patients receiving analgesic, anti-inflammatory, tranqulizing, sedative, or anaesthetic medicine. |
| **Interventions** | Experimental: single intramuscular injection of levonantradol 1.5, 2, 2.5, or 3 mg  Control: intramuscular placebo |
| **Outcomes** | - Pain intensity (0 = none, 1 = slight, 2 = some, 3 = a lot, 4 = complete). - Pain analog (patient's self-rating of pain) on a 0 to 25 scale. - Side effects. |
| **Notes** | Sponsored by Pfizer Inc. The trial reports "side effects" not adverse events, but we have used the data anyways |

Risk of bias table

| **Bias** | **Authors' judgement** | **Support for judgement** |
| --- | --- | --- |
| Random sequence generation (selection bias) | Unclear risk | Quote: "randomised" Judgement: probably done, but no mention of random sequence generation |
| Allocation concealment (selection bias) | Unclear risk | Not mentioned |
| Blinding of participants and personnel (performance bias) | Low risk | Quote: "double-blind Judgement: probably done |
| Blinding of outcome assessment (detection bias) | Unclear risk | Not mentioned |
| Incomplete outcome data (attrition bias) | Unclear risk | "Subjects who discontinued the study due to inadequate pain relief were assigned a pain intensity score equal to the score at the time they dropped out and a pain relief score equal to zero for the remainder of the study. "  "Only four patients on placebo and one patient on the 3.0-mg dose received a supplemental analgesic."  This could indicate that 5 participants had imputed data in the above manner, which seems to be an inadequate way of handling missing data. |
| Selective reporting (reporting bias) | Unclear risk | No protocol obtained. No reporting of pain on NRS or VAS and no reporting of serious adverse events |
| Other bias | High risk | Sponsored by Pfizer Inc. |

***Jochimsen 1978***

| **Methods** | Double-blind, 5-way crossover, placeb-controlled trial. |
| --- | --- |
| **Participants** | The subjects were all suffering from pain related to malignancies and had a history of frequent analgesie use, though none were receiving large doses of narcotics. No patients having conditions interfering with drug metabolism, severe organic disease other than cancer, pregnancy, or major psychiatric disorders were included in the study. Of the 35 patients completing the study, 29 were women and 6 were men, with an age range of 38 to 77 yr (mean, 57). |
| **Interventions** | Upon selection for study each patient was admitted to the Clinical Research Center at the University of  lowa and randomly assigned to a sequence of treatment with the 5 test preparations. Each patient received single doses of 2 and 4 mg of BPP, 60 and 120 mg of codeine sulfate, and placebo by mouth. The test preparations, in identical-appearing capsules, were given at least 1 hr after breakfast and at least 4 hr after the last known analgesic, and only when the patient experienced moderate or severe pain. If relief was not apparent within 4 hr, a known analgesic was administered at the patient's request and observations for that day discontinued. |
| **Outcomes** | Nurse recorded pain relief every 6hrs: 0, none; 1, slight; 2, moderate; 3, a lot; 4, complete.  Patient self-rated pain on a VAS. |
| **Notes** | Funding by Abbott Laboratories. |

Risk of bias table

| **Bias** | **Authors' judgement** | **Support for judgement** |
| --- | --- | --- |
| Random sequence generation (selection bias) | Unclear risk | Quote: "randomised"  Judgement: probably done, but no mention of randomisation sequence generation. |
| Allocation concealment (selection bias) | Unclear risk | Not mentioned. |
| Blinding of participants and personnel (performance bias) | Low risk | Quote: "double-blind" and "test-capsules identical appearing"  Judgement: probably done |
| Blinding of outcome assessment (detection bias) | Unclear risk | Not mentioned. |
| Incomplete outcome data (attrition bias) | Unclear risk | 2 patients did not complete the study, reasons not provided. |
| Selective reporting (reporting bias) | Unclear risk | No protocol obtained. No reporting of pain on VAS or NRS in a usable way. No reporting of serious adverse events. |
| Other bias | High risk | Funding by Abbott Laboratories. |

***Johnson 2010 (THC)***

| **Methods** | Two-week (two-day baseline and twoweek treatment), multicenter, double-blind, Randomised, placebo-controlled, parallel group study. According to clinicaltrials.gov there trial was conducted from 2002 to 2004. |
| --- | --- |
| **Participants** | Eligible patients recorded a pain severity score of 4 or above on a 0 to 10 Numerical Rating Scale (NRS) on both days of the two-day baseline period.  Patients were excluded if they had cancers affecting the oral cavity; radiotherapy to the floor of the mouth; major psychiatric or cardiovascular disorders; epilepsy; renal or hepatic impairment; or if they were pregnant, lactating, or not using adequate contraception. Patients who had received therapies expected to confound the study outcome (epidural analgesia within 48 hours of screening; palliative radio-, chemo-, or hormonal therapy within two weeks of screening; or CBs within seven days of randomization) were also excluded. Patients taking levodopa, sildenafil, or fentanyl or patients with a hypersensitivity to CBs were excluded on safety grounds. |
| **Interventions** | Experimental: THC spray delivering 2.7mg THC.  Control: placebo delivering excipients and colorants. |
| **Outcomes** | Primary outcomes:   - change from baseline in NRS pain score - use of breakthrough analgesia   Secondary outcomes:   - opioid background medication - patient assessment of sleep quality, nausea, memory, concentration, and appetite. - Brief Pain Inventory-Short form (BPI-SF) - European Organisation for Research and Treatment of Cancer (EORTC) Quality of Life Questionnaire (QLQ-C30). - Adverse events |
| **Notes** | This trials did provide data that was compatible with at meta-analysis in regards to the outcome Quality of Life based on (EORTC) Quality of Life Questionnaire (QLQ-C30). |

Risk of bias table

| **Bias** | **Authors' judgement** | **Support for judgement** |
| --- | --- | --- |
| Random sequence generation (selection bias) | Unclear risk | Quote: "randomised" Judgment: probably done, but no mention of random sequence generation |
| Allocation concealment (selection bias) | Unclear risk | Not mentioned |
| Blinding of participants and personnel (performance bias) | Low risk | Quote: "double-blind" and there was added colorants to mask the placebo. Judgement: probably done |
| Blinding of outcome assessment (detection bias) | Low risk | Quote: "Masking: Quadruple (participant, care provider, investigator, outcomes assessor) Judgement: probably done |
| Incomplete outcome data (attrition bias) | Unclear risk | Even loss to follow-up. Intention to treat data was used but it was unclear how the trial handled missing data (we do not know if the trial used last observation carried forward or mulitiple imputations). |
| Selective reporting (reporting bias) | Low risk | The protocol was registered after the trial began. The primary outcomes were changed after registration at clinicaltrials.gov. The trial reported serious adverse events and reported pain. <https://clinicaltrials.gov/ct2/show/NCT00674609> |
| Other bias | Unclear risk | Sponsored by GW Pharmaceuticals. |

***Johnson 2010 (THC/CBD)***

| **Methods** | Two-week (two-day baseline and twoweek treatment), multicenter, double-blind, Randomised, placebo-controlled, parallelgroup study. According to clinicaltrials.gov there trial was conducted from 2002 to 2004. |
| --- | --- |
| **Participants** | Eligible patients recorded a pain severity score of 4 or above on a 0 to 10 Numerical Rating Scale (NRS) on both days of the two-day baseline period.  Patients were excluded if they had cancers affecting the oral cavity; radiotherapy to the floor of the mouth; major psychiatric or cardiovascular disorders; epilepsy; renal or hepatic impairment; or if they were pregnant, lactating, or not using adequate contraception. Patients who had received therapies expected to confound the study outcome (epidural analgesia within 48 hours of screening; palliative radio-, chemo-, or hormonal therapy within two weeks of screening; or CBs within seven days of randomization) were also excluded. Patients taking levodopa, sildenafil, or fentanyl or patients with a hypersensitivity to CBs were excluded on safety grounds. |
| **Interventions** | Experimental: THC:CBD spray delivering 2.7mg THC and 2.5mg CBD.  Control: placebo delivering excipients and colorants. |
| **Outcomes** | Primary outcomes:   - change from baseline in NRS pain score - use of breakthrough analgesia   Secondary outcomes:   - opioid background medication - patient assessment of sleep quality, nausea, memory, concentration, and appetite. - Brief Pain Inventory-Short form (BPI-SF) - European Organisation for Research and Treatment of Cancer (EORTC) Quality of Life Questionnaire (QLQ-C30). - Adverse events |
| **Notes** | We mainly used the trial data from <https://clinicaltrials.gov/ct2/show/NCT00674609>  This trials did provide data that was compatible with at meta-analysis in regards to the outcome Quality of Life based on (EORTC) Quality of Life Questionnaire (QLQ-C30). |

Risk of bias table

| **Bias** | **Authors' judgement** | **Support for judgement** |
| --- | --- | --- |
| Random sequence generation (selection bias) | Unclear risk | Quote: "randomised" Judgment: probably done, but no mention of random sequence generation |
| Allocation concealment (selection bias) | Unclear risk | Not mentioned |
| Blinding of participants and personnel (performance bias) | Low risk | Quote: "double-blind" and there was added colorants to mask the placebo. Judgement: probably done |
| Blinding of outcome assessment (detection bias) | Low risk | Quote: "Masking: Quadruple (participant, care provider, investigator, outcomes assessor) Judgement: probably done |
| Incomplete outcome data (attrition bias) | Unclear risk | Even loss to follow-up. Intention to treat data was used but it was unclear how the trial handled missing data (we do not know if the trial used last observation carried forward or mulitiple imputations). |
| Selective reporting (reporting bias) | Low risk | The protocol was registered after the trial began. The primary outcomes were changed after registration at clinicaltrials.gov. The trial reported serious adverse events and reported pain. <https://clinicaltrials.gov/ct2/show/NCT00674609> |
| Other bias | Unclear risk | Sponsored by GW Pharmaceuticals. |

***Kalliomäki 2013***

| **Methods** | Randomised, double-blind, double-dummy, placebo-controlled trial. |
| --- | --- |
| **Participants** | Healthy male or non-fertile females aged 18-45 years, with a BMI between 18 and 33 and bodyweight between 50 and 120kg, scheduled for partially or completely impacted mandibular third molar surgery with bone removal. |
| **Interventions** | Experimental: AZD1940 (CB1/CB2 receptor agonist) 800 microgram + naproxen placebo (n=60)  Control: AZD1940 placebo + naproxen placebo (n=60)  Study treatment was administered 1.5hrs before surgery. Patients could ask for 1000mg acetaminophen as rescue medication. |
| **Outcomes** | Primary outcome: post-operative pain on visual analogue scale (0-100mm) from completion of surgery until 8hrs thereafter. Measurements were carried out every 20min for 4h and every 60min thereafter. The VAS-area under the curve was the primary outcome.  Secondary outcomes: pain on jaw movement, time from end of surgery to rescue pain medication, and proportion of participants taking rescue pain medication.  Subjective CNS-related effects were also assessed at a visual analogue mood scale: "stimulated", "high", "anxious", "sedated", and "down". |
| **Notes** | We did not use data from participants receiving naproxen only. |

Risk of bias table

| **Bias** | **Authors' judgement** | **Support for judgement** |
| --- | --- | --- |
| Random sequence generation (selection bias) | Unclear risk | Quote: "randomised"  Judgement: probably done, but no mention of random sequence generation |
| Allocation concealment (selection bias) | Unclear risk | Not mentioned. |
| Blinding of participants and personnel (performance bias) | Low risk | Quoto: "double-blind"  Judgement: probably done |
| Blinding of outcome assessment (detection bias) | Unclear risk | Not mentioned. |
| Incomplete outcome data (attrition bias) | Low risk | No loss to follow-up |
| Selective reporting (reporting bias) | Unclear risk | No protocol obtained. Serious adverse events reported. Pain only reported as area under the curve. |
| Other bias | High risk | Funded by AstraZeneca. First author employee of AstraZeneca. |

***Kantor 1981***

| **Methods** | Randomised, double-blind, placebo-controlled |
| --- | --- |
| **Participants** | Participants with post-operative pain. |
| **Interventions** | Experimental: levonantradol intramuscularly 0.25mg, 0.5mg, or 1mgm  Control: placebo |
| **Outcomes** | SPID score. |
| **Notes** |  |

Risk of bias table

| **Bias** | **Authors' judgement** | **Support for judgement** |
| --- | --- | --- |
| Random sequence generation (selection bias) | Unclear risk | Quote: "randomised"  Judgement: probably done, but no mention of random sequence generation. |
| Allocation concealment (selection bias) | Unclear risk | Not mentioned |
| Blinding of participants and personnel (performance bias) | Unclear risk | Quote: "double-blind"  Judgement: probably done, but no description of how. |
| Blinding of outcome assessment (detection bias) | Unclear risk | Not mentioned |
| Incomplete outcome data (attrition bias) | Unclear risk | Not mentioned |
| Selective reporting (reporting bias) | Unclear risk | No protocol. No reporting of pain on VAS or NRS, no reporting of serious adverse events. |
| Other bias | Unclear risk | No mention of conflicts of interest or funding. |

***Karst 2003***

| **Methods** | Randomised, placebo-controlled, double-blind crossover trial |
| --- | --- |
| **Participants** | Chronic neuropathic pain with hyperalgesia and allodynia. |
| **Interventions** | Experimental: 7-day treatment with CT-3 2x10mg capsules BID for the first 4 days and 4x10mg capsules BID for the last 3 days.  Control: identical placebo |
| **Outcomes** | Primary outcome: pain diary, patients completed the visual analogue scale and the verbal rating sale at 11 am and 4 pm at each day.  Secondary outcomes: adverse events, Trail-Making TEst, Addiction Research Center Inventory-Marijuana scale. |
| **Notes** |  |

Risk of bias table

| **Bias** | **Authors' judgement** | **Support for judgement** |
| --- | --- | --- |
| Random sequence generation (selection bias) | Low risk | The trial was described as randomised using a computer sequence. |
| Allocation concealment (selection bias) | Low risk | The sequence was translated to packaged bottles labeled 1-21. In sequential order, the patients were given corresponding packed bottles. The bottles had been prepacked by a external pharmacist. |
| Blinding of participants and personnel (performance bias) | Low risk | Identical looking bottled. Quote: "double-blind"  Judgement: probably done, but not described how. |
| Blinding of outcome assessment (detection bias) | Low risk | Assessments were done by medical students unaware of the assignment. |
| Incomplete outcome data (attrition bias) | Unclear risk | 1 person in each group was lost to follow-up. No data were imputed for these participants |
| Selective reporting (reporting bias) | Low risk | No protocol obtained. The trial assessed both VAS and serious adverse events (the trial reported no major physical adverse events) |
| Other bias | High risk | Funded by Atlantic Technology Ventures Inc and Indevus Pharmaceuticals Inc. One of the investigators was a patent holder of the technology used in the trial. |

***Killestein 2002***

| **Methods** | Randomised, double-blind, placebo-controlled, twofold crossover study. |
| --- | --- |
| **Participants** | We studied 16 patients with progressive MS (10 with secondary progressive [SP] and six with primary progressive [PP] MS). Patients were eligible for study if they had disease duration 1 year, severe spasticity (mean Ashworth spasticity score 6 of 2 or more in at least one limb) during screening, and Expanded Disability Status Scale (EDSS) score between 4 and 7.5  Exclusion: other disease of clinical importance, use of other investigational drugs, disease exacerbation, steroid treatment, or use of cannabinoids in the 2 months preceding study entry, history of alcohol or drug abuse, depression, psychosis, scizophrenia. |
| **Interventions** | Experimental: dronabinol 2.5 mg BID the first 2 weeks, 5 mg BID the last 2 weeks if well-tolerated .  Control: placebo |
| **Outcomes** | Muscle tone by Ashworth scale; EDDS; MSFC; timed 25-foot walk; Nine-hole Peg Test; Paced Auditory Serial Addition Test, Fatigue Severity Scale; Medical Outcomes Study Short Form 36; Health-related QoL; daily VAS to assess spasticity, pain, tremor, fatigue, concentration, micturition, walking, vision, mood, participant's global impression. |
| **Notes** | No data could be used as the trial did not report which groups the participants were initially randomised to nor did they report data before the first crossover. |

Risk of bias table

| **Bias** | **Authors' judgement** | **Support for judgement** |
| --- | --- | --- |
| Random sequence generation (selection bias) | Unclear risk | Quote "randomised"  Judgment: probably done, but no description of randomisation sequence generation. |
| Allocation concealment (selection bias) | Unclear risk | Not mentioned |
| Blinding of participants and personnel (performance bias) | Low risk | Quote: "double-blind" and Patients received identical-appearing capsules containingdronabinol (THC Marinol ® , Unimed Pharmaceuticals,  Judgement: probably done, but not described how. |
| Blinding of outcome assessment (detection bias) | Low risk | To avoid unmasking, a “treating” physician was responsible for general medical care and safety management, whereas an “assessing” physician was responsible for the neurologic tests. |
| Incomplete outcome data (attrition bias) | Low risk | No participants were lost to follow-up |
| Selective reporting (reporting bias) | Low risk | No protocol obtained. The trial reported pain on VAS and serious adverse events. |
| Other bias | Unclear risk | The trial was "Supported by the Dutch Ministry of Health, Welfare and Sport." |

***Langford 2012***

| **Methods** | International, multi-centre, randomised, double-blind, placebo-controlled trial. |
| --- | --- |
| **Participants** | Eligible subjects were to have CNP due to MS, of at least 3 months duration. Subjects were also to have a sum score of at least 24 on a pain 0–10 point NRS on the last 6 days during the baseline period. In addition, their analgesic regimen was to be stable for at least 2 weeks preceding the study entry day.  Exclusion: Severe pain for other conditions (nociceptive pain, musculoskeletal pain including spasms, peripheral neuropathic, or psychogenic origin, trigeminal neuralgia. Significant psychiatric disorders (other than depression), renal, hepatic, cardiovascular or convulsive disorders, sensitivity to cannabis or cannabinoids. |
| **Interventions** | Experimental: Sativex (nasal spray combining tetrahydrocannabinol with cannabidiol), 1 pump delivered 2.7mg of THC and 2.5mg of CBD. Maximum of 12 sprays per 24hrs.  Control: Placebo. |
| **Outcomes** | Primary outcome: response to treatment defined as an improvement of 30% or more in patient's mean pain NRS from baseline to last week of treatment in phase A.  Secondary outcomes: Brief pain inventory-short form, subject global impression of change, sleep quality assessment. |
| **Notes** | There was a large descripency between the reporting of adverse events on clinicaltrials.gov and the main publication. We used the data from the main publication. |

Risk of bias table

| **Bias** | **Authors' judgement** | **Support for judgement** |
| --- | --- | --- |
| Random sequence generation (selection bias) | Low risk | Quote: "pre-determined computer-generated randomisation code"  Judgement: probably done |
| Allocation concealment (selection bias) | Low risk | Quote: "stratified by center, randomly permuted blocks of variable sizes"  Judgement: probably done |
| Blinding of participants and personnel (performance bias) | Low risk | Quote: "double-blind" and "placebo delivered excipient and colorants"  Judgement: probably done |
| Blinding of outcome assessment (detection bias) | Low risk | "Patients, investigators, and those assessing the data were therefore blinded to the patients’ treatment allocation" in addition to the description of the placebo. |
| Incomplete outcome data (attrition bias) | Unclear risk | 26 participants withdrew from the experimental group (adverse events n=14, withdrew consent n=3, lack of efficacy n=3, other n=6).  16 participants withdrew from the control group (adverse events n=9, withdrew consent n=2, lack of efficacy n=4, other n=1). The trial stated it used intention to treat data, but it was unclear how it imputed data for participants with missing data. |
| Selective reporting (reporting bias) | High risk | Protocol obtained <https://clinicaltrials.gov/ct2/show/NCT00391079>. The trial changed how it reported the primary outcome from the mean NRS score at the end of treatment to a change in NRS score and response to treatment, defined as an improvement of 30 % or more in patient’s mean pain NRS score from baseline to the last week of treatment. |
| Other bias | High risk | Quote: "Funded by GW Pharma Ltd., that covered the costs and had input into the writing of the paper" |

***Leocani 2014***

| **Methods** | Randomised, placebo-controlled, crossover trial. |
| --- | --- |
| **Participants** | Multiple sclerosis and clinical evidence of spasticity (modified Ashworth scale -MAS > 1). |
| **Interventions** | Experimental: sativex cannabis (tetrahydrocannabinol + cannabidiol, and small amounts of cannabinoids, terpenoids, and ethanol)  Control intervention: Placebo. |
| **Outcomes** | Spasticity on modified Ashworth scale, pain NRS, 10-minute walking distance, fatigue severity scale. |
| **Notes** |  |

Risk of bias table

| **Bias** | **Authors' judgement** | **Support for judgement** |
| --- | --- | --- |
| Random sequence generation (selection bias) | Unclear risk | Quote: "randomised"  Judgment: probably done, but no mention of random sequence generation. |
| Allocation concealment (selection bias) | Unclear risk | Not mentioned |
| Blinding of participants and personnel (performance bias) | Unclear risk | Quote: "double-blind fashion"  Judgement: probably done |
| Blinding of outcome assessment (detection bias) | Unclear risk | Not mentioned |
| Incomplete outcome data (attrition bias) | Unclear risk | Five patients dropped out, two during active treatment, three during placebo. |
| Selective reporting (reporting bias) | Unclear risk | No protocol obtained. |
| Other bias | High risk | Sponsored by Almirall S.A. (holder of the Sativex commercializator rights for EU ex-UK). Medication provided by GW Pharmaceuticals. |

***Levin 2017***

| **Methods** | Randomised, Parallel Assignment |
| --- | --- |
| **Participants** | Inclusion Criteria:   - Age 18 or older with an American Society of Anesthesiologists (ASA) physical status of I to III who are scheduled to undergo elective surgery under general anesthesia with pre-anesthesia consultation prior to surgery. - Subjects must be able to swallow study medication; - At a risk of postoperative nausea and vomiting of at least 61% percent, according to a simplified risk score, based on the presence of at least three of the following risk factors: female sex, nonsmoker status, anticipated use of postoperative opioid and previous PONV or motion sickness.   Exclusion Criteria:   - Subjects with clinically significant or unstable cardiac, respiratory, hepatic, renal, or other major organ system disease - Patients who will not be admitted to the PACU post-operatively (patients who are immediately transferred to the ICU) - Known sensitivity to marijuana or other cannabinoid agents - Psychotic illness or depression - Addiction to illicit substances or alcohol - Non-psychotic emotional disorders. - Pregnant or lactating - Subjects who suffer from chronic nausea and/or vomiting; - Has had treatment with any other investigational drug within 12 weeks prior to randomization - Subjects who, in the opinion of the investigator, would experience an unacceptable risk from administration of study drug |
| **Interventions** | Drug: Nabilone  Nabilone (0.5 mg) or placebo given preoperativelyOther Name: Cesamet  Drug: Placebo  Placebo Comparator: identical capsule containing placebo (single dose) given preoperatively |
| **Outcomes** | Primary Outcome Measures  : Incidence of postoperative nausea and/or vomiting [ Time Frame: Prior to discharge from postanesthesia care unit, an expected average of two hours ]  Secondary Outcome Measures : Number of antiemetic rescue medications given postoperatively. [ Time Frame: Prior to discharge from postanesthesia care unit, an expected average of two hours ]  Other Outcome Measures: Standardized score of nausea and/or vomiting severity if PONV occurs. [ Time Frame: Prior to discharge from postanesthesia care unit, an expected average of two hours ] Pain score during the immediate post-operative period. [ Time Frame: Prior to discharge from postanesthesia care unit, an expected average of two hours ] Use of intraoperative and postoperative opioids [ Time Frame: Prior to discharge from postanesthesia care unit, an expected average of two hours ] Rates of known side effects. [ Time Frame: Prior to discharge from postanesthesia care unit, an expected average of two hours ]Nabilone side effects include: drowsiness, vertigo, psychological high, dry mouth, depression, blurred vision, sensation disturbance, anorexia, headache, euphoria, and hallucinations (based on patient self-reporting). Time to discharge from the PACU. [ Time Frame: Prior to discharge from postanesthesia care unit, an expected average of two hours ] Rates of admission due to PONV [ Time Frame: Prior to discharge from postanesthesia care unit, an expected average of two hours ] Antiemetics given prophylactically by the anesthesiologist. [ Time Frame: Until discharge from postanesthesia care unit, an expected average of two hours ] |
| **Notes** |  |

Risk of bias table

| **Bias** | **Authors' judgement** | **Support for judgement** |
| --- | --- | --- |
| Random sequence generation (selection bias) | Low risk | An epidemiologist not directly involved in either the execution of the study or the analysis of the study results prepared a computer-generated restricted randomization schedule with a 1:1 allocation ratio and randomly varying block sizes |
| Allocation concealment (selection bias) | Low risk | To maintain allocation concealment, the research pharmacy used sequentially numbered sealed D. Levin et al. 123 opaque envelopes to assign and prepare the study drug or placebo for each enrolled patient in sequence on the day of surgery. |
| Blinding of participants and personnel (performance bias) | Low risk | To ensure blinding, the nabilone and placebo pills were identical in appearance. All study investigators, study coordinators, participants, clinicians, and data analysts were blinded to group allocation for the duration of the study. The research pharmacy maintained a sealed envelope for each enrolled patient. The envelopes were labelled with the subject number and contained information for emergency unblinding if needed. |
| Blinding of outcome assessment (detection bias) | Low risk | To ensure blinding, the nabilone and placebo pills were identical in appearance. All study investigators, study coordinators, participants, clinicians, and data analysts were blinded to group allocation for the duration of the study. The research pharmacy maintained a sealed envelope for each enrolled patient. The envelopes were labelled with the subject number and contained information for emergency unblinding if needed. |
| Incomplete outcome data (attrition bias) | Low risk | The primary intention-to-treat analysis included all patients who received the study drug, underwent the planned surgery, and had available outcomes data. Missing data were not imputed.  6 participants were lost to follow-up in the placebo group in total. The number of particpants that were lost to follow were below 5%. |
| Selective reporting (reporting bias) | Low risk | https://clinicaltrials.gov/ct2/show/NCT02115529 protocol obtained and all pre-specified outcomes reported including pain on NRS . |
| Other bias | Low risk | This trial was supported in part by a Physicians’ Services Incorporated Resident’s Research Grant. Valeant Canada donated the study medications and placebo. Neither Physicians’ Services Incorporated nor Valeant Canada had any role in the design and conduct of the study; the collection, management, analysis, and interpretation of the data; or the preparation, review, or approval of the manuscript. |

***Lichtman 2018***

| **Methods** | Phase 3, double blind, randomised, placebo-controlled trial. |
| --- | --- |
| **Participants** | Eligible patients had advanced cancer, were 18 years or older, had a clinical diagnosis of cancer-related pain that was unalleviated by an optimized maintenance dose of Step 3 opioid therapy. Opioid therapy was considered optimized if: 1) a dose increase was clinically inappropriate due to opioid-related side effects or 2) further efficacy benefit was not expected at higher doses (for the second definition, patients had to be receiving 90 mg morphine equivalents/day or more, inclusive of maintenance, and breakthrough opioids). The maintenance opioid was preferably a sustained-release formulation, but an around-the-clock immediate-release formulation was acceptable. To be eligible, patients also had to fulfill the following criteria on each of three consecutive days during the screening period: four or less opioid breakthrough analgesic episodes per day (averaged over the three days); a stable maintenance opioid therapy dose; average pain of four or more and eight or less on a 0 to 10 Numerical Rating Scale (NRS); and average pain scores on the NRS that did not change by more than two points (i.e., no more than a two-point difference between the highest and lowest scores, with all scores remaining between four and eight).  Key exclusion criteria included baseline use of morphine at >500 mg morphine equivalents/day (inclusive of maintenance and breakthrough opioids), current use of more than one type of breakthrough opioid analgesic, planned clinical interventions that would affect pain, and any history of schizophrenia or substance abuse. |
| **Interventions** | Experimental: nabiximol oral mucosal spray. One spray in the evening, gradully titrated by one additional spray per day, until unacceptable side effects or acceptable pain relief or maximum dose of 10 sprays per day. Titration lasted 14 days, after which patients continued on that dose for 21 days.  Control: placebo |
| **Outcomes** | Primary outcome: percent improvement from baseline to end of treatment in the following parameters: average pain NRS score, worst pain NRS score, sleep disruption NRS score  Secondary outcomes: maintenance, breakthrough, total opioid use per day in morphine equivalents.  Patients also completed: subject global impression of chance (SGIC); patient satisfaction questionnaire (PSQ), physician global impression of change (PGIC); constipation NRS |
| **Notes** |  |

Risk of bias table

| **Bias** | **Authors' judgement** | **Support for judgement** |
| --- | --- | --- |
| Random sequence generation (selection bias) | Unclear risk | Quote: "eligible patients were randomised 1:1".  Judgement: probably done, but no mention of randomisation sequence generation. |
| Allocation concealment (selection bias) | Unclear risk | Not mentioned. |
| Blinding of participants and personnel (performance bias) | Low risk | Quote: "nabiximols oral mucosal spray or matching placebo"  Judgement: probably done |
| Blinding of outcome assessment (detection bias) | Unclear risk | Not mentioned. |
| Incomplete outcome data (attrition bias) | High risk | 58 nabiximols patients and 48 placebo patients withdrew from the study. |
| Selective reporting (reporting bias) | Low risk | <https://clinicaltrials.gov/ct2/show/NCT01262651> protocol obtained and all pre-specified outcomes reported including pain on NRS and serious adverse events. |
| Other bias | High risk | This work was supported by Otsuka Pharmaceutical Development & Commercialization, Inc., Rockville, MD, USA. The efforts of A.H. Lichtman were supported by the Virginia Commonwealth University School of Pharmacy start-up funds. The authors declare no conflicts of interest |

***Lynch 2014***

| **Methods** | A Randomised, placebo-controlled, crossover pilot study. |
| --- | --- |
| **Participants** | Participants had to have neuropathic pain persisting for three months after completing chemotherapy with paclitaxel, vincristine, or cisplatin. The average seven day intensity of pain had to be 4 or more on an 11-point numeric rating scale. Participants also exhibited sensory abnormalities comprising allodynia, hyperalgesia, or hypesthesia. Concurrent analgesics had to be stable for 14 days before entry into the trial.  Exclusion criteria: ischemic heart disease, epilepsy, personal or family history of schizophrenia or psychotic disorder, substance abuse of dependency, pregnancy, medical condition that might compromise safety in the trial. |
| **Interventions** | Experimental: nabiximols, an oromucosal whole cannabis-based spray combining tetrahydrocannabinol with cannabidiol, minor cannabinoids and terpenoids, plus ethanol and propylene glycol excipients, and peppermint flavoring.  Control: Placebo that was packaged in exactly the same way, with a similar yellowish color and peppermint flavor. |
| **Outcomes** | Primary outcome: neuropathic pain measured on an 11-point NRS with anchors "no pain" or "pain as bad as you can imagine".  Secondary outcomes:   - SF36 - QST |
| **Notes** |  |

Risk of bias table

| **Bias** | **Authors' judgement** | **Support for judgement** |
| --- | --- | --- |
| Random sequence generation (selection bias) | Low risk | Quote: "computergenerated randomization schedule determined the order of treatment"  Judgement: probably done |
| Allocation concealment (selection bias) | Low risk | Quote: "participants and study staff were blinded to the randomization code"  Judgement: probably done |
| Blinding of participants and personnel (performance bias) | Low risk | Quote: "blinded" and "The placebo was packaged in exactly the same way, with similar yellowish color and peppermint flavor".  Judgement: probably done |
| Blinding of outcome assessment (detection bias) | Unclear risk | Not mentioned. |
| Incomplete outcome data (attrition bias) | Unclear risk | All partici"Eighteen participants entered the trial and 16 completed the Randomised, placebo-controlled, crossover protocol"pants completed the RCT. |
| Selective reporting (reporting bias) | Low risk | No protocol obtained. Pain on NRS and serious adverse events reported. |
| Other bias | Unclear risk | No conflicts of interest mentioned, but "GW Pharmaceuticals kindly supplied the medication used in this study." |

***Malik 2016***

| **Methods** | Baseline screening period, where patients were asked to maintain a daily chest pain diary for 2 weeks. On day 1, before randomisation, patients completed Short Form-36 (SF36) and Beck anxiety and depression inventories. Patients were randomised by the providing pharmacist based on 1:1 ratio. Patients were contacted every 3 days to verify medication compliance. At day 28, patiens underwent esophageal balloon distention test (EBDT) and completed questionnaries: chest pain symptoms; SF36; Beck anxiety and depression inventories. |
| --- | --- |
| **Participants** | Fulfilling diagnostic criteria for functional chest pain according to Rome 3.  All patients underwent cardiac evaluation that excluded a cardiac source for chest pain, and in many instances, after various empirical therapies including PPIs, TCA, and SSRI had proved to be ineffective for at least 3 months. Patients aged 18–75 years were included if they fulfilled diagnostic criteria of at least two weekly episodes of chest pain for the last 3 months, a normal cardiac evaluation (Stress test ± normal coronary angiogram), normal chest xray, upper GI endoscopy with normal esophageal biopsies, high resolution esophageal manometry, and a normal 24-hour pH impedance study (% fraction time of pH <4.0 was <4.5). All patients had to have evidence of esophageal hypersensitivity with an abnormal esophageal balloon distention test (EBDT).  Exclusion criteria: history of requiring narcotics, other pain medication; substance abuse; Barrett's esophagus or peptic stricure; significant physical or psychiatric comorbidity. |
| **Interventions** | Experimental group: dronabinol 5mg bid  Control group: Matching placebo bid |
| **Outcomes** | Primary outcome: frequency of chest pain episodes  Secondary outcomes:   - Esophageal balloon distention test - SF3 - Beck anxiety and depression inventory6 |
| **Notes** |  |

Risk of bias table

| **Bias** | **Authors' judgement** | **Support for judgement** |
| --- | --- | --- |
| Random sequence generation (selection bias) | Unclear risk | Quote: "patients were randomised by the providing pharmacist"  Judgement: probably done, but method of randomisation unclear. |
| Allocation concealment (selection bias) | Unclear risk | No mention of allocation concealment. |
| Blinding of participants and personnel (performance bias) | Low risk | Quote: "Double-blind" and "Placebo capsules were matched accordingly to resemble dronabinol capsules"  Judgement: probably done |
| Blinding of outcome assessment (detection bias) | Unclear risk | Not mentioned. |
| Incomplete outcome data (attrition bias) | Unclear risk | Three participants dropped out in each intervention group for unclear reasons. |
| Selective reporting (reporting bias) | Low risk | Protocol obtained, <https://clinicaltrials.gov/ct2/show/NCT01598207>, all pre-specified outcomes reported. However, no reporting of pain on NRS or VAS score. |
| Other bias | Low risk | No conflicts of interest.  Planned to include 40 participants, but only included 19. |

***Marková 2018***

| **Methods** | Randomised, parallel group, double-blind, placebo-controlled two-phase trial. |
| --- | --- |
| **Participants** | Tetrahydrocannabinol [THC]:cannabidiol [CBD] oromucosal spray (Sativex®) as add-on therapy to optimized standard antispasticity treatment in participants with moderate to severe multiple sclerosis (MS) spasticity.  **Inclusion criteria:**  1) adults ≥ 18 years of age  2) with a diagnosis of MS and existing MS spasticity symptoms for at least 12 months  3) moderate to severe MS spasticity defined as a score of ≥4 on the MS spasticity 0-10 numerical rating scale (NRS) scale  4) currently receiving optimized treatment with one or more oral antispasticity drugs (baclofen and/or tizanidine and/or dantrolene as monotherapy or in combination therapy) for at least 3 months prior to screening without adequate relief of MS spasticity symptoms  **Exclusion criteria:**  1) prior administration of THC:CBD spray  2) current consumption of cannabis herb or other cannabinoid-based drugs within 30 days prior to study entry  3) treatment with botulinum toxin injection for spasticity relief within the previous 6 months  4) medical history or family history of major psychiatric disorders other than depression  5) known or suspected history of a dependence disorder or heavy alcohol consumption  6) possibility of pregnancy or lactation; history of myocardial infarction or clinically significant cardiac dysfunction  7) clinically significant impaired renal function or impaired hepatic function |
| **Interventions** | In a single-blind, 4-week, trial period (Phase A), patients received THC:CBD spray as add-on therapy to optimized standard antispasticity medication.  Non-responer was defined as <20% improvement from baseline in the MS spasticity 0-10 NRS score. Non-responders were removed from the study.  Initial responders at 4 weeks entered a 1 to 4 week washout phase. Initial responders whose improvement in the MS spasticity NRS score during Phase A was reduced by ≥ 80% during the washout period were eligible for Phase B.  In Phase B, participants were randomised in a double-blind manner to treatment with THC:CBD spray or placebo for 12 weeks. Participants were advised to re-up-titrate their study medication to the optimal individual dose identified in Phase A, then to maintain the study treatment at this dose while allowing for adjustments according to the participant’s needs. |
| **Outcomes** | The primary efficacy endpoint was the proportion of responders after 12 weeks of randomised treatment in Phase B, where responder was defined as a patient who achieved ≥ 30% improvement in the MS spasticity 0-10 NRS score from Phase B baseline.  Secondary efficacy variables were measures of spasticity and associated symptoms during the 12-week randomised treatment period (Phase B):   - Change from baseline in MS spasticity 0-10 NRS score - Change from baseline in pain 0-10 NRS score - Change from baseline in modified Ashworth scale (MAS) score - Change from baseline in Expanded Disability Status Scale (EDSS) score.   Other secondary efficacy endpoints were:   - Barthel activities of daily living (ADL) index - Short form 36 quality of life (QoL) health survey (SF-36) - The number of patients with adverse events (AEs) and serious adverse events (SAEs) - Sleep disruption 0-10 NRS score - The numbers of AEs, SAEs, and treatment interruptions related to AEs; and discontinuation rates and the reasons for discontinuation were also recorded. |
| **Notes** |  |

Risk of bias table

| **Bias** | **Authors' judgement** | **Support for judgement** |
| --- | --- | --- |
| Random sequence generation (selection bias) | Unclear risk | Method of randomisation was not specified, but the trial was still presented as being randomised |
| Allocation concealment (selection bias) | Unclear risk | The trial was classified as randomised but the allocation concealment process was not described. |
| Blinding of participants and personnel (performance bias) | Unclear risk | The procedure of blinding was insufficiently described. |
| Blinding of outcome assessment (detection bias) | Unclear risk | It was not mentioned if the outcome assessors in the trial were blinded |
| Incomplete outcome data (attrition bias) | High risk | The trial used improper methods in dealing with the missing data: Missing data were handled using Last Observation Carried Forward (LOCF). |
| Selective reporting (reporting bias) | Unclear risk | A protocol was published before the trial was begun and outcomes in the protocol were reported on, however adverse events and serious adverse events were not mentioned outcomwa in the protocol. |
| Other bias | High risk | This study was sponsored by Almirall Hermal GmbH and Almirall S.A.  J Markovà has received fees as consultant or investigator in projects sponsored by Almirall  U Essner and A Lenschat are consultants for Almirall Hermal GmbH. B Akmaz and C Trompke are fulltime employees of Almirall Hermal GmbH. M Marinelli and C Vila are fulltime employees of Almirall S.A. |

***Narang 2008***

| **Methods** | Randomised, crossover group, double-blind, placebo-controlled two-phase trial. |
| --- | --- |
| **Participants** | **Inclusion criteria:**  1) Participants with chronic noncancer pain that were taking stable doses of opioid analgesics for longer than 6 months and reported pain of at least 4 on a 0 to 10 numeric rating scale.  2) Female participants with reproductive capacity were eligible if they agreed to use birth control measures during the study and had negative urine pregnancy tests.  3) Abstained from using marijuana for 1 month before participation in the study, although no formal testing was done to evaluate ongoing marijuana use.  **Exclusion criteria:**  1) Pain due to cancer.  2) Participants using a transdermal fentanyl patch or intrathecally administered opioid treatment, because participants would not be able to easily titrate their opioid dose during the trial.  3) Required opioid dosing more frequently than every 8 hours.  4) Unstable psychiatric disorder per investigator judgment.  5) Current substance abuse by self-report.  6) Involvement in active litigation, compensation, or disability issues. 7) Significant depression and/or anxiety (scores higher than 11 on the Hospital Anxiety and Depression Scale). |
| **Interventions** | The research design includes 2 studies; a doubleblinded, Randomised, placebo-controlled, single-dose study (Phase I) followed by an open-label, multidose extension study.  The single-dose phase was a double-blinded, Randomised, 3-treatment, 3-period, crossover trial. Subjects each received identically appearing placebo, 10 mg or 20 mg dronabinol capsules in 1 of 6 randomly allocated sequences. The 3 treatment visits were separated by a minimum of 3 days between each visit. |
| **Outcomes** | Primary outcome measure:   - Total Pain Relief at 8 hours (TOTPAR), ranging from 0=no relief of pain to 10=complete relief of pain - Average pain intensity and for the sum of pain intensity difference (SPID) for 8 hours after receiving the test drug.   Secondary outcome measures:  Patient satisfaction, side effects, dropout rate, adverse events, pain bothersomeness, changes in mood, changes in evoked pain (0 –10), evaluation of subject blinding, and plasma levels of THC. |
| **Notes** |  |

Risk of bias table

| **Bias** | **Authors' judgement** | **Support for judgement** |
| --- | --- | --- |
| Random sequence generation (selection bias) | Low risk | The Investigational Drug Service (IDS) Pharmacy of the hospital generated the randomization scheme (www.randomization.com). |
| Allocation concealment (selection bias) | Low risk | Subjects each received identically appearing placebo, 10 mg or 20 mg dronabinol capsules in 1 of 6 randomly allocated sequences |
| Blinding of participants and personnel (performance bias) | Low risk | Study personnel and subjects were blinded until all the subjects had completed the Phase I trial. |
| Blinding of outcome assessment (detection bias) | Unclear risk | It was not mentioned if the outcome assessors in the trial were blinded or the extent of blinding was insufficiently described. |
| Incomplete outcome data (attrition bias) | Low risk | Missing data were unlikely to make treatment effects depart from plausible values. |
| Selective reporting (reporting bias) | Unclear risk | No protocol was published and the outcome serious adverse events were not reported on. |
| Other bias | Unclear risk | Supported in part by an investigator-initiated grant from Solvay Pharmaceuticals, Inc. |

***NCT01606202***

| **Methods** | Randomised clinical trial, parallel assignment, double blinded |
| --- | --- |
| **Participants** | **Inclusion Criteria:**   - Gave informed consent for participation in the study. - Male or Female, aged 18 years or above. - Diagnosis of non-acute spinal cord injury, with central neuropathic pain not wholly relieved by current therapy. - Central neuropathic pain with a mean severity Numerical Rating Scale score at least four during last seven days of the baseline period. - Relatively stable neurology during the preceding six months. - Stable medication regimen during the preceding four weeks. - Agreement, if female and of child bearing potential or if male with a partner of child bearing potential, to ensure that effective contraception was used during the study and for three months thereafter. - Had not used cannabinoids for at least the preceding seven days and willing to abstain from any use of cannabinoids during the study. - Clinically acceptable laboratory results at Visit 2. - Ability (in the investigator's opinion) and willingness to comply with all study requirements. - Agreement for the UK Home Office, their general practitioner, and their consultant if appropriate, to be notified of their participation in the study.   **Exclusion Criteria:**   - History of schizophrenia, other psychotic illness, severe personality disorder or other significant psychiatric disorder other than depression associated with their underlying condition. - History of alcohol or substance abuse. - Severe cardiovascular disorder, such as ischaemic heart disease, arrhythmias (other than well controlled atrial fibrillation), poorly controlled hypertension or severe heart failure. - History of autonomic dysreflexia. - History of epilepsy. - If female, were pregnant or lactating, or were planning a pregnancy to occur during the course of the study. - Significant renal or hepatic impairment. - Elective surgery or other procedures requiring general anaesthesia scheduled to occur during the study. - Terminal illness or were considered inappropriate for placebo medication. - Any other significant disease or disorder which, in the opinion of the investigator, may have either put the subject at risk because of participation in the study, or may influenced the result of the study, or the subject's ability to participate in the study. - Regular levodopa therapy within the seven days leading to study entry. - If male, were receiving and were unwilling to stop sildenafil for the duration of the study. - Known or suspected hypersensitivity to cannabinoids or any of the excipients of the study medications. - Known or suspected adverse reaction to cannabinoids. Intention to travel internationally during the study. Intention to donate blood during the study. - Participation in another research study in the 12 weeks leading to study entry. Previous randomisation into this study. |
| **Interventions** | Experimental: GW-1000-02 Active treatment.  Drug: GW-1000-02  Contained delta-9-tetrahydrocannabinol (THC) (27 mg/ml):cannabidiol (CBD) (25 mg/ml) as extract of Cannabis sativa L., with peppermint oil, 0.05% (v/v), in ethanol:propylene glycol (50:50) excipient. Each actuation delivered 100 μl (THC 2.7 mg and CBD 2.5 mg). The maximum permitted dose of study medication was eight actuations in any three-hour period, and 48 actuations in any 24 hour period.Other Name: Sativex  Placebo Comparator: Placebo  Placebo control.  Drug: Placebo  Contained peppermint oil, 0.05% (v/v), quinoline yellow, 0.005% (w/v), sunset yellow, 0.0025% (w/v), in ethanol:propylene glycol (50:50) excipient. Each actuation delivered 100 μl. The maximum permitted dose of study medication was eight actuations in any three-hour period, and 48 actuations in any 24 hour period. |
| **Outcomes** | **Primary Outcome Measures**  Change From Baseline in Mean Central Neuropathic Pain 11-Point Numerical Rating Scale Scores at the End of Treatment (up to 51 Days). [ Time Frame: Up to 51 days ]The Central Neuropathic Pain Numerical Rating Scale score was recorded three times daily, in the morning (on waking), at lunchtime and in the evening using the scale, 0 = 'No Pain' and 10 = 'Worst Possible Pain'. Patients were instructed to relate 'No Pain' to the time before the start of their spinal cord injury. End of Treatment was defined as the mean of the last seven days in the study or the mean of the last three days if the subject withdrew. A negative value indicates an improvement in pain score from baseline.  **Secondary Outcome Measures :**  Change From Baseline in the Mean Percentage of Days on Which Escape Medication Was Used at the End of Treatment [ Time Frame: Up to 51 days ]The percentage of days that subjects used escape medication was analysed and is presented as the mean change from baseline at the end of treatment. A negative value from baseline indicates an improvement.  Change From Baseline in Mean Spasm Severity Numerical Rating Scale Score at the End of Treatment [ Time Frame: Up to 51 days ]Each day, just before going to bed, patients recorded in their patient diary whether they experienced any spasms that day and, if yes, recorded the overall level of the spasm(s) experienced using an Numerical Rating Scale spasm scale ranging from 0 = "Mildest ever spasm" to 10 = "Worst ever spasm". The mean spasm severity scores were summarised and analysed analogously to the primary endpoint. A negative value from baseline indicates an improvement.  Change From Baseline in the Percentage of Days on Which Spasm Was Experienced at the End of Treatment [ Time Frame: Up to 51 days ]Each day, just before going to bed, patients recorded in their patient diary whether they experienced any spasms that day. The percentage of days on which spasm was experienced were summarised and analysed analogously to the primary endpoint. A negative value from baseline indicates an improvement.  Change From Baseline in Mean Spasticity Severity Numerical Rating Scale Scores the End of Treatment. [ Time Frame: 0 - 51 days ]Each day at bed time patients recorded whether they experienced any spasticity that day and, if yes, the overall level of spasticity experienced was quantified using an Numerical Rating Scale from 0 = "Mildest ever spasticity" to 10 = "Worst ever spasticity". The mean spasticity severity scores and the changes from baseline to End of Treatment were to be calculated. A negative value indicates an improvement from baseline.  Change From Baseline in the Percentage of Days on Which Spasticity Was Experienced at the End of Treatment [ Time Frame: Up to 51 days ]Each day, just before going to bed, patients recorded in their patient diary whether they had experienced any spasticity that day or not. The percentage of days on which spasticity was experienced (spasticity incidence) was calculated and summarised analogously to the primary efficacy parameter of Numerical Rating Scale pain score. A negative value from baseline indicates an improvement.  Change From Baseline in Modified Ashworth Scale Score at the End of Treatment [ Time Frame: Up to 51 days ]The Modified Ashworth Scale is a five-point scale conducted on four pre-identified muscle groups. Only the lower limb was assessed because not all Spinal Cord Injury patients upper limb disability. The assessor used the Modified Ashworth scale ranging from 0 ("No increase in muscle tone") to 4 ("Affected part(s) rigid in flexion or extension") to rate the muscle tone for knee and ankle for the left and right sides separately at a pre-dose visit and at the end of treatment. The average of the four individual scores and was taken. A negative value indicates an improvement from baseline.  Change From Baseline in the Mean Short Orientation Memory Function Concentration Test Score at the End of Treatment [ Time Frame: Up to 51 days ]Patients were asked at baseline and end of treatment, to complete the Short Orientation Memory Function Concentration Test as a measure of cognitive function. The minimum score is 0 and maximum of 28 which denoted good cognitive function .A negative value from baseline indicates a deterioration.  Change From Baseline in Mean Spitzer Quality of Life Index Score at the End of Treatment [ Time Frame: Up to 51 days ]The Spitzer Quality of Life Index questionnaire consists of five sections, relating to activity, daily living, health, support and outlook. Each section has three choices (numbered 1, 2 and 3) and the patient is required to choose the one that best describes their quality of life during the last week. Choice 1 is scored 2, choice 2 is scored 1 and choice 3 is scored 0. The total Spitzer is the unweighted sum of the five scores. The scale is 0 (bad) to 10 (good). A positive value indicates an improvement from baseline.  Change From Baseline in the Mean Caregiver Strain Index Score at the End of Treatment [ Time Frame: Up to 51 days ]Carers were asked at baseline and end of treatment to complete the Caregiver Strain Index, as a measure of the strain they felt from being a carer, the maximum possible score being 13. A negative value from baseline indicates an improvement.  Number of Subjects Who Reported an Improvement in Their Overall Condition in the Patient Global Impression of Change at the End of Treatment [ Time Frame: Up to 51 days ]The Patient Global Impression of Change asked patients to give their impression of the overall change in their condition during the study at the end of treatment using the following scale: 1 = Very Much Improved, 2 = Much Improved, 3 = Minimally Improved, 4 = No Change, 5 = Minimally Worse, 6 = Much Worse, 7 = Very Much Worse. The number of patients who scored their condition as 1, 2, or 3 (improved) is presented.  Change From Baseline in the Mean Brief Pain Inventory Score at the End of Treatment [ Time Frame: 0 - 51 days ]The Brief Pain Inventory is a 14-item questionnaire that asks patients to rate pain over the prior week and the degree to which it interferes with activities on a 0 to 10 scale, where 0=no pain and 10=pain as bad as you can imagine. Severity is measured as worst pain, least pain, average pain, and pain right now. The severity composite score is calculated as the arithmetic mean of the four severity items(range 0-10). A negative value indicates an improvement in worst pain score from baseline.  Change From Baseline in the Mean Sleep Disturbance Numerical Rating Scale Score at the End of Treatment [ Time Frame: 0 - 51 days ]Each day patients recorded in their patient diary whether they woke during the previous night using the following scoring system: 0 = No, 1 = Once, 2 = Twice, 3 = More than twice, 4 = Awake most of the night. A negative value indicates an improvement from baseline.  Incidence of Adverse Events as a Measure of Patient Safety. [ Time Frame: Up to 61 days ]The number of patients who experienced an adverse event during the study is presented. |
| **Notes** |  |

Risk of bias table

| **Bias** | **Authors' judgement** | **Support for judgement** |
| --- | --- | --- |
| Random sequence generation (selection bias) | Unclear risk | Unclear risk: If the method of randomisation was not specified, but the trial was still presented as being randomised. |
| Allocation concealment (selection bias) | Unclear risk | Uncertain risk: If the trial was classified as randomised but the allocation concealment process was not described. |
| Blinding of participants and personnel (performance bias) | Unclear risk | Uncertain risk: If the procedure of blinding was insufficiently described. |
| Blinding of outcome assessment (detection bias) | Unclear risk | Uncertain risk of bias: If it was not mentioned if the outcome assessors in the trial were blinded or the extent of blinding was insufficiently described. |
| Incomplete outcome data (attrition bias) | Unclear risk | Uncertain risk of bias: If there was insufficient information to assess whether missing data were likely to induce bias on the results. |
| Selective reporting (reporting bias) | Low risk | Reporting pain assessment on VAS or NRS and serious adverse events will grant the trial a grade of low risk of bias. |
| Other bias | Unclear risk | Sponsor: GW Pharmaceuticals Ltd. |

***Nurmikko 2007***

| **Methods** | Randomised, double-blind, placebo-controlled parallel group study. |
| --- | --- |
| **Participants** | **Inclusion criteria:**  1) Patients had to have a current history of unilateral peripheral neuropathic pain and allodynia.  2) Age 18 or over, male or female  3) A history of at least 6 months duration of pain due to a clinically identifiable nerve lesion  4) Demonstrate mechanical allodynia and impaired sensation within the territory of affected nerve(s) on clinical examination  5) Patients with complex regional pain syndrome (CRPS) were eligible if they showed evidence of peripheral nerve lesion (diagnosed as CRPS type II)  6) A baseline severity score of at least 4 on the numerical rating scale for spontaneous pain for at least 4 of 7 days in the baseline week  7) A stable medication regimen of analgesics for at least 2 weeks prior to study entry  8) Female patients of child bearing potential and male patients whose partner was of child bearing potential had to agree to use effective contraception  9) Willing for his or her name to be notified to the UK Home Office  **Exclusion criteria:**  1) Cannabinoid use (cannabis, Marinol (synthetic THC) or nabilone (synthetic cannabinoid analogue)) at least 7 days before randomisation. Subjects were required to abstain from use of cannabis during the study  2) Schizophrenia, psychosis, or other major psychiatric condition beyond depression with underlying condition  3) Concomitant severe non-neuropathic pain or the presence of cancer related neuropathic pain or from diabetes mellitus  4) Known history of alcohol or substance abuse  5) Severe cardiovascular condition, poorly controlled hypertension, epilepsy, pregnancy, lactation, significant hepatic or renal impairment  6) Scheduled surgery or anaesthesia  7) Terminal illness or subjects inappropriate for placebo therapies  8) Known hypersensitivity to cannabinoids  9) Participation within a trial in the last 12 weeks |
| **Interventions** | Patients were screened to determine eligibility and completed baseline diary assessments of daily pain intensity and sleep disturbance scores in the 7–10 days prior to first treatment assignment. After eligibility was confirmed, patients were assigned to the next sequential randomisation number within each centre.  Initial dosing was under clinical supervision at the study site. A maximum of 8 sprays were administered over 2 h with Intoxication VAS and vital signs checked at regular intervals.  After satisfactory completion of initial dosing, patients began home dose titration and were allowed a maximum dose of 8 sprays per 3-hour interval and a maximum of 48 sprays per 24 h. At the next visit (after 7–10 days) titration, compliance and adverse events were reviewed, and patients advised on how to optimise dosing for the rest of the study period. Patients kept a diary from the screening visit until end of treatment in which they recorded daily their pain and sleep scores (on the appropriate NRS), as well as adverse events and the number of sprays used.  Maximum permitted dose was 48 actuations (THC 130 mg: CBD 120 mg) in 24 hours.  The neuropathic pain intensity NRS score at baseline was defined as the mean of all diary entries from Day 7 to Day 1 and, for the end of treatment score, the mean of all diary entries during the last 7 days in the study, or the last 3 days in the event of withdrawal. |
| **Outcomes** | The primary outcome measure:   - Change from baseline on a numerical rating scale (NRS) of mean intensity of global neuropathic pain.   Secondary outcome measures:   - The composite score calculated from the Neuropathic Pain Scale (NPS) - Tests for mechanical allodynia, - A four-step verbal rating scale for sleep disturbance (see below) - The Pain Disability Index (PDI) - The Patient Global Impression of Change (PGIC) of both pain and allodynia - The General Health Questionnaire (GHQ-12)   Adverse events were collected at each clinic visit, and haematology, clinical chemistry and ECG monitored at the beginning and end of the study. |
| **Notes** |  |

Risk of bias table

| **Bias** | **Authors' judgement** | **Support for judgement** |
| --- | --- | --- |
| Random sequence generation (selection bias) | Low risk | The randomisation schedule had a 1:1 treatment allocation ratio with randomly permuted blocks stratified by centre and was generated using a computer based pseudo-random number algorithm. The randomisation schedule was held by the sponsor with a copy inpatient-specific sealed envelopes sent to the pharmacy in each centre. Once the patient’s eligibility was confirmed, they were assigned to the next sequential randomisation number within each centre |
| Allocation concealment (selection bias) | Low risk | The placebo medication was identical in composition, appearance, odour and taste with the study medication but without cannabis extract. That the smell and taste of the cannabinoid preparation might lead to unblinding was averted by disguising them with addition of peppermint oil to both preparations. All medication was provided in identical amber vials, packaged and labelled by the sponsor. |
| Blinding of participants and personnel (performance bias) | Low risk | The participants and the treatment providers were blinded to intervention allocation and this was described. |
| Blinding of outcome assessment (detection bias) | Unclear risk | It was not mentioned if the outcome assessors in the trial were blinded or the extent of blinding was insufficiently described. |
| Incomplete outcome data (attrition bias) | Low risk | The primary analysis for the primary and secondary endpoints was performed on the intention-to-treat (ITT) population. |
| Selective reporting (reporting bias) | Low risk | The protocol was published after the trial has begun, reporting pain assessment on VAS or NRS and serious adverse events |
| Other bias | Unclear risk | GW Pharma acted as the sponsor of the study, provided the medication, participated in the study design, co-ordinated the study between centres and carried out the first set of analyses. |

***Ostenfeld 2011 (100 mg)***

| **Methods** | Randomised, doubleblind, placebo-controlled study incorporating 4 parallel groups |
| --- | --- |
| **Participants** | **Inclusion criteria:**  1) Aged 18 to 50 years.  2) In general good health  3) Scheduled for surgical extraction of up to 4 third molar teeth under local anesthesia  4) At least 1 of which was required to be fully or partially impacted in the mandible requiring bone removal  **Exclusion criteria:**  1) Previous allergic reactions to drugs or foods  2) Presence of significant organ disease or mental illness  3) Pregnant or lactating women, or women of childbearing potential who were not using adequate contraception as defined by the protocol  4) Previous exposure to either non-steroidal antiinflammatory drugs or COX 2 inhibitors within 48 hours or 5 half-lives before the start of surgery  5) Intolerance of paracetamol or opioid-based rescue medication  6) Concomitant use of other drugs  7) History of either drug or alcohol abuse or positive prestudy drug/alcohol screen. |
| **Interventions** | After baseline assessments participants were Randomised to 1 of 4 possible treatment regimens: GW842166 800 mg preoperative and placebo postoperative; GW842166 100 mg preoperative and placebo postoperative; Ibuprofen 800mg preoperative and Ibuprofen 400 mg postoperative; Placebo preoperative and Placebo postoperative.  It was anticipated that the duration of the dental surgery could last up to 1 hour, depending on the number of teeth being extracted. Surgery was performed under local anesthetic using 2% lidocaine (with 1:80,000 epinephrine). Nitrous oxide in conjunction with the local anesthetic was permitted.  Postoperative treatments were to be administered 4 hours after the preoperative dose.  A rescue analgesic (co-codamol 15/500: paracetamol 500 mg, codeine phosphate 15 mg) was available if pain became intolerable. Patients were not permitted to receive other analgesics or anti-inflammatory drugs.  Patients were asked to wait at least 90 minutes after completion of surgery before requesting rescue analgesia.  Poststudy follow-up visits occurred approximately 48 hours postdose and at 14 to 21 days postdose. Pain intensity was recorded predose, at 1 hour, every 15 minutes from 2 to 4 hours, and at 5, 6, 7, 8, 9, and 10 hours postsurgery. |
| **Outcomes** | Primary outcome:  Weighted mean of the pain intensity over the 10 hours post-surgery as measured by the VAS (Visual Analogue Scale) Timeframe: over the 10 hours post-surgery.  Secondary outcomes:   - Weighted mean of the pain intensity over the 10 hours post-surgery,measured by the Verbal Rating Scale -VAS and VRS mean pain scores up to 10 hours post-surgery. - Elapsed time from study drug administration to rescue analgesic request - Patient global evaluation before rescue medication use and at 10 and 24 hours postdose, - The proportion of patients requiring rescue medication. |
| **Notes** |  |

Risk of bias table

| **Bias** | **Authors' judgement** | **Support for judgement** |
| --- | --- | --- |
| Random sequence generation (selection bias) | Unclear risk | The method of randomisation was not specified, but the trial was still presented as being randomised. |
| Allocation concealment (selection bias) | Unclear risk | The trial was classified as randomised but the allocation concealment process was not described. |
| Blinding of participants and personnel (performance bias) | Unclear risk | The procedure of blinding was insufficiently described. |
| Blinding of outcome assessment (detection bias) | Unclear risk | It was not mentioned if the outcome assessors in the trial were blinded or the extent of blinding was insufficiently described. |
| Incomplete outcome data (attrition bias) | Low risk | Efficacy analyses were performed on the intent-to-treat population and included all Randomised patients, who took at least 1 dose of study medication and who had at least 1 postdose assessment. |
| Selective reporting (reporting bias) | Low risk | The protocol was published before and the outcomes specified in the protocol were reported on. |
| Other bias | Unclear risk | The authors declare no conflict of interest. Sponsored by GlaxoSmithKline |

***Ostenfeld 2011 (800 mg)***

| **Methods** | Randomised, doubleblind, placebo-controlled study incorporating 4 parallel groups |
| --- | --- |
| **Participants** | **Inclusion criteria:**  1) Aged 18 to 50 years.  2) In general good health  3) Scheduled for surgical extraction of up to 4 third molar teeth under local anesthesia  4) At least 1 of which was required to be fully or partially impacted in the mandible requiring bone removal  **Exclusion criteria:**  1) Previous allergic reactions to drugs or foods  2) Presence of significant organ disease or mental illness  3) Pregnant or lactating women, or women of childbearing potential who were not using adequate contraception as defined by the protocol  4) Previous exposure to either non-steroidal antiinflammatory drugs or COX 2 inhibitors within 48 hours or 5 half-lives before the start of surgery  5) Intolerance of paracetamol or opioid-based rescue medication  6) Concomitant use of other drugs  7) History of either drug or alcohol abuse or positive prestudy drug/alcohol screen. |
| **Interventions** | After baseline assessments participants were Randomised to 1 of 4 possible treatment regimens: GW842166 800 mg preoperative and placebo postoperative; GW842166 100 mg preoperative and placebo postoperative; Ibuprofen 800mg preoperative and Ibuprofen 400 mg postoperative; Placebo preoperative and Placebo postoperative.  It was anticipated that the duration of the dental surgery could last up to 1 hour, depending on the number of teeth being extracted. Surgery was performed under local anesthetic using 2% lidocaine (with 1:80,000 epinephrine). Nitrous oxide in conjunction with the local anesthetic was permitted.  Postoperative treatments were to be administered 4 hours after the preoperative dose.  A rescue analgesic (co-codamol 15/500: paracetamol 500 mg, codeine phosphate 15 mg) was available if pain became intolerable. Patients were not permitted to receive other analgesics or anti-inflammatory drugs.  Patients were asked to wait at least 90 minutes after completion of surgery before requesting rescue analgesia.  Poststudy follow-up visits occurred approximately 48 hours postdose and at 14 to 21 days postdose. Pain intensity was recorded predose, at 1 hour, every 15 minutes from 2 to 4 hours, and at 5, 6, 7, 8, 9, and 10 hours postsurgery. |
| **Outcomes** | Primary outcome:  Weighted mean of the pain intensity over the 10 hours post-surgery as measured by the VAS (Visual Analogue Scale) Timeframe: over the 10 hours post-surgery.  Secondary outcomes:   - Weighted mean of the pain intensity over the 10 hours post-surgery,measured by the Verbal Rating Scale -VAS and VRS mean pain scores up to 10 hours post-surgery. - Elapsed time from study drug administration to rescue analgesic request - Patient global evaluation before rescue medication use and at 10 and 24 hours postdose, - The proportion of patients requiring rescue medication. |
| **Notes** |  |

Risk of bias table

| **Bias** | **Authors' judgement** | **Support for judgement** |
| --- | --- | --- |
| Random sequence generation (selection bias) | Unclear risk | The method of randomisation was not specified, but the trial was still presented as being randomised. |
| Allocation concealment (selection bias) | Unclear risk | The trial was classified as randomised but the allocation concealment process was not described. |
| Blinding of participants and personnel (performance bias) | Unclear risk | The procedure of blinding was insufficiently described. |
| Blinding of outcome assessment (detection bias) | Unclear risk | It was not mentioned if the outcome assessors in the trial were blinded or the extent of blinding was insufficiently described. |
| Incomplete outcome data (attrition bias) | Low risk | Efficacy analyses were performed on the intent-to-treat population and included all Randomised patients, who took at least 1 dose of study medication and who had at least 1 postdose assessment. |
| Selective reporting (reporting bias) | Low risk | The protocol was published before and the outcomes specified in the protocol were reported on. |
| Other bias | Unclear risk | The authors declare no conflict of interest. Sponsored by GlaxoSmithKline |

***Portenoy 2012 (1 - 4 sprays)***

| **Methods** | Randomised, doubleblind, placebo-controlled, parallel group, graded-dose design |
| --- | --- |
| **Participants** | **Inclusion criteria:**  1) Adult patients were eligible for the study if they had active cancer and chronic pain that was moderate or severe despite a stable opioid regimen that could not be made more effective by further opioid dose titration.  **Exclusion criteria:**  1) If they had a major psychiatric or cardiovascular disorder, epilepsy, or significant renal or hepatic impairment, or if they were pregnant, lactating or not using adequate contraception.  2) Patients who had received or who were due to receive therapies that were expected to change the pain (such as radiotherapy, or chemotherapy or hormonal therapy) also were excluded.  3) Patients who were currently using or had used marijuana, cannabinoidbased medications or rimonabant within 30 days of study entry, and were unwilling to abstain for the duration of the study, were excluded.  4) Patients receiving long-term methadone therapy for pain were not eligible because of concerns that its potency relative to other opioid agonists may vary with dose, rendering analyses using morphine equivalent milligrams less reliable. |
| **Interventions** | The study timeline included a 5- to 14-day baseline period, randomization, a 5-week titration and treatment period, and a poststudy visit after 2 weeks. The maximum duration was 9 weeks.  After randomization, patients entered a titration and treatment phase, which included a 1-week blinded dose titration period followed by 4 weeks of stable dosing.  Throughout this phase, patients self-administered the study drug (either nabiximols or an identicallyappearing placebo) as an oromucosal spray delivered using a pump.  Patients randomly assigned to Group 1 (low dose) were instructed to titrate the study medication to between 1 and 4 sprays per day. Those assigned to Group 2 (medium dose) titrated the number of sprays to between 6 and 10 sprays per day, and those assigned to Group 3 (high dose) titrated to between 11 and 16 sprays per day. |
| **Outcomes** | Primary Outcome Measures   1. Number of Patients With at Least 30% Improvement in Numerical Rating Scale (NRS) Average Pain Score From Baseline. Time Frame: 5 Weeks: Baseline (first 3 days) - Week 5 (last 3 days)   Secondary Outcome Measures   1. Change in Cumulative Average Pain Response Curves [ Time Frame: Baseline to end of treatment (Week 5) ]The cumulative response to treatment is the percentage changes from baseline in the mean NRS pain score as defined as the 30% response.The pain NRS was completed at the same time each day, i.e. bedtime in the evening. The patient was asked "on a scale of '0 to 10', please indicate the number that best describes your pain or average pain in the last 24 hours" 2. Change in Mean Daily NRS Pain Score (Average Pain). [ Time Frame: 5 Weeks: Baseline (first 3 days) - End of Treatment (last 3 days of week 5) ]The average pain NRS was complete at the same time each day, i.e. bedtime in the evening. The patient was asked "on a scale of '0 to 10', please indicate the number that best describes your pain or average pain in the last 24 hours" where 0 = no pain and 10 = pain as bad as you can imagine. 3. Change in Mean Daily NRS Pain Score (Worst Pain). [ Time Frame: 5 Weeks: Baseline (first 3 days) - End of Treatment (last 3 days of week 5) ]The worst pain NRS was completed at the same time each day, i.e. bedtime in the evening. The patient was asked "on a scale of '0 to 10', please indicate the number that best describes your worst pain in the last 24 hours" where 0 = no pain and 10 = pain as bad as you can imagine.. 4. Change in Sleep Disruption NRS [ Time Frame: 5 Weeks: Baseline - End of Treatment (Last 3 days of Week 5) ]The sleep disruption NRS was completed at the same time each day, i.e. bedtime in the evening. The patient was asked "on a scale of '0 to 10', please indicate how your pain disrupted your sleep last night?" where 0 = did not disrupt sleep and 10 = completely disrupted (unable to sleep at all). 5. Change in Brief Pain Inventory - Short Form (BPI-SF) [ Time Frame: Baseline (Visit 2) and End of Treatment (End of Week 5 or premature termination) ]The BPI-SF is a 14-item questionnaire that asks patients to rate pain over the prior week and the degree to which it interferes with activities on a 0 to 10 scale, where 0=no pain and 10=pain as bad as you can imagine. 6. Change in Patient Assessment of Constipation Quality of Life (PAC-QoL) [ Time Frame: Baseline (Visit 2) and End of Treatment (Week 5 or premature termination) ]The PAC-QoL questionnaire consists of 28 questions divided into the following areas: 4 questions on physical discomfort, 8 questions on psychosocial discomfort, 11 questions on worries/concerns and 5 questions on satisfaction. 7. Change in Patient Global Impression of Change - PGIC [ Time Frame: End of Week 5 ]A 7-point Likert-type scale was used, with the question: 'Please assess the status of your pain due to cancer since entry into the study using the scale below' with the markers "very much improved, much improved, slightly improved, no change, slightly worse, much worse or very much worse". 8. Change in Montgomery Asberg Depression Rating Scale (MADRS) [ Time Frame: Baseline and End of Treatment (Week 5 or premature termination) ]The MADRS comprises of 10 questions that are completed by the patient to determine their depression level. The MADRS was completed at Visit 2 (Baseline) prior to receiving the study drug and at Visit 4 (Week 5 or premature termination). Each item is scored on a 0-6 scale , where 0=no sadness to 6=extreme and continuous gloom and despondency, and the MADRS score is the sum of the 10 item scores (range 0-60). |
| **Notes** |  |

Risk of bias table

| **Bias** | **Authors' judgement** | **Support for judgement** |
| --- | --- | --- |
| Random sequence generation (selection bias) | Low risk | Patients were randomly assigned by computer using a block approach, first to 1 of 3 dose groups, and then within each group, to either active drug or placebo |
| Allocation concealment (selection bias) | Unclear risk | The allocation to active drug or placebo was in a 3:1 ratio. The randomization was stratified by region (North America/Rest of the World). |
| Blinding of participants and personnel (performance bias) | Unclear risk | The procedure of blinding was insufficiently described. |
| Blinding of outcome assessment (detection bias) | Unclear risk | It was not mentioned if the outcome assessors in the trial were blinded or the extent of blinding was insufficiently described. |
| Incomplete outcome data (attrition bias) | High risk | The efficacy analyses were intent-to-treat. All patients who were Randomised and received at least 1 dose of study medication were entered into the analyses. Missing data for the efficacy endpoints were imputed using the last observation carry forward (LOCF) method. |
| Selective reporting (reporting bias) | Low risk | The protocol was published before and the outcomes specified in the protocol were reported on. |
| Other bias | Unclear risk | This study was funded by GW Pharmaceuticals and Otsuka. |

***Portenoy 2012 (11 - 16 sprays)***

| **Methods** | Randomised, doubleblind, placebo-controlled, parallel group, graded-dose design |
| --- | --- |
| **Participants** | **Inclusion criteria:**  1) Adult patients were eligible for the study if they had active cancer and chronic pain that was moderate or severe despite a stable opioid regimen that could not be made more effective by further opioid dose titration.  **Exclusion criteria:**  1) If they had a major psychiatric or cardiovascular disorder, epilepsy, or significant renal or hepatic impairment, or if they were pregnant, lactating or not using adequate contraception.  2) Patients who had received or who were due to receive therapies that were expected to change the pain (such as radiotherapy, or chemotherapy or hormonal therapy) also were excluded.  3) Patients who were currently using or had used marijuana, cannabinoidbased medications or rimonabant within 30 days of study entry, and were unwilling to abstain for the duration of the study, were excluded.  4) Patients receiving long-term methadone therapy for pain were not eligible because of concerns that its potency relative to other opioid agonists may vary with dose, rendering analyses using morphine equivalent milligrams less reliable. |
| **Interventions** | The study timeline included a 5- to 14-day baseline period, randomization, a 5-week titration and treatment period, and a poststudy visit after 2 weeks. The maximum duration was 9 weeks.  After randomization, patients entered a titration and treatment phase, which included a 1-week blinded dose titration period followed by 4 weeks of stable dosing.  Throughout this phase, patients self-administered the study drug (either nabiximols or an identicallyappearing placebo) as an oromucosal spray delivered using a pump.  Patients randomly assigned to Group 1 (low dose) were instructed to titrate the study medication to between 1 and 4 sprays per day. Those assigned to Group 2 (medium dose) titrated the number of sprays to between 6 and 10 sprays per day, and those assigned to Group 3 (high dose) titrated to between 11 and 16 sprays per day. |
| **Outcomes** | Primary Outcome Measures   1. Number of Patients With at Least 30% Improvement in Numerical Rating Scale (NRS) Average Pain Score From Baseline. Time Frame: 5 Weeks: Baseline (first 3 days) - Week 5 (last 3 days)   Secondary Outcome Measures   1. Change in Cumulative Average Pain Response Curves [ Time Frame: Baseline to end of treatment (Week 5) ]The cumulative response to treatment is the percentage changes from baseline in the mean NRS pain score as defined as the 30% response.The pain NRS was completed at the same time each day, i.e. bedtime in the evening. The patient was asked "on a scale of '0 to 10', please indicate the number that best describes your pain or average pain in the last 24 hours" 2. Change in Mean Daily NRS Pain Score (Average Pain). [ Time Frame: 5 Weeks: Baseline (first 3 days) - End of Treatment (last 3 days of week 5) ]The average pain NRS was complete at the same time each day, i.e. bedtime in the evening. The patient was asked "on a scale of '0 to 10', please indicate the number that best describes your pain or average pain in the last 24 hours" where 0 = no pain and 10 = pain as bad as you can imagine. 3. Change in Mean Daily NRS Pain Score (Worst Pain). [ Time Frame: 5 Weeks: Baseline (first 3 days) - End of Treatment (last 3 days of week 5) ]The worst pain NRS was completed at the same time each day, i.e. bedtime in the evening. The patient was asked "on a scale of '0 to 10', please indicate the number that best describes your worst pain in the last 24 hours" where 0 = no pain and 10 = pain as bad as you can imagine.. 4. Change in Sleep Disruption NRS [ Time Frame: 5 Weeks: Baseline - End of Treatment (Last 3 days of Week 5) ]The sleep disruption NRS was completed at the same time each day, i.e. bedtime in the evening. The patient was asked "on a scale of '0 to 10', please indicate how your pain disrupted your sleep last night?" where 0 = did not disrupt sleep and 10 = completely disrupted (unable to sleep at all). 5. Change in Brief Pain Inventory - Short Form (BPI-SF) [ Time Frame: Baseline (Visit 2) and End of Treatment (End of Week 5 or premature termination) ]The BPI-SF is a 14-item questionnaire that asks patients to rate pain over the prior week and the degree to which it interferes with activities on a 0 to 10 scale, where 0=no pain and 10=pain as bad as you can imagine. 6. Change in Patient Assessment of Constipation Quality of Life (PAC-QoL) [ Time Frame: Baseline (Visit 2) and End of Treatment (Week 5 or premature termination) ]The PAC-QoL questionnaire consists of 28 questions divided into the following areas: 4 questions on physical discomfort, 8 questions on psychosocial discomfort, 11 questions on worries/concerns and 5 questions on satisfaction. 7. Change in Patient Global Impression of Change - PGIC [ Time Frame: End of Week 5 ]A 7-point Likert-type scale was used, with the question: 'Please assess the status of your pain due to cancer since entry into the study using the scale below' with the markers "very much improved, much improved, slightly improved, no change, slightly worse, much worse or very much worse". 8. Change in Montgomery Asberg Depression Rating Scale (MADRS) [ Time Frame: Baseline and End of Treatment (Week 5 or premature termination) ]The MADRS comprises of 10 questions that are completed by the patient to determine their depression level. The MADRS was completed at Visit 2 (Baseline) prior to receiving the study drug and at Visit 4 (Week 5 or premature termination). Each item is scored on a 0-6 scale , where 0=no sadness to 6=extreme and continuous gloom and despondency, and the MADRS score is the sum of the 10 item scores (range 0-60). |
| **Notes** |  |

Risk of bias table

| **Bias** | **Authors' judgement** | **Support for judgement** |
| --- | --- | --- |
| Random sequence generation (selection bias) | Low risk | Patients were randomly assigned by computer using a block approach, first to 1 of 3 dose groups, and then within each group, to either active drug or placebo. |
| Allocation concealment (selection bias) | Unclear risk | The allocation to active drug or placebo was in a 3:1 ratio. The randomization was stratified by region (North America/Rest of the World). |
| Blinding of participants and personnel (performance bias) | Unclear risk | The procedure of blinding was insufficiently described. |
| Blinding of outcome assessment (detection bias) | Unclear risk | It was not mentioned if the outcome assessors in the trial were blinded or the extent of blinding was insufficiently described. |
| Incomplete outcome data (attrition bias) | High risk | The efficacy analyses were intent-to-treat. All patients who were Randomised and received at least 1 dose of study medication were entered into the analyses. Missing data for the efficacy endpoints were imputed using the last observation carry forward (LOCF) method. |
| Selective reporting (reporting bias) | Low risk | The protocol was published before and the outcomes specified in the protocol were reported on. |
| Other bias | Unclear risk | This study was funded by GW Pharmaceuticals and Otsuka. |

***Portenoy 2012 (4 - 10 sprays)***

| **Methods** | Randomised, doubleblind, placebo-controlled, parallel group, graded-dose design |
| --- | --- |
| **Participants** | **Inclusion criteria:**  1) Adult patients were eligible for the study if they had active cancer and chronic pain that was moderate or severe despite a stable opioid regimen that could not be made more effective by further opioid dose titration.  **Exclusion criteria:**  1) If they had a major psychiatric or cardiovascular disorder, epilepsy, or significant renal or hepatic impairment, or if they were pregnant, lactating or not using adequate contraception.  2) Patients who had received or who were due to receive therapies that were expected to change the pain (such as radiotherapy, or chemotherapy or hormonal therapy) also were excluded.  3) Patients who were currently using or had used marijuana, cannabinoidbased medications or rimonabant within 30 days of study entry, and were unwilling to abstain for the duration of the study, were excluded.  4) Patients receiving long-term methadone therapy for pain were not eligible because of concerns that its potency relative to other opioid agonists may vary with dose, rendering analyses using morphine equivalent milligrams less reliable. |
| **Interventions** | The study timeline included a 5- to 14-day baseline period, randomization, a 5-week titration and treatment period, and a poststudy visit after 2 weeks. The maximum duration was 9 weeks.  After randomization, patients entered a titration and treatment phase, which included a 1-week blinded dose titration period followed by 4 weeks of stable dosing.  Throughout this phase, patients self-administered the study drug (either nabiximols or an identicallyappearing placebo) as an oromucosal spray delivered using a pump.  Patients randomly assigned to Group 1 (low dose) were instructed to titrate the study medication to between 1 and 4 sprays per day. Those assigned to Group 2 (medium dose) titrated the number of sprays to between 6 and 10 sprays per day, and those assigned to Group 3 (high dose) titrated to between 11 and 16 sprays per day. |
| **Outcomes** | Primary Outcome Measures   1. Number of Patients With at Least 30% Improvement in Numerical Rating Scale (NRS) Average Pain Score From Baseline. Time Frame: 5 Weeks: Baseline (first 3 days) - Week 5 (last 3 days)   Secondary Outcome Measures   1. Change in Cumulative Average Pain Response Curves [ Time Frame: Baseline to end of treatment (Week 5) ]The cumulative response to treatment is the percentage changes from baseline in the mean NRS pain score as defined as the 30% response.The pain NRS was completed at the same time each day, i.e. bedtime in the evening. The patient was asked "on a scale of '0 to 10', please indicate the number that best describes your pain or average pain in the last 24 hours" 2. Change in Mean Daily NRS Pain Score (Average Pain). [ Time Frame: 5 Weeks: Baseline (first 3 days) - End of Treatment (last 3 days of week 5) ]The average pain NRS was complete at the same time each day, i.e. bedtime in the evening. The patient was asked "on a scale of '0 to 10', please indicate the number that best describes your pain or average pain in the last 24 hours" where 0 = no pain and 10 = pain as bad as you can imagine. 3. Change in Mean Daily NRS Pain Score (Worst Pain). [ Time Frame: 5 Weeks: Baseline (first 3 days) - End of Treatment (last 3 days of week 5) ]The worst pain NRS was completed at the same time each day, i.e. bedtime in the evening. The patient was asked "on a scale of '0 to 10', please indicate the number that best describes your worst pain in the last 24 hours" where 0 = no pain and 10 = pain as bad as you can imagine.. 4. Change in Sleep Disruption NRS [ Time Frame: 5 Weeks: Baseline - End of Treatment (Last 3 days of Week 5) ]The sleep disruption NRS was completed at the same time each day, i.e. bedtime in the evening. The patient was asked "on a scale of '0 to 10', please indicate how your pain disrupted your sleep last night?" where 0 = did not disrupt sleep and 10 = completely disrupted (unable to sleep at all). 5. Change in Brief Pain Inventory - Short Form (BPI-SF) [ Time Frame: Baseline (Visit 2) and End of Treatment (End of Week 5 or premature termination) ]The BPI-SF is a 14-item questionnaire that asks patients to rate pain over the prior week and the degree to which it interferes with activities on a 0 to 10 scale, where 0=no pain and 10=pain as bad as you can imagine. 6. Change in Patient Assessment of Constipation Quality of Life (PAC-QoL) [ Time Frame: Baseline (Visit 2) and End of Treatment (Week 5 or premature termination) ]The PAC-QoL questionnaire consists of 28 questions divided into the following areas: 4 questions on physical discomfort, 8 questions on psychosocial discomfort, 11 questions on worries/concerns and 5 questions on satisfaction. 7. Change in Patient Global Impression of Change - PGIC [ Time Frame: End of Week 5 ]A 7-point Likert-type scale was used, with the question: 'Please assess the status of your pain due to cancer since entry into the study using the scale below' with the markers "very much improved, much improved, slightly improved, no change, slightly worse, much worse or very much worse". 8. Change in Montgomery Asberg Depression Rating Scale (MADRS) [ Time Frame: Baseline and End of Treatment (Week 5 or premature termination) ]The MADRS comprises of 10 questions that are completed by the patient to determine their depression level. The MADRS was completed at Visit 2 (Baseline) prior to receiving the study drug and at Visit 4 (Week 5 or premature termination). Each item is scored on a 0-6 scale , where 0=no sadness to 6=extreme and continuous gloom and despondency, and the MADRS score is the sum of the 10 item scores (range 0-60). |
| **Notes** |  |

Risk of bias table

| **Bias** | **Authors' judgement** | **Support for judgement** |
| --- | --- | --- |
| Random sequence generation (selection bias) | Low risk | Patients were randomly assigned by computer using a block approach, first to 1 of 3 dose groups, and then within each group, to either active drug or placebo |
| Allocation concealment (selection bias) | Unclear risk | The allocation to active drug or placebo was in a 3:1 ratio. The randomization was stratified by region (North America/Rest of the World). |
| Blinding of participants and personnel (performance bias) | Unclear risk | The procedure of blinding was insufficiently described. |
| Blinding of outcome assessment (detection bias) | Unclear risk | It was not mentioned if the outcome assessors in the trial were blinded or the extent of blinding was insufficiently described. |
| Incomplete outcome data (attrition bias) | High risk | The efficacy analyses were intent-to-treat. All patients who were Randomised and received at least 1 dose of study medication were entered into the analyses. Missing data for the efficacy endpoints were imputed using the last observation carry forward (LOCF) method. |
| Selective reporting (reporting bias) | Low risk | The protocol was published before and the outcomes specified in the protocol were reported on. |
| Other bias | Unclear risk | This study was funded by GW Pharmaceuticals and Otsuka. |

***Riva 2018***

| **Methods** | A Fase II, Randomised, Double-Blind, Placebo-Controlled, Multicentre Study |
| --- | --- |
| **Participants** | Inclusion criteria:  Subjects must fulfil ALL of the following criteria:   - Written informed consent - Subject able and willing to comply with all study requirements - Affected by ALS, either of definite, probable or possible category according to the El Escorial revised criteria or by primary lateral sclerosis (Pringle's criteria) - Affected of spasticity, equal or above 1 in the Ashworth Scale for spasticity in 2 or more muscle groups - Who will judge spasticity a relevant cause of movements impairment - Subject has spasticity due to MND of at least three months duration, which is not wholly relieved with current anti-spasticity therapy - Subject fulfils at least one of the two criteria below. Subject must be either:Currently established on a regular dose of anti-spasticity therapy, orPreviously tried and failed, or could not tolerate suitable anti-spasticity therapy - Stabilization of factors affecting spasticity: any physiotherapy regimen or medication likely to affect spasticity will be optimised before the study and not altered in the 3 weeks before start of treatment - Subject is willing for his or her name to be notified to the responsible authorities for participation in this study, as applicable.   Additional inclusion Criteria to be met at baseline  • Subjects have registered spasticity NRS scores via the personal clinical diary over the 6 days (day 2 to day 7) before randomization  Exclusion criteria:   - Any concomitant disease or disorder that has spasticity-like symptoms or that may influence the subject's level of spasticity - Subjects receiving Botulinum Toxin during the preceding 6 months - Bedridden and tracheotomised patients - Fixed-tendon contractures - Severe cognitive impairment - Currently using or has used cannabis, cannabinoid-based medications or Acomplia (Rimonabant) within 30 days of study entry and unwilling to abstain for the duration of the study - Any history or immediate family history of schizophrenia, other psychotic illness, severe personality disorder or other significant psychiatric disorder other than depression associated with their underlying condition - Any known or suspected history of a diagnosed dependence disorder, current heavy alcohol consumption, current use of an illicit drug or current non-prescribed use of any prescription drug - Subjects with poorly controlled epilepsy or recurrent seizures (Subjects who have had one or more fits in the year prior to Visit 1 will be excluded) - Any known or suspected hypersensitivity to cannabinoids or any of the excipients - Subject has experienced myocardial infarction or clinically relevant cardiac dysfunction within the last 12 months or has a cardiac disorder that, in the opinion of the investigator would put the subject at risk of a clinically relevant arrhythmia or myocardial infarction - Subject has a diastolic blood pressure of <50 mmHg or >105 mmHg (when measured in a sitting position at rest for five minutes) or a postural drop in the systolic blood pressure of greater than 20 mmHg - Personal history suggestive of relevant impaired renal or hepatic function - Female subjects of child bearing potential, unless willing to ensure that they or their partner use effective contraception during the study and for three months thereafter - Female subject who is pregnant, lactating or planning pregnancy during the course of the study and for three months thereafter - Subjects who have received any IMP within the 8 weeks before Visit 1 - Any other significant disease or disorder which, in the opinion of the investigator, may either put the subject at risk because of participation in the study, or may influence the result of the study, or the subject's ability to participate in the study - Unwilling to abstain from donation of blood during the study - Patients will be asked not to drive while they will be receiving medication |
| **Interventions** | Experimental: Sativex  Cannabis Sativa extract Oromucosal spray, containing THC (27 mg/ml):CBD (25 mg/ml)  Placebo Comparator: Placebo  Placebo oromucosal spray |
| **Outcomes** | **Primary Outcome Measures**  Modified Ashworth scale (AS). [ Time Frame: Week 7 (6 weeks after randomization) ]Improvement in the modified 5 - points modified Ashworth scale (AS). The variable for analysis will be the change in AS from the baseline (visit 2, Week 2) to the end of treatment (visit 4, Week 7).  **Secondary Outcome Measures :**  Mean weekly spasticity, spasm frequency and sleep disruption Numeric Rating Scale (NRS) score [ Time Frame: Week 7 (6 weeks after randomizazion) ]  The variable for analysis will be the change in mean NRS from the baseline (days 0-7) to the last week of treatment (usually days 42-49). Proportion of subjects completing the study and showing an improvement of 30% or more and 50% or more in NRS from Baseline (Week 1) and end of study (last seven days of treatment)  **Other Outcome Measures:**  Pain NRS score [ Time Frame: Week 7 (6 weeks ater randomization) ]Pain will be measured with the mean of the last 3 days 0 - 10 daily pain intensity NRS.  Appetite increase [ Time Frame: Week 7 (6 weeks after randomization) ]Weight difference before and at the end of the study.  Function (Ten meters walk test, ALS-FRS, Barthel ADL Index) [ Time Frame: Week 7 (6 weeks aftar randomization) ]The time taken for the 10-metre walk, ALS-FRS and Barthel ADL Index will be analysed using the Visit 2 (week 1)result as baseline.  Global Impression of Change [ Time Frame: Week 7 (6 weeks after randomization) ]Carer Global Impression of Change and ease of transfer Physician Global Impression of Change Subject Global Impression of Change  Safety [ Time Frame: Week 4, week 7 ]Adverse events, Vital Signs, Physical Examination , oral examination |
| **Notes** |  |

Risk of bias table

| **Bias** | **Authors' judgement** | **Support for judgement** |
| --- | --- | --- |
| Random sequence generation (selection bias) | Low risk | A week after a baseline assessment, consecutive patients fulfilling the eligibility criteria were randomly assigned (1:1) to either active treatment or placebo. Randomisation was done centrally according to an allocation schedule with bal anced randomly permuted blocks of four via a computer­based algorithm to a consecutive series of numbers by an in dependent statistician, who had no other role in the study or in data analysis. |
| Allocation concealment (selection bias) | Low risk | Participants were assigned to groups consecutively by study investigators acccording to the allocation schedule. The treatment allocation code was kept in a sealed opaque envelope. |
| Blinding of participants and personnel (performance bias) | Low risk | All participants, in vestigators, site personnel, steering committee mem bers, and the study statistician were masked to the treat ment allocation. Vials and boxes were prepared centrally (GW Pharma, Cambridge, UK) and had standardised labelling to ensure maintenance of blinding |
| Blinding of outcome assessment (detection bias) | Low risk | All participants, in vestigators, site personnel, steering committee mem bers, and the study statistician were masked to the treat ment allocation. Vials and boxes were prepared centrally (GW Pharma, Cambridge, UK) and had standardised labelling to ensure maintenance of blinding |
| Incomplete outcome data (attrition bias) | Low risk | Generally, the trial is judged as at a low risk of bias due to incomplete outcome data if dropouts are less than 5% |
| Selective reporting (reporting bias) | Low risk | This trial is registered with ClinicalTrials.gov, number NCT01776970 |
| Other bias | Low risk | Funding Italian Research Foundation for Amyotrophic Lateral Sclerosis |

***Rog 2005***

| **Methods** | 5-week (1-week run-in, 4-week treatment) randomised, double-blind, placebo-controlled, parallel group study |
| --- | --- |
| **Participants** | **Inclusion criteria:**  Inclusion Central pain for which a nociceptive cause appeared unlikely was required to be of at least 3 months’ duration and expected to remain otherwise stable during the study.  **Exclusion criteria:**  1) Patients were ineligible if they had a history of major psychiatric disorder other than depression associated with their underlying condition; severe concomitant illness, seizures, history or suspicion of substance abuse; concomitant severe nonneuropathic pain or the presence of illness such as diabetes mellitus that could cause peripheral neuropathic pain; or scheduled procedures requiring general anesthesia during the study.  2) Patients were also excluded if they were pregnant, lactating, taking levodopa therapy within 7 days of study entry or had known or suspected hypersensitivity to cannabinoids. |
| **Interventions** | On the first day of treatment, up to four sprays were delivered in 2 hours and any signs of intoxication observed over 4 hours by the investigator and recorded by the participant on a 100-mm visual analogue scale (VAS) (0 no intoxication and 100 extreme intoxication). If the participant scored 25 mm on a predose VAS or the investigator had concerns, a dose could be omitted.  Participants who satisfactorily completed initial dosing were given written instructions to begin home dose titration the following day. No specific target dose was set, and the patients were advised to increase the number of sprays stepwise on consecutive days up to 48 sprays (THC 129.6 mg:CBD 120 mg) in 24 hours.  During telephone follow-up, patients were advised to optimize dosing when suboptimal benefit had been achieved. Those patients who satisfactorily completed the trial were offered the opportunity to participate in an open-label extension study, which will be reported separately.  For the NRS-11 scales, the mean score of the 7 days before the first dose was given served as baseline and the mean score of the last 7 (3 in the event of withdrawal) days before the final intake of test medication was used as final score. |
| **Outcomes** | Primary Outcome Measures:  1)Change From Baseline in the Mean Pain 0-10 Numerical Rating Scale Score at the End of Treatment (4 Weeks) [ Time Frame: 0 - 4 weeks ]  The average pain Numerical Rating Scale was completed at the same time each day, i.e. bedtime in the evening. The patient was asked "on a scale of '0 to 10', please indicate the number that best describes your pain or average pain in the last 24 hours" where 0 = no pain and 10 = pain as bad as you can imagine. A negative value indicates an improvement in pain score from baseline.  Secondary Outcome Measures:  1) Change From Baseline in the Mean 0-10 Numerical Rating Scale Sleep Score at the End of Treatment (4 Weeks)  2) Subject Global Impression of Change at Week 4. A 7-point Likert-type scale was used, with the question: 'Please assess the improvement in overall condition since the start of the study using the scale below' with the markers "very much improved, much improved, slightly improved, no change, slightly worse, much worse or very much worse".  3) Change From Baseline in the Mean Neuropathic Pain Scale 0-10 Numerical Rating Scale Score at the End of Treatment (Week 4)  4) Change From Baseline in the Mean Brief Repeatable Battery of Neuropsychological Test Score for 'Selective Reminding' at the End of Treatment (Week 4)  5) Change From Baseline in the Mean Brief Repeatable Battery of Neuropsychological Test Score for '10/36 Spatial Recall' at the End of Treatment (Week 4)  6) Change From Baseline in the Mean Brief Repeatable Battery of Neuropsychological Test Score for 'Symbol Digit Modalities' at the End of Treatment (Week 4)  7)Change From Baseline in the Mean Brief Repeatable Battery of Neuropsychological Test Score for the 'Paced Auditory Serial Addition Task' (PASAT) at the End of Treatment (Week 4)  8) Change From Baseline in the Mean Brief Repeatable Battery of Neuropsychological Test Score for 'Word List Generation' at the End of Treatment (4 Weeks)  9) Change From Baseline in the Mean Total Guy's Neurological Disability Scale Score at the End of Treatment (4 Weeks)  10) Change From Baseline in the Mean 0-100 mm Visual Analogue Scale Score for Intoxication Levels at the End of Treatment (4 Weeks)  11) Change From Baseline in The Hospital Anxiety and Depression Scale Score for Depression at the End of Treatment (4 Weeks)  12) Change From Baseline in The Hospital Anxiety and Depression Scale Score for Anxiety at the End of Treatment (4 Weeks)  13) Change From Baseline in the Multiple Sclerosis Functional Composite Score at the End of Treatment (4 Weeks)  14) Incidence of Adverse Events as a Measure of Patient Safety |
| **Notes** |  |

Risk of bias table

| **Bias** | **Authors' judgement** | **Support for judgement** |
| --- | --- | --- |
| Random sequence generation (selection bias) | Low risk | Patients were Randomised using a predetermined randomization code drawn up by a statistician who remained unknown to study personnel throughout the duration of the trial. |
| Allocation concealment (selection bias) | Low risk | Treatment allocation was made using Randomised permuted blocks of four (two active drug, two placebo). The identity of study medication assigned to patients, to which all study personnel remained blinded, was contained in individually sealed envelopes retained in the hospital 24-hour pharmacy and with the sponsor’s Pharmacovigilance Department. |
| Blinding of participants and personnel (performance bias) | Low risk | The participants and the treatment providers were blinded to intervention allocation and this was described. |
| Blinding of outcome assessment (detection bias) | Low risk | It was mentioned that outcome assessors were blinded and this was described. |
| Incomplete outcome data (attrition bias) | Low risk | The intention-to-treat (ITT) population was used for all efficacy analyses and was defined as all patients who entered the study, were Randomised, received at least one dose of study medication, and had on-treatment efficacy data. |
| Selective reporting (reporting bias) | Low risk | The protocol was published after the trial has begun, reporting pain assessment on VAS or NRS and serious adverse events. |
| Other bias | Unclear risk | GW Pharmaceuticals sponsored the trial, contributed to study design, provided trial medication and matching placebo and collected the data. GW Pharma Ltd. has contracted data handling and analysis to a contract research organization. The authors have received full access to the data and conducted an independent analysis. GW and Bayer Pharmaceuticals have had the opportunity to review the manuscript of the paper, but decision to publish rests with the authors. |

***Schimrigk 2017***

| **Methods** | Randomised, doubleblind, placebo-controlled parallel group design in 16 weeks followed by a 32 weeks open-label period |
| --- | --- |
| **Participants** | Inclusion Criteria:   - Diagnosis of multiple sclerosis acc. to McDonald diagnostic criteria - EDSS score between 3 and 8 (incl.) - Patients who are in a stable phase of multiple sclerosis - Patients with MS-related central neuropathic pain for at least 3 months   Exclusion Criteria:   - Severe concomitant diseases - Certain concomitant therapies (in particular: pain influencing concomitant therapies) - Dronabinol intake within the last 12 months prior to study entry or Marihuana use within one month prior to study entry |
| **Interventions** | Double-blind period started with a 4 weeks titration period to establish the patient-specific tolerable dose. Dosing was increased every 5 days by 2.5 mg to reach a daily dose between 7.5 and 15.0 mg. |
| **Outcomes** | Primary Outcome Measures:  1)Mean change of baseline pain severity score on the 11-point Likert Numerical Rating Scale recorded in patient diary [ Time Frame: max. 2.5 years ]  Secondary Outcome Measures:  1) Likert Numerical Rating Scale pain relief  2) Pain-related sleep interference  3) SF-36 (QoL-questionnaire)  4) Intake of rescue medication |
| **Notes** |  |

Risk of bias table

| **Bias** | **Authors' judgement** | **Support for judgement** |
| --- | --- | --- |
| Random sequence generation (selection bias) | Low risk | Patients were randomly allocated either to dronabinol or placebo in a 1: 1 ratio according to a computer-generated randomization code. Block packs with 4 kits were distributed to the sites and kits were allocated to patients in chronological order starting with the lowest available medication number at each site. |
| Allocation concealment (selection bias) | Low risk |  |
| Blinding of participants and personnel (performance bias) | Low risk | Blinding of patients, investigators and staff involved in the conduct of the study was maintained throughout the clinical trial. |
| Blinding of outcome assessment (detection bias) | Low risk | Blinding of patients, investigators and staff involved in the conduct of the study was maintained throughout the clinical trial. |
| Incomplete outcome data (attrition bias) | Low risk | The numbers and reasons for the withdrawals and dropouts for all outcomes were clearly stated and could be described as being similar to both groups |
| Selective reporting (reporting bias) | Low risk | Protocol was published before the trial was begun, and the outcomes specified in the protocol were reported on. |
| Other bias | Unclear risk | The present study was funded by Bionorica research GmbH (Innsbruck, Austria). |

***Selvarajah 2009***

| **Methods** | Randomised Placebo-Controlled Double-Blind Clinical Trial |
| --- | --- |
| **Participants** | Inclusion Criteria:   - Willing and able to give informed consent. - Male or female, aged 18 years or above. - Ability (in the investigators opinion) and willingness to comply with all study requirements. - Diagnosed with Type 1 or 2 diabetes mellitus as diagnosed according to the World Health Organisation (WHO) criteria. - Diagnosed with neuropathic pain due to distal symmetrical diabetic neuropathy of at least six months duration, as defined by a NDS score of at least 4, and in who pain is not wholly relieved with their current therapy. The NDS score must be attained from at least two different test parameters and not only the ankle jerk reflex. - The last six daily diary 0-10 NRS pain scores before randomisation summed to at least 24. - Stable dose of regular pain medication and non-pharmacological therapies (including TENS) for at least 14 days prior to the screening visit and willingness for these to be maintained throughout the study. - Agreement for the responsible authorities (as applicable in individual countries), their primary care physician, and their consultant, if appropriate, to be notified of their participation in the study.   Exclusion Criteria:   - Concomitant pain thought by the investigator to be of a nature or severity to interfere with their assessment of their painful diabetic neuropathy. - Uncontrolled diabetes with HbA1c blood levels of more than 11% at Visit1, Day B1. - Receiving a prohibited medication and were unwilling to stop or comply for the duration of the study. - Has used cannabinoid based medications within 60 days of study entry and were unwilling to abstain for the duration for the study. - Has used cannabis within 30 days of study entry and were unwilling to abstain for the duration for the study. - History of schizophrenia, other psychotic illness, severe personality disorder or other significant psychiatric disorder other than depression associated with their underlying condition. - Known or suspected history of alcohol or substance abuse. - History of epilepsy or recurrent seizures. - Known or suspected hypersensitivity to cannabinoids or any of the excipients of the IMP. - Postural drop of 20mmHg or more in systolic blood pressure at screening. - Medical history of gastroparesis. - Evidence of cardiomyopathy. - Experienced myocardial infarction or clinically relevant cardiac dysfunction within the last 12 months or had a cardiac disorder that, in the opinion of the investigator would put the subject at risk of a clinically relevant arrhythmia or myocardial infarction. - QT interval; of > 450 ms (males) or > 470 ms (females) at Visit 1. - Secondary or tertiary AV block or sinus bradycardia (HR <50bpm) or sinus tachycardia (HR>110bpm) at Visit 1. - Diastolic blood pressure of <50 mmHg or >105 mmHg in a sitting position at rest for five minutes prior to randomisation. - Impaired renal function i.e., creatinine clearance is lower than 50 ml/min at Visit 1. - Significantly impaired hepatic function, at Visit 1, in the investigator's opinion. - Female subjects of child bearing potential and male subjects whose partner was of child bearing potential, unless they were willing to ensure that they or their partner used effective contraception during the study and for three months thereafter. - If female, were pregnant or lactating, or were planning pregnancy during the course of the study and for three months thereafter. - Received an IMP within the 12 weeks before Visit 1. - Any other significant disease or disorder which, in the opinion of the investigator, may either put the subject at risk because of participation in the study, may influence the result of the study, or the subject's ability to participate in the study. - Following a physical exam, the subject had any abnormalities that, in the opinion of the investigator, would prevent the subject from safely participating in the study. - Intention to donate blood during the study. - Intention to travel internationally during the study. - Previous randomisation into this study. |
| **Interventions** | This was a 15 week (one week baseline and fourteen weeks treatment period), multicentre, double blind, randomised, placebo controlled, parallel group study to evaluate the efficacy of Sativex in subjects with pain due to diabetic neuropathy. Subjects were screened to determine eligibility and completed a seven-day baseline period. Subjects then returned to the centre for assessment, randomisation and dose introduction. Visits occurred at the end of weeks two, six, ten and at the end of the study (treatment week 14) or earlier if they withdrew. A follow up visit occurred 28 days after completion or withdrawal. Subjects in this study were given the opportunity to be enrolled in an open label extension study |
| **Outcomes** | Primary Outcome Measures:  1)The Change From Baseline in Mean Diabetic Neuropathy Pain 0-10 Numerical Rating Scale Score at the End of Treatment (Average of Last 7 Days Treatment)  2) Number of Responders at the 30% Improvement Level at the End of Treatment  Secondary Outcome Measures:  1) Change From Baseline in Mean Neuropathic Pain Scale Score at the End of Treatment  2) Change From Baseline in Mean Sleep Quality 0-10 Numerical Rating Scale Score at the End of Treatment  3) Subject Global Impression of Change at the End of Treatment  4) Change From Baseline in Mean Brief Pain Inventory (Short Form) 'Pain Severity Composite Score' at the End of Treatment  5) Change From Baseline in Mean Quality of Life EuroQol 5-D Weighted Health State Index Score at the End of Treatment Measured by Visual Analogue Scale  6) Change From Baseline in the Use of Rescue Analgesia at the End of Treatment  7) Incidence of Adverse Events as a Measure of Subject Safety  8) Change From Baseline in Mean Intoxication 0-10 Numerical Rating Scale Score at the End of Treatment |
| **Notes** |  |

Risk of bias table

| **Bias** | **Authors' judgement** | **Support for judgement** |
| --- | --- | --- |
| Random sequence generation (selection bias) | Unclear risk | The method of randomisation was not specified, but the trial was still presented as being randomised. |
| Allocation concealment (selection bias) | Unclear risk | The trial was classified as randomised but the allocation concealment process was not described. |
| Blinding of participants and personnel (performance bias) | Low risk | Masking: Quadruple (Participant, Care Provider, Investigator, Outcomes Assessor) |
| Blinding of outcome assessment (detection bias) | Low risk | Masking: Quadruple (Participant, Care Provider, Investigator, Outcomes Assessor) |
| Incomplete outcome data (attrition bias) | Low risk | An intent-to-treat analysis was undertaken |
| Selective reporting (reporting bias) | Low risk | The protocol was published before or at the time the trial was begun, and the outcomes specified in the protocol were reported on |
| Other bias | Unclear risk | Study sponsor: GW Pharmaceuticals Ltd. |

***Serpell 2014***

| **Methods** | A double-blind, randomised, placebo-controlled, parallel group study |
| --- | --- |
| **Participants** | **Inclusion criteria:**  1) Participants were aged 18 or older  2) Had mechanical allodynia within the territory of the affected nerve(s)  3) Participants had at least one of the following underlying conditions, which caused their PNP: post-herpetic neuralgia, peripheral neuropathy, focal nerve lesion, radiculopathy or Complex Regional Pain Syndrome (CRPS) type 2.  4) Participants also had a sum score of at least 24 on a pain 0–10 NRS for more than 6 days (baseline days 2–7) during the baseline period (average 0–10 NRS score of 4/10), and pain that was not wholly relieved by their current therapy.  5) In addition, their analgesic regimen was stable for at least 2 weeks preceding study entry and they were willing for the responsible authorities (i.e., primary care consultant or physician) to be notified of their participation in the study.  **Exclusion criteria:**  1) Participants with severe pain from other concomitant conditions were excluded, as were those with a history of significant psychiatric, renal, hepatic, cardiovascular or convulsive disorders, or with a known hypersensitivity to the study medication.  2) Those with CRPS type 1, cancer-related PNP or pain resulting from diabetes mellitus were excluded.  3) Participants receiving a prohibited medication [including cannabis or cannabinoid-based medications (in the last year), any analgesics taken on a ‘PRN’ (when required) basis, the introduction of any new analgesic medication, or any alteration to the dosage of the patient’s concomitant analgesic medication (other than the rescue analgesia provided), or all paracetamol-containing medications (stopped on the day the patient entered the baseline period)], who were unwilling to abstain for the study duration were also excluded.  4) Participants with a known history of alcohol or substance abuse.  5) Women of child-bearing potential or their partners were excluded unless willing to ensure effective contraception was used throughout the study, as were those who had received an investigational medicinal product within 12 weeks of screening.  6) Pregnant or lactating women and those planning a pregnancy were excluded.  7) Patients with any physical abnormality at screening (i.e., any abnormalities that, in the opinion of the investigator, would prevent the patient from safely participating in the study), or those intending to travel or donate blood during the study were also ineligible to take part. |
| **Interventions** | Following eligibility screening, patients completed a 7-day baseline period. Patients were then assessed, Randomised and received dose introduction.  Patients were Randomised to receive either THC/CBD spray or placebo.  Visits occurred at the end of weeks 2, 6, 10 and at the end of the study (treatment week 14) or earlier if they withdrew. A follow-up visit occurred 28 days after study completion or withdrawal. |
| **Outcomes** | Primary Outcome Measures:  1) Change From Baseline in Mean Peripheral Neuropathic Pain on a 0-10 Numerical Rating Scale (NRS) Score at the End of Treatment (15 Weeks)  Secondary Outcome Measures:  1) Change From Baseline in Neuropathic Pain Scale Score at the End of Treatment (15 Weeks)  2) Change From Baseline in Sleep Quality 0-10 Numerical Rating Scale Scores at the End of Treatment (15 Weeks)  3) Change in Baseline Mean Dynamic Allodynia Test Score at the End of Treatment (15 Weeks)  4) Change in Baseline Mean Punctate Allodynia Test Scores at the End of Treatment (15 Weeks)  5) Subject Global Impression of Change  6) Change From Baseline in Brief Pain Inventory (Short Form) Scores at the End of Treatment  7) Change From Baseline in Quality of Life EuroQol 5-D (Health Status Index) Score at the End of Treatment (15 Weeks)  8) Change From Baseline in Quality of Life EuroQol 5-D (Health Status Visual Analogue Scale) Score at the End of Treatment (15 Weeks)  9) Change From Baseline in the Use of Rescue Analgesia at the End Treatment (15 Weeks)  10) Incidence of Adverse Events as a Measure of Subject's Safety |
| **Notes** |  |

Risk of bias table

| **Bias** | **Authors' judgement** | **Support for judgement** |
| --- | --- | --- |
| Random sequence generation (selection bias) | Low risk | Randomization was carried out using a predetermined computer-generated randomization code |
| Allocation concealment (selection bias) | Low risk | Study medication was pre-packed by the GW Clinical Trial Supplies Department and dispatched to the investigator centres labelled with patient numbers. The randomization scheme involved patient numbers being assigned sequentially by the investigator staff. The investigator staff, pharmacy and GW Clinical Department held sealed code break envelopes for each patient. |
| Blinding of participants and personnel (performance bias) | Low risk | Participants, investigators are caregivers were all blinded to the treatment allocation. |
| Blinding of outcome assessment (detection bias) | Low risk | Participants, investigators are caregivers were all blinded to the treatment allocation. |
| Incomplete outcome data (attrition bias) | Low risk | All Randomised patients who received at least one dose of test treatment and had on-treatment efficacy data were included in the intention-to-treat (ITT) analysis set.  The per protocol (PP) analysis set included those with evaluable data for the primary parameter with no protocol deviations, which were considered to affect the comparison between treatments for this endpoint. |
| Selective reporting (reporting bias) | Low risk | The protocol was published after the trial has begun, reporting pain assessment on VAS or NRS and serious adverse events will grant the trial a grade of low risk of bias. |
| Other bias | Unclear risk | This study was sponsored by GW Pharma Ltd (GW). GW was involved in the study design, data collection and analysis, as well as in the preparation of this manuscript and publication decisions. |

***Skrabek 2008***

| **Methods** | Randomised, double-blind, placebo-controlled trial |
| --- | --- |
| **Participants** | **Inclusion criteria:**  1) Meeting The American College of Rheumatology (1990) criteria for the classification of fibromyalgia  2) Participants between 18 and 70 years of age  3) Having continued pain despite the use of other oral medications  4) No previous use of oral cannabinoids for pain management.  **Exclusion criteria:**  1) Pain was better explained by a diagnosis other than fibromyalgia  2) Abnormalities on routine baseline blood work including electrolytes, urea and creatinine, a complete blood count, and liver function tests  3) Heart disease 4) Schizophrenia or other psychotic disorder  5) Severe liver dysfunction  6) History of untreated nonpsychotic emotional disorders  7) Cognitive impairment  8) Major illness in another organ system  9) Pregnancy  10) Nursing mothers  11) A history of drug dependency  12) A known sensitivity to marijuana or other cannabinoid agents. |
| **Interventions** | Subjects met the eligibility criteria through a structured interview process, and participants provided informed consent.  Subjects were randomly assigned by the HSC pharmacy into treatment and control groups, each consisting of 20 participants.  Subjects in both groups were seen at baseline, after 2 weeks and 4 weeks of treatment and after a 4-week washout period. Subjects in the treatment group received 0.5 mg nabilone PO at bedtime for a 1-week period, with instructions to increase to 0.5 mg BID after 7 days. At the 2-week visit, subjects were evaluated for the presence of side effects and drug tolerance, and if they consented to continue, had the prescription increased to nabilone 0.5 mg PO in the morning and 1 mg PO at bedtime, with instructions to increase to 1 mg BID after 7 days. Subjects in the control group received a corresponding placebo. Subjects were assessed for the safety and efficacy of their prescription based on the outcome measures at the 2-, 4-, and 8-week follow-up visits. Subjects were asked to continue any current treatment for fibromyalgia, including breakthrough pain medications, but not to begin any new therapies. |
| **Outcomes** | Primary Outcome Measure:  1) Visual Analogue Scale Pain Scores  Secondary Outcome Measure:  1) Number of positive tender points  2) Average tender point pain threshold  3) The subject’s score on the FIQ (Fibromyalgia Impact Questionaire) |
| **Notes** |  |

Risk of bias table

| **Bias** | **Authors' judgement** | **Support for judgement** |
| --- | --- | --- |
| Random sequence generation (selection bias) | Unclear risk | Subjects were randomly assigned by the HSC pharmacy into treatment and control groups, each consisting of 20 participants |
| Allocation concealment (selection bias) | Unclear risk | The trial was classified as randomised but the allocation concealment process was not described |
| Blinding of participants and personnel (performance bias) | Low risk | The examining physicians and the subjects were blinded to the randomization process. All of the study medication was provided by Valeant Canada Limited (Montreal, Quebec, Canada) and was identical to placebo |
| Blinding of outcome assessment (detection bias) | Unclear risk | It was not mentioned if the outcome assessors in the trial were blinded or the extent of blinding was insufficiently described |
| Incomplete outcome data (attrition bias) | Unclear risk | If there was insufficient information to assess whether missing data were likely to induce bias on the results. |
| Selective reporting (reporting bias) | Low risk | A protocol was published before or at the time the trial was begun, and the outcomes specified in the protocol were reported on. |
| Other bias | Unclear risk | Supported by an unrestricted research grant provided by Valeant Canada Limited and an HSC Medical Staff Council Fellowship Fund. Valeant Canada Limited had no involvement in study design, interaction with patients, patient assessments, data analysis, or the authorship of this paper. |

***Stambaugh 1981***

| **Methods** | A four-way crossover, double-blinded trial |
| --- | --- |
| **Participants** | Patients with chronic moderate to severe pain from advanced cancer |
| **Interventions** | Experimental: Levonantradol (CP-50,556-I) intramuscular injection of either 0.5 mg or 2 mg  comparison: Placebo and 10 mg morphine |
| **Outcomes** | Analgesic effect measured on both verbal and visual analog scales  Toxicity from intramuscular injection. |
| **Notes** | Data extracted from an abstract. |

Risk of bias table

| **Bias** | **Authors' judgement** | **Support for judgement** |
| --- | --- | --- |
| Random sequence generation (selection bias) | Unclear risk | Not described |
| Allocation concealment (selection bias) | Unclear risk | Not described |
| Blinding of participants and personnel (performance bias) | Unclear risk | Not described |
| Blinding of outcome assessment (detection bias) | Unclear risk | Not described |
| Incomplete outcome data (attrition bias) | Unclear risk | Not described |
| Selective reporting (reporting bias) | Unclear risk | Not described |
| Other bias | Unclear risk | Not described |

***Staquet 1978***

| **Methods** | Randomised, double-blinded controlled trial |
| --- | --- |
| **Participants** | **Inclusion criteria:**  All the patients selected to participate in the study were hospitalized advanced cancer patients who reported continuous moderate to severe pain for at least 3 days at the time of admission to the study. Age ranged from 21 to 75.  **Exclusion criteria:**  1) Patients receiving large doses of narcotics  2) Patiens with insufficient mental clarity to judge discomfort or relief.  3) Patients with serious gastrointestinal pathology and with renal and hepatic diseases susceptible to interfere with drug metabolism or excretion. |
| **Interventions** | The study is a report of 2 consecutive crossover oral trials. In the first, 4 mg of NIB was compared with 50 mg of codeine and with placebo in thirty patients. In the second trial, in 15 patients, 50 mg secobarbital was compared with 4 mg of NIB and with placebo.  Each patient received the three medications, one on each of three successive days, in order randomly determined for each patient. |
| **Outcomes** | Not stated in the article. |
| **Notes** |  |

Risk of bias table

| **Bias** | **Authors' judgement** | **Support for judgement** |
| --- | --- | --- |
| Random sequence generation (selection bias) | Unclear risk | Not mentioned |
| Allocation concealment (selection bias) | Low risk | All the drugs were in identical capsules containing the same volume of powder, with lactose as a filler. A sealed envelope with the codes was kept in the ward |
| Blinding of participants and personnel (performance bias) | Unclear risk | Not mentioned |
| Blinding of outcome assessment (detection bias) | Unclear risk | Not mentioned |
| Incomplete outcome data (attrition bias) | Unclear risk | Not mentioned |
| Selective reporting (reporting bias) | Unclear risk | Not mentioned |
| Other bias | Unclear risk | Not mentioned |

***Svendsen 2004***

| **Methods** | Randomised double blind placebo controlled crossover trial. |
| --- | --- |
| **Participants** | **Inclusion criteria:**  Inclusion criteria were a diagnosis of multiple sclerosis (clinical definite multiple sclerosis and laboratory supported definite multiple sclerosis), age between 18 and 55 years, and central pain at the maximal pain site with a pain intensity score ≥3 on a 0-10 numerical rating scale.  **Exclusion criteria:**  We did not include patients with musculoskeletal disorders, peripheral neuropathic pain (caused by a lesion in nerve root, plexus, or peripheral nerve), or visceral pain at the maximal pain site.  Exclusion criteria were hypersensitivity to cannabinoids or sesame oil; heart disease; mania, depression, or schizophrenia; previous or present alcohol or drug misuse; treatment with tricylic antidepressants, anticholinergic agents, antihistamine, or central nervous system depressant drugs (with the exception of spasmolytic drugs); use of analgesic drugs except paracetamol; pregnancy or lactation; sexually active women without reliable contraception; patients unable to cooperate or complete the study; participation in other clinical trials within the previous month; use of marihuana within the three months before the study; and unwillingness to abstain from the use of marihuana during the entire period. |
| **Interventions** | At study entry we obtained a medical history and did a full neurological and physical examination, including electrocardiography. We allocated patients to treatment after a one week baseline period. We planned for the patients to receive three weeks’ (18-21 days’) treatment with dronabinol and three weeks’ (18-21 days’) treatment with placebo, with a washout period of at least 21 days between the treatment periods. Any analgesic drug (except paracetamol) was discontinued at least one week before the first visit.  During the baseline period and the two treatment periods the patients recorded pain intensity, use of escape medication, and adverse events in a diary. They recorded assessment of pain relief at the end of each treatment period. At the end of the baseline period and at the end of each treatment period we administered quantitative sensory tests, a health related quality of life questionnaire (SF-36), and the expanded disability status scale.  We administered dronabinol as 2.5 mg capsules and used dosage escalation. The initial dose was 2.5 mg daily, and the dose was increased by 2.5 mg every other day to a maximum dose of 5 mg (two capsules) twice daily. Placebo capsules were administered as identical looking capsules.  After 18-21 days of treatment we discontinued treatment without tapering. If adverse events occurred we reduced the dose to a minimum of 2.5 mg daily.  For the daily pain scale ratings we determined amedian value for the third week of treatment. We coded and summed the item scores in SF-36 and transformed them to a scale of 0 (poor health) to 100 (optimal health). |
| **Outcomes** | The predefined primary outcome measure was median spontaneous pain intensity in the last week of treatment.  Secondary outcome measures were median radiating pain intensity in the last week of treatment, pain relief, use of escape medication, patient preference, health related quality of life (SF-36), expanded disability status scale score, and quantitative sensory testing. |
| **Notes** |  |

Risk of bias table

| **Bias** | **Authors' judgement** | **Support for judgement** |
| --- | --- | --- |
| Random sequence generation (selection bias) | Low risk | We assigned patients to treatment sequence by using a computer generated randomisation code with a block size of six prepared by IPC-Nordic. |
| Allocation concealment (selection bias) | Low risk | Investigators allocated patients consecutively by time of inclusion at the study site. Sealed, completely opaque code envelopes containing information about treatment sequence for each patient were present at the study site. The code envelopes were returned unopened to the monitor after termination of the study. |
| Blinding of participants and personnel (performance bias) | Low risk | Both investigators and patients were blinded. |
| Blinding of outcome assessment (detection bias) | Low risk | Both investigators and patients were blinded |
| Incomplete outcome data (attrition bias) | Unclear risk | If a patient stopped the treatment prematurely we used the last week of treatment in the analysis according to the principle of intention to treat |
| Selective reporting (reporting bias) | Low risk | If there is no protocol or the protocol was published after the trial has begun, reporting pain assessment on VAS or NRS and serious adverse events will grant the trial a grade of low risk of bias. |
| Other bias | Low risk | The study was supported by grants from the Danish Multiple Sclerosis Society (grant no 2002/71045), grant 900035 from manager Ejnar Jonasseon and his wife’s memorial grant, and the Warwara Larsen Foundation (grant no 664.28), Denmark. |

***Toth 2012***

| **Methods** | An enriched-enrolment, randomised withdrawal, flexible-dose, double-blind, placebo-controlled, parallel trial |
| --- | --- |
| **Participants** | Inclusion Criteria   - Male or female subjects, ages between 18-80 years; - Signed and dated informed consent; - Females of childbearing potential must have a negative serum β-HCG pregnancy test and be practicing an effective form of contraception (accepted methods are hormonal [oral contraceptive or injectable contraceptive], double barrier with spermicide, or intrauterine device-IUD). Complete abstinence may be considered acceptable, but must be determined on a case-by-case basis with the clinical investigator. - Diagnosis of DPN-associated NeP syndrome, confirmed by a qualified Neurologist or pain specialist, with persistence for a minimum of 3 months. - Score of ≥4 on the DN4 questionnaire, a single page survey consisting of historical questions and one examination portion using light touch and pinprick over the region of suspected neuropathic pain. This has high sensitivity and specificity for neuropathic pain. - Must complete ≥4 daily pain diaries during the week of the screening phase prior to randomization; - Must have a daily mean pain score of ≥4 over the screening period prior to randomization based on Daily Pain Rating Scale (DPRS); - Must have a score of >40 mm on the visual analog scale (VAS) of the Short Form McGill Pain Questionnaire (SF-MPQ); - Screening laboratory values must be within normal limits, or abnormalities must be deemed clinically insignificant in the judgment of the investigator - Subject must be deemed capable of complying with study schedule, procedures and medications.   Exclusion Criteria   - Pregnant or lactating women or women of childbearing potential not using acceptable method of contraception; - Subjects with neuropathic pain that is not due to DPN - Any skin conditions in the affected areas with NeP that (in the judgment of the investigator) could interfere with evaluation of the NeP - Current or past DSM-IV-TRTM (2000) diagnosis of schizophrenia, psychotic disorder, bipolar affective disorder or obsessive-compulsive disorder and Major Depressive Disorder (MDD); - Current or past DSM-IV-TRTM (2000) diagnosis of substance abuse or dependence within the last 6 month; - Use of marijuana or other cannabinoids during the study. Discontinuation of these substances 30 days prior to the screening visit is permitted. The study consent must be signed and dated prior to the discontinuation of these substances; - Clinically significant or unstable conditions that, in the opinion of the investigator, would compromise participation in the study. This includes, for example, medical conditions such as, but not limited to: hepatic, renal, respiratory, hematological, immunologic, or cardiovascular diseases (eg, myocardial infarction within previous month, ventricular arrhythmia recent severe heart insufficiency), inflammatory or rheumatologic disease, active infections, symptomatic peripheral vascular disease, and untreated endocrine disorders; - History of seizure disorder, except febrile seizures of childhood; - A glycated hemoglobin (HbA1C) of more than 11% at screening - Any other condition, which in the investigator's judgment might increase the risk to the subject or decrease the chance of obtaining satisfactory data to achieve the objectives of the study. This includes any condition precluding nabilone use; - Malignancy within past 2 years with exception of basal cell carcinoma; - Urine screen positive for illicit substances, including tetrahydrocannaboids (THC) such as marijuana at screening (Visit 1); - Liver function tests or liver enzymes >3 times the upper limit of normal (ULN) - Other blood or urine laboratory results which are sufficiently abnormal in the view of the investigator(s) to raise concern about the enrollment of this subject in this study. - A previous history of intolerance or hypersensitivity to cannabinoids or other medications or substances with similar chemical structure; - Anticipated need for surgery during the study or within 4 weeks of completion; - Anticipated need for general anesthetics during the course of the study; - Anticipated need for hospitalization for any reason during the course of the study or within 4 weeks of completion; - Previous prescribed use of nabilone or other cannabinoids, including use of sample medications, within the 30 days prior to screening. Note that prior use of marijuana is not an exclusion criterion. - Participation in any other studies involving investigational or marketed products, concomitantly or within 30 days prior to entry in the study and/or - Employees or relatives of employees of the investigational site or Valeant Canada |
| **Interventions** | This was a single-center, parallel-group, double-blind, placebocontrolled randomised clinical trial with a 4-week single-blind flexible-dose phase of nabilone use followed by a randomised double- blind maintenance phase. It was designed to compare 5 weeks of stable-dose nabilone 1-4 mg/day with placebo, taken as 2 daily doses 12 hours apart with 1:1 allocation.  During the single-blind phase, subjects were initially prescribed nabilone 0.5 mg twice daily for 1 week, with weekly follow-up visits to determine efficacy, tolerability, and dosing adjustments. Nabilone was titrated up to 1.0 mg twice daily and as high as 2.0 mg twice daily as tolerated over the subsequent 3 weeks until day 28 of the study.  In order to be Randomised at day 28, subjects were required to have completed at least 75% of their daily pain diaries over the 4 weeks. If subjects did not achieve >30% pain relief for daily pain scores for the 7 days prior to day 28 when compared to the baseline scores, or if subjects developed intolerable side effects, then these subjects were not Randomised in the double-blind phase of the study.  During the double-blind phase, eligible subjects were randomly assigned to continue nabilone at the effective dose achieved in the flexible-dose single-blind phase or to receive placebo. For subjects receiving nabilone during the double- blind phase, the dose of nabilone achieved at day 28 was continued without change for 5 weeks. For subjects Randomised to placebo, there was a 1-week blinded taper of either 0.5 mg daily or twice daily of nabilone regardless of dose achieved at day 28, followed by strict placebo use over the subsequent 4 weeks. |
| **Outcomes** | Primary Outcome Measures:  1) To evaluate the efficacy of nabilone compared to placebo in the treatment of diabetic neuropathy-associated peripheral neuropathic pain (DPN).  Secondary Outcome Measures:  1) To evaluate safety and tolerability of nabilone for the treatment of neuropathic pain in subjects with diabetic peripheral neuropathy. |
| **Notes** |  |

Risk of bias table

| **Bias** | **Authors' judgement** | **Support for judgement** |
| --- | --- | --- |
| Random sequence generation (selection bias) | Low risk | An electronic randomization system was used to randomize individual subjects without block randomization as developed by an outside coordinator. |
| Allocation concealment (selection bias) | Low risk | Randomization was concealed from subjects, clinical coordinator, and assessing physicians. Medication was blinded for placebo using capsules of identical size, color, taste, and smell. |
| Blinding of participants and personnel (performance bias) | Low risk | Randomization was concealed from subjects, clinical coordinator, and assessing physicians. |
| Blinding of outcome assessment (detection bias) | Low risk | Randomization was concealed from subjects, clinical coordinator, and assessing physicians. |
| Incomplete outcome data (attrition bias) | Low risk | All analyses were based on the intention-to-treat population |
| Selective reporting (reporting bias) | Unclear risk | If no protocol was published and the outcome pain assessment on VAS or NRS and serious adverse events were not reported on. |
| Other bias | Unclear risk | This trial was funded by an unrestricted grant from Valeant Canada. Valeant Canada (Pharmaceuticals) played no role in the design or conduct of the study, and was not involved in the data analysis or construction of this manuscript. |

***Turcott 2018***

| **Methods** | Randomised, double-blind, parallel-group clinical trial |
| --- | --- |
| **Participants** | Inclusion Criteria:   - Non-small cell lung cancer (NSCLC) patients with unresectable stage IIIB/IV - ECOG performance status ≤2 - Life expectancy of > 4 months at time of screening - If woman of childbearing potential or a fertile man, he/she must agree to use an effective form of contraception during the study and for 30 days following the last dose of study drug (an effective form of contraception is abstinence, a hormonal contraceptive, or a double-barrier method) - Must be willing and able to give signed informed consent and, in the opinion of the Investigator, to comply with the protocol tests and procedures   Exclusion Criteria:   - Known allergy to some derivative of marijuana, there is a dependency or who have previously been treated with cannabinoids. - Consumption of dietary supplements at baseline. - Currently taking prescription medications intended to increase appetite or treat weight loss; these include, but are not limited to, testosterone, androgenic compounds, megestrol acetate, methylphenidate, and Anamorelin. |
| **Interventions** | After baseline assessment, patients were randomised by the protocol coordinator in a double-blind manner to receive capsules for oral administration of 0.5 mg daily of nabilone or placebo for 2 weeks, as administration of this agent must initiate with an induction dose as per regulatory indication. Subsequently the dose was increased to 1 mg daily for the next 6 weeks. Patients were evaluated at the time of inclusion, and 4 and 8 weeks after randomisation. |
| **Outcomes** | Primary Outcome Measures:  1) Anorexia. Lack of desire to eat food. Will be obtained through a questionnaire Anorexia / Cachexia scale from Functional Assessment of Anorexia Cachexia Therapy (FAACT) (score ≤24 diagnosis of anorexia) 2) Percentage weight loss. Percentage weight loss in the last month 3) Body Mass Index. It is an index of a person's weight in relation to height 4) Subjective Global Assessment. Convenient, fast and cheaper method used to make a nutritional assessment, consisting of 3 parts: anamnesis, physical examination and qualification. 5) Energy consumption. Total calories consumed on average per day for a subject  Secondary Outcome Measures:  1) Protein consumption.Total protein grams consumed on average per day for a subject 2) Lipids consumption.Total lipids grams consumed on average per day for a subject 3) Carbohydrate consumption. Total carbohydrate grams consumed on average per day for a subject 4) Nausea. adverse effect from Common terminology criteria for adverse event 5) Vomiting. Adverse effect from Common terminology criteria for adverse event 6) Constipation. Adverse effect from Common terminology criteria for adverse event 7) Diarrhea. Adverse effect from Common terminology criteria for adverse event 8) Dysgeusia. Adverse effect from Common terminology criteria for adverse event 9) Global Status of Quality of Life. The Global status of Quality of Life evaluation is evaluated using the validated Mexican-Spanish version of the European Organization for the Research and Treatment of Cancer Quality of Life Questionnaires specific for cancer with the items 29 and 30. 10) Physical functioning. The Physical functioning evaluation is evaluated from the validated Mexican-Spanish version of the European Organization for the Research and Treatment of Cancer Quality of Life Questionnaires specific for cancer 11) Role Functioning. The Role functioning evaluation is evaluated from the validated Mexican-Spanish version of the European Organization for the Research and Treatment of Cancer Quality of Life Questionnaires specific for cancer 12) Emotional Functioning. The Emotional Functioning evaluation is evaluated from the validated Mexican-Spanish version of the European Organization for the Research and Treatment of Cancer Quality of Life Questionnaires specific for cancer 13) Social Functioning. The Social Functioning evaluation is evaluated from the validated Mexican-Spanish version of the European Organization for the Research and Treatment of Cancer Quality of Life Questionnaires specific for cancer 14) Nausea/Vomiting. The Nausea/Vomiting evaluation is evaluated from the validated Mexican-Spanish version of the European Organization for the Research and Treatment of Cancer Quality of Life Questionnaires specific for cancer 15) Fatigue. The Fatigue evaluation is evaluated from the validated Mexican-Spanish version of the European Organization for the Research and Treatment of Cancer Quality of Life Questionnaires specific for cancer 16) appetite loss. The appetite loss evaluation is evaluated from the validated Mexican-Spanish version of the European Organization for the Research and Treatment of Cancer Quality of Life Questionnaires specific for cancer |
| **Notes** |  |

Risk of bias table

| **Bias** | **Authors' judgement** | **Support for judgement** |
| --- | --- | --- |
| Random sequence generation (selection bias) | Unclear risk | The method of randomisation was not specified, but the trial was still presented as being randomised. |
| Allocation concealment (selection bias) | Unclear risk | The trial was classified as randomised but the allocation concealment process was not described. |
| Blinding of participants and personnel (performance bias) | Unclear risk | The procedure of blinding was insufficiently described |
| Blinding of outcome assessment (detection bias) | Unclear risk | It was not mentioned if the outcome assessors in the trial were blinded or the extent of blinding was insufficiently described. |
| Incomplete outcome data (attrition bias) | Low risk | The numbers and reasons for the withdrawals and dropouts for all outcomes were clearly stated and could be described as being similar to both groups. |
| Selective reporting (reporting bias) | Unclear risk | Protocol was published after the study had begun, and the authors have not reported on serious adverse events |
| Other bias | High risk | Funding: Nabilone and placebo were donated by vealent pharmaceutical without any further participation in the trial. |

***Turcotte 2015***

| **Methods** | A single center, Randomised, double-blinded, parallel, placebo-controlled study |
| --- | --- |
| **Participants** | **Inclusion Criteria:**  1) Males and females between the ages of 18-65 years old with clinically definite RRMS  2) EDSS of < 6.5  3) Current treatment with gabapentin that is not effective at a stabilized dose of (>1800mg/day) for at least 1 month.  4) Visual Analogue Scale score for NPP symptoms > 5; pain present for at least 3 months  **Exclusion Criteria:**  1) Pregnancy or breastfeeding (Negative serum pregnancy test for all females of childbearing age; not currently breastfeeding)  2) History of alcohol or substance abuse (No history of alcohol or substance abuse)  3) Past or current nonpsychotic/psychotic emotional disorders (No history of non-psychotic emotional disorders)  4) Significant renal or hepatic insufficiency (No significant hepatic or renal insufficiency) 5) Cardiovascular disease (i.e., heart failure, cardiac arrhythmias) or uncontrolled hypertension (No significant cardiovascular disease or hypertension) 6) Hypersensitivity to nabilone or its derivatives (No known hypersensitivity and/or allergy to nabilone or its derivatives)  7) Current reported use of CBs and/or related products (No current use of cannabinoid or related products) 8) Patients with possible confounding causes of neuropathies (i.e., diabetes, HIV, etc.) |
| **Interventions** | This study was comprised of a prescreening visit (Visit 1), during which patients completed baseline pain assessment, followed by a 9-week (63-day) treatment phase (4-week dose “titration phase” and 5-week fixed dose “maintenance phase”). During the 9-week treatment phase, patients were required to attend two additional clinic visits at weeks 4 and 9 of the study (Visits 2 and 3, respectively). At the conclusion of the 9-week treatment, patients had the option of remaining on their current study medication (if on active treatment) or to be tapered off.  In order to minimize potential side effects, the dose of this medication was increased slowly over the course of the 4-week titration phase to a final dose of 1mg given twice a day, administered in the morning and at bedtime. |
| **Outcomes** | Primary Outcome Measures:  1) VAS   Secondary Outcome Measures:  1) SF MPQ  2) SF-36  3) PGIC |
| **Notes** |  |

Risk of bias table

| **Bias** | **Authors' judgement** | **Support for judgement** |
| --- | --- | --- |
| Random sequence generation (selection bias) | Low risk | Randomization assignments were generated using a preprogrammed computer software (Microsoft ExcelVR ) employing a random permuted blocks approach. |
| Allocation concealment (selection bias) | Low risk | In order to reduce the ability of investigators to predict subsequent treatment assignments of eligible patients, block sizes were also Randomised with either two or four patients per block. |
| Blinding of participants and personnel (performance bias) | Low risk | treating physicians were blinded to the treatment assignments for all participating patients. |
| Blinding of outcome assessment (detection bias) | Unclear risk | It was not mentioned if the outcome assessors in the trial were blinded or the extent of blinding was insufficiently described. |
| Incomplete outcome data (attrition bias) | Low risk | The trial is judged as at a low risk of bias due to incomplete outcome data if dropouts are less than 5%. |
| Selective reporting (reporting bias) | Low risk | Protocol was published before, and the outcomes specified in the protocol were reported on. |
| Other bias | Unclear risk | Valeant Canada (Pharmaceuticals) |

***Van de Donk 2018***

| **Methods** | Randomised, double-blind, placebo-controlled, 4-way crossover study |
| --- | --- |
| **Participants** | **Inclusion Criteria**:  Female fibromyalgia patients will be included if they have a pain score >5 for most of the day and meet the 2010 American College of Rheumatology diagnostic criteria.  **Exclusion Criteria**:  1) Unable to give written informed consent  2) Presence of medical disease that may alter PKPD of cannabinoids such as pulmonary, renal, liver, cardiac, gastro-intestinal, vascular disease  3) Allergy to study medication  4) Use of strong opioids  5) Use of benzodiazepines  6) History of illicit drug abuse (incl. cannabisrelated products) or alcohol abuse;  7) (Family) History of psychosis  8) Epilepsy;  9) Raised intracranial pressure;  10) Pregnancy and/or lactation;  11) The presence of pain syndromes other than fibromyalgia;  12) Age < 18 years. |
| **Interventions** | Patients will be Randomised to receive any of these 4 treatments: Bedrocan©, Bedrolite©,Bediol© or placebo. All four treatments will be administered by vaporizing using the Volcano Medic vaporizer. During and after inhalation multiple blood samples will be collected, to measure the pharmacokinetic effect. Electric and pressure pain tests will be used to measure the analgesic effect. Side-effects will be evaluated by two questionnaires (Bowdel and Bond&Lader)  Patients visited the research unit on 5 occasions. On their first visit, the patients were screened (medical history, physical examination, and urinalysis) and familiarized with the experimental setup (they were, for example, trained in the inhalation process). On each of their next visits, the patients received 1 of 4 possible cannabis treatments (in random order) with at least 2 weeks between visits. |
| **Outcomes** | Primary outcome:  PK data, spontaneous and experimental pain relief  Secondary outcome:  Mental state using Bowdle and Bond & Lader questionnaires |
| **Notes** |  |

Risk of bias table

| **Bias** | **Authors' judgement** | **Support for judgement** |
| --- | --- | --- |
| Random sequence generation (selection bias) | Low risk | Randomisation was performed by the pharmacy using a computergenerated randomization list. A distinct randomization sequence was created for each subject; randomization sequence was controlledwith just 2 subjectswith an identical treatment sequence |
| Allocation concealment (selection bias) | Low risk | On the day before the experiment, the subject was allocated to treatment by the pharmacy after receiving a fax message from the investigators with the participant’s identifier code and study visit number. Treatment was prepared on the day of the study and collected by a technician from the pharmacy in a closed opaque canister labeled with the patient’s identifier code and study visit number; the contents of the canister were emptied in the vaporizer. |
| Blinding of participants and personnel (performance bias) | Low risk | The investigators (and patients) remained blinded until data analysis was complete (June 2018). |
| Blinding of outcome assessment (detection bias) | Low risk | The investigators (and patients) remained blinded until data analysis was complete (June 2018). |
| Incomplete outcome data (attrition bias) | Low risk | The numbers and reasons for the withdrawals and dropouts for all outcomes were clearly stated and could be described as being similar to both groups. |
| Selective reporting (reporting bias) | Unclear risk | If a protocol was published before or at the time the trial was begun, and the outcomes specified in the protocol were reported on |
| Other bias | High risk | This investigator-initiated trial was performed in collaboration with Bedrocan International BV (Veendam, the Netherlands). Bedrocan International BV was responsible for the production and delivery of the cannabis products and the Volcano device for cannabis inhalation. M.A. Kowal is an employee of Bedrocan International BV, the Netherlands.  No funding stated. |

***vanAmerongen 2017***

| **Methods** | Randomised, double-blind, placebo- controlled, 2-way crossover design |
| --- | --- |
| **Participants** | **Inclusion criteria:**  Twenty-four patients 18 years or older with a diagnosis of progressive (primary or secondary) MS according to the revised McDonald criteria 15 who had a diseasedurationof 41 year and were clinically stable for at least 30 days before the start of the challenge phase were to be enrolled. Inaddition, patients had to have moderate spasticity as defined by an Ashworth score of >2 (range,0-4) and a Kurtzke Expanded Disability Status Scale score between 4.5 and 7.5 at baseline (range,0-10).  **Exclusion criteria:**  Current use of Δ9-THC was exclusionary |
| **Interventions** | Each of the 2 visits in the challenge phase consisted of up-titration of 3 consecutive drug administrations with a 100-minute interval in ascending order. If well tolerated, the 3 dose levels were predetermined to be 3, 5 and 8 mg, leading to a total daily dose of 16 mg.  Between the 2 visits was a washout period of 7 to 14 days.  On the basis of the findings of the challenge phase, patients start with a predetermined daily dose divided over 3 intakes. After 2 weeks of treatment, the dose for each patient was evaluated and increased when considered appropriate.  All randomised patients completed the challenge phase and were subsequently enrolled in the treatment phase. |
| **Outcomes** | The primary end point for the treatment phase was the H/M ratio.  Secondary end points were biomarkers for efficacy that were either measured in the clinic (Ashworth score, subjective spasticity [NRS], number of spasms and pain using an NRS and the McGill Pain Questionnaire) or measured at home using a daily diary (subjective spasticity [NRS], number of spasms and pain [NRS]). Furthermore, a set of functional outcome measures was selected to assess treatment effects: Expanded Disability Status Scale, the Patient’s Global Impression of Change, quality of sleep as determined by the Pittsburgh Sleep Quality Index, walking distance recorded by the Timed 25-Foot Walk Test, the Fatigue Severity Scale. |
| **Notes** |  |

Risk of bias table

| **Bias** | **Authors' judgement** | **Support for judgement** |
| --- | --- | --- |
| Random sequence generation (selection bias) | Unclear risk | The method of randomisation was not specified, but the trial was still presented as being randomised. |
| Allocation concealment (selection bias) | Unclear risk | The trial was classified as randomised but the allocation concealment process was not described. |
| Blinding of participants and personnel (performance bias) | Unclear risk | The procedure of blinding was insufficiently described. |
| Blinding of outcome assessment (detection bias) | Unclear risk | It was not mentioned if the outcome assessors in the trial were blinded or the extent of blinding was insufficiently described. |
| Incomplete outcome data (attrition bias) | Low risk | the trial is judged as at a low risk of bias due to incomplete outcome data if dropouts are less than 5%. However, the 5% cut-off is not definitive. |
| Selective reporting (reporting bias) | Low risk | If there is no protocol or the protocol was published after the trial has begun, reporting pain assessment on VAS or NRS and serious adverse events will grant the trial a grade of low risk of bias. |
| Other bias | Unclear risk | The present study was funded by Echo Pharmaceuticals |

***Vela 2021***

| **Methods** | The trial (NordCAN) was designed as a single-centre, double-blind, randomized and placebo-controlled trial conducted at the Rheumatological Research Unit at the Department of Rheumatology, Aalborg University Hospital, Denmark. |
| --- | --- |
| **Participants** | Inclusion criteria were fulfilling the 2006 Classification Criteria for Psoriatic Arthritis42 or the 1990 American College of Rheumatology criteria, pain intensity during the past 24 hours measured by a visual analogue scale (VAS) $30/100 mm at inclusion, age $18 years, and an ability and willingness to give written informed consent and to meet the requirements of the trial protocol.  Exclusion criteria included concurrent diagnosis of chronic regional pain syndrome or neuropathy, other known diseases where exacerbations needed to be treated with systemic corticosteroids (ie, certain types of inflammatory bowel disease) or patients who have received systemic corticosteroid treatment during the past 3 months, concurrent diagnosis of other inflammatory joint diseases, eg, rheumatoid arthritis or ankylosing spondylitis, and patients with gout if their disease had not been in remission for more than 6 months. Concurrent active malignant disease; planned major surgery during the intervention period or recent major surgery; current or planned pregnancy during the trial period; known allergy or contraindication to CBD treatment; previous addictive behaviour defined as abuse of cannabis, opioids, or other recreational or pharmaceutical drugs; severely decreased liver function; kidney function or known chronic heart failure; history of epilepsy; or severe cramps were also exclusion criteria. |
| **Interventions** | Patients initially received either oral CBD 10 mg or a placebo tablet once daily with the dose increased to 10 mg twice daily after 2 weeks. Patients were contacted by the investigator after 4 weeks, and those not experiencing a pain reduction of more than 20 mm on the VAS had their dose increased to 10 mg thrice daily until the end of treatment period. |
| **Outcomes** | The primary outcome was the difference between groups in the change of patient-reported pain intensity during the past 24 hours on VAS after 12 weeks of treatment (Difference VAS-pain).  Safety outcomes included percentage of patients experiencing adverse events (AEs) and a characterization of serious AEs (SAEs).  Explorative outcomes included assessment of anxiety and depression quantified using the Hospital Anxiety and Depression Scale (HADS)48 providing a score of 0 to 21 for each domain, where higher scores equal greater involvement of either anxiety or depression.  Subjective assessment of sleep quality was quantified using the Pittsburgh Sleep Quality Index (PSQI). Scores ranging from 0 to 21, where 21 indicates severe difficulties in all sleep-related areas |
| **Notes** |  |

Risk of bias table

| **Bias** | **Authors' judgement** | **Support for judgement** |
| --- | --- | --- |
| Random sequence generation (selection bias) | Low risk | The randomisation sequence was computer generated by a trial pharmacist from Glostrup Pharmacy, who had no clinical involvement in the trial |
| Allocation concealment (selection bias) | Low risk | Stratification was based on disease (PsA or Hand-OA), and allocation was concealed from patients and investigators. |
| Blinding of participants and personnel (performance bias) | Low risk | Double-blinded. The active synthetic CBD tablet and placebo tablet (containing the same ingredients except CBD) were produced by Glostrup Pharmacy (Glostrup, Denmark). |
| Blinding of outcome assessment (detection bias) | Unclear risk | Not described. |
| Incomplete outcome data (attrition bias) | Low risk | The efficacy analysis was based on the data from the full- analysis set, ie, the intention-to-treat population, including all participants who were randomised, assessed at baseline, and received at least one dose of trial medication.  The safety analysis includes all patients who were randomly assigned to a trial group and have had exposure to the trial drug or placebo. |
| Selective reporting (reporting bias) | Low risk | The NordCAN project was registered on ClinicalTrials.gov (NTC03693833) and in the European Clinical Trials database (2017-003574-13). The trial was continually monitored by the Good Clinical Practice (GCP) unit of Aalborg University Hospital, externally audited by the Danish Medicine Agency, and was conducted in accordance with the Declaration of Helsinki, GCP, and Danish regulatory requirements. |
| Other bias | Low risk | The trial appeared free of other types of bias. |

***Wade 2003***

| **Methods** | A consecutive series of double-blind, Randomised, placebo-controlled single-patient cross-over trials with two-week treatment periods. |
| --- | --- |
| **Participants** | Inclusion criteria:  Eligible patients had to have a neurological diagnosis and to be able to identify troublesome symptoms which were stable and unresponsive to standard treatments.  Exclusion criteria:  Patients were excluded if they had a history of: drug or alcohol abuse, serious psychiatric illness (excluding depression associated with the neurological condition), serious cardiovascular disease or active epilepsy. |
| **Interventions** | The test articles were whole-plant extracts of delta-9-tetrahydrocannabinol (THC-rich CME), cannabidiol (CBD-rich CME) and a 1:1 preparation of the two (THC:CBD). These were presented in a pump-action sublingual spray that delivered 2.5 mg THC and/or CBD at each actuation. The maximum permitted dose of each CME was 120 mg / 24 hours. The placebo spray contained inert plant material and solvent only. |
| **Outcomes** | The following measures were used. Subjects kept a daily diary in which they scored their target symptoms by means of visual analogue scales (VAS) at the same time each day. They also used VAS to provide a daily record of subjective intoxication, alertness, appetite, happiness, relaxation, optimism, energy, general well-being, sleep and feeling refreshed. |
| **Notes** |  |

Risk of bias table

| **Bias** | **Authors' judgement** | **Support for judgement** |
| --- | --- | --- |
| Random sequence generation (selection bias) | Low risk | Randomization was achieved using Williams’ squares, and vials for each two-week period were allocated and coded before despatch to the investigators. |
| Allocation concealment (selection bias) | Unclear risk | Not described |
| Blinding of participants and personnel (performance bias) | Unclear risk | Not described |
| Blinding of outcome assessment (detection bias) | Unclear risk | Not described |
| Incomplete outcome data (attrition bias) | Unclear risk | Not described clearly |
| Selective reporting (reporting bias) | Unclear risk | Not described |
| Other bias | Unclear risk | The study was funded by GW Pharmaceuticals plc (GWP) and supported by the Nuffield Orthopaedic Centre NHS Trust. |

***Wade 2004***

| **Methods** | Randomised,  placebo-controlled, double-blind, parallel group study |
| --- | --- |
| **Participants** | **Inclusion criteria:**  Have clinically confirmed MS of any type. This was undertaken through taking a history, undertaking a full examination, and full review of all hospital notes;  . have been stable over the preceding four weeks with no relapse, confirmed clinically on entry to the study;  . be on stable regular medication (i.e., not changed in the  last four weeks);  . be willing to abstain from alternative cannabinoid use  for seven days prior to screening and throughout the  study; and  . volunteer one of the five target symptoms at a sufficient  level of severity: spasticity, spasms, bladder problems, tremor or pain that was not obviously musculoskeletal.    **Exclusion criteria:**  1) Primary symptom was rated at less than 50% of maximal severity.  2) Current or past history of drug or alcohol abuse  3) Significant psychiatric illness other than depression associated with MS,  4) Serious cardiovascular disorder  5) Significant renal or hepatic impairment or history of epilepsy.  Patients could not be included if they had a planned visit abroad during the active study. |
| **Interventions** | Following the two-week base line period, patients returned to the study centre for review of eligibility, including further VAS completion for target symptoms. If the inclusion criteria were still fulfilled, dosing with Randomised medication was initiated.  After the six-week double-blind parallel group trial, patients returned to the study centre for a repeat of the full assessment battery.  All patients were then offered active medication for a further four weeks. Retitration was necessary because for the placebo group this would have been a patient’s first exposure to active medication  At the end of this second period, a total of ten weeks dosing, patients had the opportunity to continue into an open-label long-term safety and tolerability study. |
| **Outcomes** | **Primary measure outcomes:**  100 mm VAS for the primary target symptom, and for any other of the five target symptoms that were troublesome;  Barthel Activities of Daily Living (ADL) index, the Rivermead Mobility Index, the short Orientation- Memory-Concentration Test, the Adult Memory and Information Processing Battery test of attention adapted for patients with MS, the General Health Questionnaire, the Guy’s Neurological Disability scale (GNDS), the Beck Depression Inventory, the Fatigue Severity Scale and VAS to rate sleep: quality, amount and feelings on awakening;  . diary records using a VAS of spasm frequency, feeling of intoxication, and severity of each of the target symptoms on one nominated day each week; and, where appropriate:  . The modified Ashworth Scale of Spasticity measured at wrist, elbow, knee and ankle and summed across all joints (total score >20), a tremor ADL questionnaire, the Nine-Hole Peg Test of manual dexterity, a questionnaire on disability arising from urinary incontinence and the time in seconds to walk 10 m.  **Secondary measure outcomes:** |
| **Notes** |  |

Risk of bias table

| **Bias** | **Authors' judgement** | **Support for judgement** |
| --- | --- | --- |
| Random sequence generation (selection bias) | Low risk | Patients were Randomised by permuted blocks of size four, stratified by nominated primary symptom and centre |
| Allocation concealment (selection bias) | Low risk | The pharmacist at each centre was provided with a randomization scheme for each primary symptom and assigned the treatments in sequential patient number order from the appropriate randomization list. |
| Blinding of participants and personnel (performance bias) | Unclear risk | The procedure of blinding was insufficiently described. |
| Blinding of outcome assessment (detection bias) | Unclear risk | The extent of blinding was insufficiently described. |
| Incomplete outcome data (attrition bias) | Unclear risk | The numbers and reasons for the withdrawals and dropouts for all outcomes were clearly stated and could be described as being similar to both groups. |
| Selective reporting (reporting bias) | Unclear risk | No protocol was published and the outcome pain assessment on VAS or NRS and serious adverse events were not reported on. |
| Other bias | Unclear risk | Funding: GW Pharma Ltd. GW Pharma Ltd contributed to the study design and was involved in collection of the data. Data handling and analysis were contracted by GW Pharma Ltd to an independent research organization. |

***Wallace 2015***

| **Methods** | Randomised, double-blinded, placebo controlled crossover study |
| --- | --- |
| **Participants** | **Inclusion criteria:**  Participants were men and women  1) Age 18 or older with  2) Diabetes mellitus type 1 or type 2, who had stable glycemia (HbA1c < 11%) and were maintained by diet or a stable regimen of diabetic therapy for at least 12 weeks before the evaluation,  3) Presence of both spontaneous and evoked pain in the feet,  4) At least a six-month history of painful diabetic peripheral neuropathy diagnosed according to research diagnostic criteria (using the Michigan Neuropathy Screening Instrument), which included the presence of abnormal bilateral physical findings (reduced distal tendon reflexes, distal sensory loss) or electrophysiological abnormalities (distal leg sensory nerve conduction studies), plus paresthesiae and a spontaneous pain of intensity of > 4 on the 11-point Numeric Rating Scale.  **Exclusion criteria:**  1) Current DSM-IV substance use disorders;  2) Lifetime history of dependence on cannabis;  3) Lifetime history of DSM-IV schizophrenia, bipolar disorder, generalized anxiety or panic disorder, or previous psychosis with or intolerance to cannabinoids;  4) Current use of cannabis within the past 30 days; positive urine toxicology screen for cannabinoids during the wash-in week before initiating study treatment;  6) Pregnant or planning pregnancy; or positive urine pregnancy test at baseline;  7) Serious medical conditions that might affect participant safety or the conduct of the trial (e.g., cardiac or pulmonary disease);  8) Other medical conditions that are associated with peripheral neuropathy or pain of vascular origin that might confound the assessment of painful DPN;  9) Lower extremity amputations other than toes; and documented unstable blood glucose (fasting < 70mg/dl or random blood glucose >250mg/dl). If subjects were taking medications to treat the DPN pain, they were required to maintain a stable dose for 30 days prior and for the duration of the study |
| **Interventions** | Subjects participated in four sessions, separated by 2 weeks, where they were exposed to placebo, or to low (1% tetrahydrocannabinol, THC), medium (4% THC), or high (7% THC) dose of cannabis.  Baseline assessments of spontaneous pain, evoked pain and cognitive testing were performed. Subjects were then administered aerosolized cannabis or placebo and pain intensity and subjective “highness” scores was measured at 5, 15, 30, 45, and 60 minutes and then every 30 minutes for an additional 3 hours. Cognitive testing was performed at 5 and 30 minutes and then every 30 minutes for an additional 3 hours. |
| **Outcomes** | **Primary outcome measures:**  1) Spontaneous Pain Score  **Secondary outcome measures:**  1) Acute sensory threshold  2) Experimental pain score  3) Cognitive testing  4) Subjective highness  5) Adverse events |
| **Notes** |  |

Risk of bias table

| **Bias** | **Authors' judgement** | **Support for judgement** |
| --- | --- | --- |
| Random sequence generation (selection bias) | Unclear risk | Randomisation was performed by a research pharmacist using a random number permutations. |
| Allocation concealment (selection bias) | Unclear risk | The key to study assignment was withheld from investigators until completion statistical analyses. |
| Blinding of participants and personnel (performance bias) | Unclear risk | The procedure of blinding was insufficiently described. |
| Blinding of outcome assessment (detection bias) | Unclear risk | The extent of blinding was insufficiently described. |
| Incomplete outcome data (attrition bias) | Low risk | The trial is judged as at a low risk of bias due to incomplete outcome data if dropouts are less than 5% |
| Selective reporting (reporting bias) | Low risk | A protocol was published before or at the time the trial was begun, and the outcomes specified in the protocol were reported on |
| Other bias | Low risk | Supported by Grant C00-SD-107 from the University of California Center for Medicinal Cannabis Research, La Jolla, California, United States of America |

***Ware 2010***

| **Methods** | Randomised, double-blind, placebo-controlled, four-period crossover design |
| --- | --- |
| **Participants** | **Inclusion criteria:**  1) Men and women aged 18 years or older with neuropathic pain of at least three months in duration caused by trauma or surgery,  2) Allodynia or hyperalgesia, and with an average weekly pain intensity score greater than 4 on a 10-cm visual analogue scale.  3) Participants had a stable analgesic regimen and reported not having used cannabis during the year before the study  4) Potential participants had to have normal liver function (defined as aspartate aminogransferase less than three times normal), normal renal function (defined as a serum creatinine level < 133 μmol/L), normal hematocrit (> 38%) and a negative result on beta-human chorionic gonadotropin pregnancy test (if applicable).  5) Women of child-bearing potential consented to use adequate contraception during the study and for three months afterward.  **Exclusion criteria:**  1) Pain due to cancer or nociceptive causes,  2) Presence of significant cardiac or pulmonary disease,  3) Current substance abuse or dependence (including abuse of or dependence on cannabis), history of psychotic disorder, current suicidal ideation, pregnancy or breastfeeding,  4) Participation in another clinical trial within 30 days of enrolment in our trial, and ongoing insurance claims. |
| **Interventions** | Adults with post-traumatic or postsurgical neuropathic pain were randomly assigned to receive cannabis at four potencies (0%, 2.5%, 6% and 9.4% tetrahydrocannabinol) over four 14-day periods in a crossover trial. Each period was 14 days in duration, beginning with five days on the study drug followed by a nine-day washout period.  Participants were instructed to inhale for five seconds while the cannabis was lit, hold the smoke in their lungs for ten seconds, and then exhale. The beginning of inhalation was recorded as the onset of the exposure. |
| **Outcomes** | **Primary outcome measure:**  1) Average pain intensity measured daily during each cycle by 100 mm visual analogue scale (VAS) 2) No pain and worst pain possible will be used as anchors in the 11-item numerical rating scale. The daily pain intensity score will be averaged across all study days for each cycle to comprise the main outcome variable.  **Secondary outcome measures:**  The main clinical outcomes will be the subjective 'high', mood, quality of life, and quality of sleep. Treatment discernment will be assessed by potency assessments at inpatient and follow up visits: 1) Trial feasibility: practical issues of recruitment and compliance will be recorded during the study 2) Pain quality: the McGill Pain Questionnaire (MPQ) measures affective and cognitive aspects of pain experience at patient visits. This will be administered on day five of each of the four five-day treatment cycles. 3) Sleep: a modification of the Leeds Sleep Evaluation Questionnaire (LSEQ) will be used. This will be administered by daily telephone interviews during each treatment cycle. 4) Mood: the short form Profile of Mood States (POMS) will be administered on day five of each of the four five-day treatment cycles 5) Quality of life: quality of life will be assessed using the EuroQOL 5D instrument, which will be administered on day five of each of the four five-day treatment cycles 6) Physiological assessments: physiological measurements (blood pressure, heart rate, electrocardiogram [ECG] changes, pulse oximetry, respiratory rate) will be conducted at 15 minute intervals during the first hour and hourly for two hours after smoking on day one of each treatment cycle 7) Quantitative sensory testing (QST): QST of cutaneous thermal sensitivity will be performed at baseline (during the screening visit) and on day five of each of the four five-day treatment cycles 8) 'High': will be measured subjectively at 15 minute intervals during the first hour and hourly for two hours after smoking on day one of each treatment cycle using an unmarked VAS scale (anchors: 0 is not at all, 10 is extremely) 9) Other subjective effects: the subjects will be asked to identify their current level of 'relaxed', 'stressed', 'happy' measured subjectively at 15 minute intervals during the first hour and hourly for two hours after smoking on day one of each treatment cycle using 100 mm VAS scales using the anchors 'not at all' and 'extremely' 10) Potency assessments: the patients’ ability to detect the strength of each cannabis preparation administered will be tested using subjective potency assessments. This will be administered on day one and day five of each treatment cycle |
| **Notes** |  |

Risk of bias table

| **Bias** | **Authors' judgement** | **Support for judgement** |
| --- | --- | --- |
| Random sequence generation (selection bias) | Low risk | Eligible participants were Randomised to a sequence of treatment periods based on a Latin square design. |
| Allocation concealment (selection bias) | Unclear risk | Not mentioned |
| Blinding of participants and personnel (performance bias) | Low risk | A panel of nine independent personnel examined the appearance of the four cannabis preparations and found no association between estimated and true potency (data not shown). |
| Blinding of outcome assessment (detection bias) | Low risk | harmacist. A panel of nine independent personnel examined the appearance of the four cannabis preparations and found no association between estimated and true potency (data not shown). |
| Incomplete outcome data (attrition bias) | Unclear risk | Not mentioned |
| Selective reporting (reporting bias) | Low risk | Reporting pain assessment on VAS or NRS and serious adverse events will grant the trial a grade of low risk of bias. |
| Other bias | Low risk | This trial was supported by the Canadian Institutes of Health Research (grant no. JHM-50014). Financial support for pharmacokinetic assays was received from the Louise and Alan Edwards Foundation. |

***Wisley 2008***

| **Methods** | Randomised, double-blinded, placebo- controlled, crossover design, using high-dose cannabis (7% delta-9-THC), low-dose cannabis (3.5% delta-9- THC), and placebo cigarettes. |
| --- | --- |
| **Participants** | **Inclusion criteria:**  **Exclusion criteria:**  If the criteria for severe major depressive disorder were met, the candidate was excluded from participation.  Because the effects of cannabis can exacerbate mental ill- ness24,54 and have been linked to an increase in the risk of suicide,22 candidates with a history or diagnosis of schizo- phrenia or bipolar depression were also excluded. Medical illnesses were also evaluated, and exclusion criteria in- cluded uncontrolled hypertension, cardiovascular disease, chronic pulmonary disease (eg, asthma, chronic obstructive pulmonary disease), and active substance abuse. |
| **Interventions** | Participants were recruited from the UCDMC and VANCHCS pain clinics through initial contact by providers intimately involved in the patient’s care as well as newspaper advertisements and postings in newsletters.  Qualified candidates with complex regional pain syndrome (CRPS type I), spinal cord injury, periph- eral neuropathy, or nerve injury were interviewed and examined by the principal investigator who invited those meeting inclusion and exclusion criteria to enroll in the study.  To reduce the risk of adverse psychoactive effects in naive individuals, previous cannabis exposure was re- quired of all participants  All participants were required to refrain from smoking cannabis or taking oral synthetic delta-9-THC medications (ie, Marinol; Solvay Pharmaceu- ticals, Inc., Marietta, GA) for 30 days before study ses- sions to reduce residual effects; each participant under- went urine toxicology screening to confirm this provision  Participants were scheduled for 3, 6-hour experimental sessions at the UC Davis/Sacramento VA Medical Center General Clinical Research Center (GCRC). The sessions were sepa- rated by at least 3 days to permit the metabolic break- down of residual cannabis. The intervals between ses- sions ranged from 3 to 21 days, with a mean (SD) of 7.8 (3.4) days. Participants received either low-dose, high- cigarettes at each visit in a crossover design  After completing all 3 study sessions, participants were debriefed and paid a modest stipend (prorated at $25 per hour) for their participation. |
| **Outcomes** | Primary measure outcomes:  Spontaneous pain relief, on a 100-mm visual analog scale (VAS) between 0 (no pain) and 100 (worst possible pain).  Secondary measure outcomes:  The Neuropathic Pain Scale,28 an 11-point box ordinal scale with several pain descriptors, was a secondary outcome.  Pain unpleasantness, global impression of change, neuropathic pain scale, allodynia, quantitative sensory testing score, mood, subjective, and psychoactive effects, and neuropsychological tests) |
| **Notes** |  |

Risk of bias table

| **Bias** | **Authors' judgement** | **Support for judgement** |
| --- | --- | --- |
| Random sequence generation (selection bias) | Low risk | Crossover design using a Web-based random number– generating program, ”Research Randomizer” (http:// www.randomizer.org/). Each patient received each treatment once, in random order. |
| Allocation concealment (selection bias) | Low risk | The allocation schedule was kept in the pharmacy and concealed from other study personnel. |
| Blinding of participants and personnel (performance bias) | Low risk | Patients and assessors were blinded to group assignments |
| Blinding of outcome assessment (detection bias) | Low risk | Patients and assessors were blinded to group assignments |
| Incomplete outcome data (attrition bias) | Unclear risk | There was insufficient information to assess whether missing data were likely to induce bias on the results. |
| Selective reporting (reporting bias) | Unclear risk | No protocol was published and the outcome pain assessment on VAS or NRS and serious adverse events were not reported on. |
| Other bias | Low risk | Supported by grant C01-DA-114 from the University of California Center for Medicinal Cannabis Research. |

***Wissel 2004***

| **Methods** | A double-blind, randomised, placebo-controlled crossover study with a total duration of 9 weeks. |
| --- | --- |
| **Participants** | For inclusion they had to suffer from disabling spasticity-related pain refractory to previous pain treatment |
| **Interventions** | Nabilone or matching placebo (supplied by AOP Orphan, Great Britain) were given over a period of 4 weeks as capsules of identical colour and taste (first week 0.5 mg Nabilone per day, three weeks 1 mg Nabilone per day or matching placebo capsule). |
| **Outcomes** | Nabilone or matching placebo (supplied by AOP Orphan, Great Britain) were given over a period of 4 weeks as capsules of identical colour and taste (first week 0.5 mg Nabilone per day, three weeks 1 mg Nabilone per day or matching placebo capsule). |
| **Notes** |  |

Risk of bias table

| **Bias** | **Authors' judgement** | **Support for judgement** |
| --- | --- | --- |
| Random sequence generation (selection bias) | Unclear risk | Not described clearly |
| Allocation concealment (selection bias) | Unclear risk | Not described clearly |
| Blinding of participants and personnel (performance bias) | Unclear risk | Not described clearly |
| Blinding of outcome assessment (detection bias) | Unclear risk | Not described clearly |
| Incomplete outcome data (attrition bias) | Unclear risk | Not described clearly |
| Selective reporting (reporting bias) | Unclear risk | If no protocol was published and the outcome pain assessment on VAS or NRS and serious adverse events were not reported on. |
| Other bias | High risk | Not described |

***Zajicek 2003a***

| **Methods** | Randomised, placebo-controlled trial |
| --- | --- |
| **Participants** | **Inclusions criteria:**  Aged 18–64 years with clinically definite or laboratory-supported multiple sclerosis who, in the opinion of the treating doctor, had had stable disease for the previous 6 months, with problematic spasticity (Ashworth score of 2 in two or more lower limb muscle groups).  Since cannabinoids can potentially affect cardiac function (reducing heart rate and blood pressure), the exclusion criterion of ischaemic heart disease and the upper age limit were imposed. Furthermore, we made every attempt to stabilise factors that affect spasticity, so any physiotherapy regimen or medication likely to affect spasticity was optimised before the study and not altered in the 30 days before start of treatment.  1. Clinically definite or laboratory supported MS aged 18-64 years inclusive  2. Significant spasticity in at least 2 lower limb muscle groups (Ashworth score of 2 or more, in two or more muscle groups, eg left foot plantar flexion & left knee & right knee flexors, etc)  3. Stable disease for previous 6 months in the opinion of the treating physician  4. Antispasticity medication and physiotherapy stabilised for the last 30 days  5. Patients may be ambulatory or not  **Exclusions criteria:**  Patients with active sources of infection, or taking medication such as beta interferon, which could affect spasticity, were excluded, and we asked individuals not to have any immunisations associated with foreign travel over the 15 weeks of the study. Patients were also asked not to drive while they were receiving medication and, if unable to comply, were excluded. Other exclusion criteria included fixed-tendon contractures, severe cognitive impairment, past history of psychotic illness, major illness in another body area, pregnancy, use of 9-THC at any time, and use of cannabis in the 30 days before the start of the study.  1. Immunosuppression, including corticosteroids or interferon taken currently or in previous 30 days.  2. Past or present history of ischaemic heart disease or psychotic illness  3. Other serious illness likely to interfere with study assessment such as major organ failure, neoplasia, coeliac disease - see appendix 9 and if in doubt please contact the Plymouth Trial Coordinating Centre (PTCC) .  4. Open/ infected pressure sores or other source of chronic infection.  5. Significant fixed tendon contractures.  6. Severe cognitive impairment such that patient is unable to provide informed consent.  7. Women who are pregnant, lactating or not using adequate contraception.  8. Unwilling to stop driving or operating dangerous machinery for the study period and one week afterwards.  9. Cannabinoids taken currently or in previous 30 days.  10. Previous use of THC (Marinol) at any time.  11. Anticipated foreign travel within the first 15 weeks of the trial.  12. Anticipated immunisations within the first 15 weeks of the trial.  13. Participation in other research studies currently or within previous 3 months.  14. Other problems likely to make participation difficult at the discretion of the neurologist. |
| **Interventions** | Patients will be randomly assigned to one of four regimens in the ratio 2:1:2:1, as follows:  1. THC (Marinol) (maximum total daily dose 0.25 mg/kg in equal doses, given as 2.5 mg THC capsules)  2. Placebo capsules (containing oil vehicle) matched to appearance of THC |
| **Outcomes** | The primary outcome:  1) mean change from baseline in Ashworth score, was then compared, using analysis of variance, with treatments as fixed effects |
| **Notes** |  |

Risk of bias table

| **Bias** | **Authors' judgement** | **Support for judgement** |
| --- | --- | --- |
| Random sequence generation (selection bias) | Low risk | The coordinating centre allocated the patient a trial number and then forwarded relevant details to the central trial pharmacy, where randomisation took place, using a dedicated stand-alone computer. |
| Allocation concealment (selection bias) | Low risk | Throughout the study, the list of treatment allocation codes was kept at the central trial pharmacy, located separately from the coordinating office. |
| Blinding of participants and personnel (performance bias) | Low risk | The study coordinating team, all investigators, the data monitoring committee, and patients were unaware of the treatment allocation for the duration of the study. |
| Blinding of outcome assessment (detection bias) | Low risk | The study coordinating team, all investigators, the data monitoring committee, and patients were unaware of the treatment allocation for the duration of the study. |
| Incomplete outcome data (attrition bias) | Low risk | Data analysis was by intention to treat and was undertaken in accordance with an analysis plan drawn up and agreed by the trial steering committee before unblinding. |
| Selective reporting (reporting bias) | Low risk | A protocol was published before or at the time the trial was begun, and the outcomes specified in the protocol were reported on |
| Other bias | Low risk | The sponsors of the study had no role in study design, data collection, data analysis, data interpretation, or writing of the report.  This study was sponsored by the Medical Research Council and was supported by the Multiple Sclerosis Society of Great Britain and Northern Ireland. |

***Zajicek 2003b***

| **Methods** | Randomised, placebo-controlled trial |
| --- | --- |
| **Participants** | **Inclusions criteria:**  Aged 18–64 years with clinically definite or laboratory-supported multiple sclerosis who, in the opinion of the treating doctor, had had stable disease for the previous 6 months, with problematic spasticity (Ashworth score of 2 in two or more lower limb muscle groups).  Since cannabinoids can potentially affect cardiac function (reducing heart rate and blood pressure), the exclusion criterion of ischaemic heart disease and the upper age limit were imposed. Furthermore, we made every attempt to stabilise factors that affect spasticity, so any physiotherapy regimen or medication likely to affect spasticity was optimised before the study and not altered in the 30 days before start of treatment.  1. Clinically definite or laboratory supported MS aged 18-64 years inclusive  2. Significant spasticity in at least 2 lower limb muscle groups (Ashworth score of 2 or more, in two or more muscle groups, eg left foot plantar flexion & left knee & right knee flexors, etc)  3. Stable disease for previous 6 months in the opinion of the treating physician  4. Antispasticity medication and physiotherapy stabilised for the last 30 days  5. Patients may be ambulatory or not  **Exclusions criteria:**  Patients with active sources of infection, or taking medication such as beta interferon, which could affect spasticity, were excluded, and we asked individuals not to have any immunisations associated with foreign travel over the 15 weeks of the study. Patients were also asked not to drive while they were receiving medication and, if unable to comply, were excluded. Other exclusion criteria included fixed-tendon contractures, severe cognitive impairment, past history of psychotic illness, major illness in another body area, pregnancy, use of 9-THC at any time, and use of cannabis in the 30 days before the start of the study.  1. Immunosuppression, including corticosteroids or interferon taken currently or in previous 30 days.  2. Past or present history of ischaemic heart disease or psychotic illness  3. Other serious illness likely to interfere with study assessment such as major organ failure, neoplasia, coeliac disease - see appendix 9 and if in doubt please contact the Plymouth Trial Coordinating Centre (PTCC) .  4. Open/ infected pressure sores or other source of chronic infection.  5. Significant fixed tendon contractures.  6. Severe cognitive impairment such that patient is unable to provide informed consent.  7. Women who are pregnant, lactating or not using adequate contraception.  8. Unwilling to stop driving or operating dangerous machinery for the study period and one week afterwards.  9. Cannabinoids taken currently or in previous 30 days.  10. Previous use of THC (Marinol) at any time.  11. Anticipated foreign travel within the first 15 weeks of the trial.  12. Anticipated immunisations within the first 15 weeks of the trial.  13. Participation in other research studies currently or within previous 3 months.  14. Other problems likely to make participation difficult at the discretion of the neurologist |
| **Interventions** | 1. Natural cannabis oil (Cannador) containing the same dose of THC, made up to GMP standard  2. Placebo capsules (containing oil vehicle) matched to appearance of the cannabis capsules |
| **Outcomes** | The primary outcome:  1) mean change from baseline in Ashworth score, was then compared, using analysis of variance, with treatments as fixed effects |
| **Notes** |  |

Risk of bias table

| **Bias** | **Authors' judgement** | **Support for judgement** |
| --- | --- | --- |
| Random sequence generation (selection bias) | Low risk | The coordinating centre allocated the patient a trial number and then forwarded relevant details to the central trial pharmacy, where randomisation took place, using a dedicated stand-alone computer. |
| Allocation concealment (selection bias) | Low risk | Throughout the study, the list of treatment allocation codes was kept at the central trial pharmacy, located separately from the coordinating office. |
| Blinding of participants and personnel (performance bias) | Low risk | The study coordinating team, all investigators, the data monitoring committee, and patients were unaware of the treatment allocation for the duration of the study. |
| Blinding of outcome assessment (detection bias) | Low risk | The study coordinating team, all investigators, the data monitoring committee, and patients were unaware of the treatment allocation for the duration of the study. |
| Incomplete outcome data (attrition bias) | Low risk | Data analysis was by intention to treat and was undertaken in accordance with an analysis plan drawn up and agreed by the trial steering committee before unblinding. |
| Selective reporting (reporting bias) | Low risk | A protocol was published before or at the time the trial was begun, and the outcomes specified in the protocol were reported on |
| Other bias | Low risk | The sponsors of the study had no role in study design, data collection, data analysis, data interpretation, or writing of the report.  This study was sponsored by the Medical Research Council and was supported by the Multiple Sclerosis Society of Great Britain and Northern Ireland. |

***Zajicek 2012***

| **Methods** | Randomised placebo-controlled, double-blinded parallel-group trial |
| --- | --- |
| **Participants** | **Inclusion Criteria**  1) Troublesome muscle stiffness for at least 3 months.  2) Stable disease for the previous 6 months.  3) Antispasticity medication and physiotherapy stabilised for the last 30 days.  4) Patients may be ambulatory or not.  5) Age 18-64.  **Exclusion Criteria:**  1) Immunosuppressants which may affect spasticity (including corticosteroids and interferon but ex-cluding azathioprine) taken currently or in previous 30 days  2) Past or present history of psychotic illness.  3) Open/infected pressure sores or other source of chronic infection.  4) Significant fixed tendon contractures.  5) Severe cognitive impairment such that the patient is unable to provide informed consent.  6) History of clinically important renal, cardiovascular or neurol. diseases (apart from MS).  7) Malignancy within the past 2 years.  8) Cannabinoids taken currently or in previous 30 days.  9) Positive qualitative urinary test on cannabinoids at screening visit. (In this case a patient will be allowed to repeat the test at a second screening visit later.)  10) Known hypersensitivity to cannabinoids.  11) Current drug abuse, including alcohol abuse.  12) Laboratory parameters outside the following limits:  Creatinine > 3x upper limit of normal Bilirubine > 3x upper limit of normal Transaminases > 5 x upper limit of normal  13) Anticipated immunisations within the 12 weeks of trial participation.  14) Other problems likely to make participation difficult at the discretion of the neurologist.  15) Women who are pregnant, lactating or not using adequate contraception.  16) Participation in other treatment studies currently or within the previous month. |
| **Interventions** | The study consisted of a pretreatment screening period of 1e2 weeks, a 2 week dose titration phase and a 10 week maintenance phase. Total treatment duration was 12 weeks. Participants were assessed at 2, 4, 8 and 12 weeks after the start of treatment. |
| **Outcomes** | Primary Outcome Measures :  1) Change in muscle stiffness: 11-point numerical Likert scale  Secondary Outcome Measures :  1) Change in pain: 11-point numerical Likert scale |
| **Notes** |  |

Risk of bias table

| **Bias** | **Authors' judgement** | **Support for judgement** |
| --- | --- | --- |
| Random sequence generation (selection bias) | Low risk | Computer generated permuted block randomisation was used, stratified by centre, ambulatory status (able to walk or not) and concurrent use of antispasticity medication (yes or no). |
| Allocation concealment (selection bias) | Low risk | Participants were evenly allocated to CE or placebo by means of an interactive voice response system. |
| Blinding of participants and personnel (performance bias) | Low risk | The study coordinating team, all investigators and participants were blinded to treatment allocation throughout. |
| Blinding of outcome assessment (detection bias) | Low risk | The study coordinating team, all investigators and participants were blinded to treatment allocation throughout. |
| Incomplete outcome data (attrition bias) | High risk | For the efficacy analysis, missing data were replaced by the last observation carried forward approach. In addition, as a sensitivity assessment of the primary efficacy parameter, worst case and multiple imputation substitutions were performed. |
| Selective reporting (reporting bias) | Unclear risk | Protocol was published before or at the time the trial was begun, and the outcomes specified in the protocol were reported on |
| Other bias | Low risk | Funded by the Society for Clinical Research, Berlin, Germany, and Weleda AG, Arlesheim, Switzerland. |
