# Supplementary material for: Cannabinoids versus placebo for pain: A systematic review with meta-analysis and Trial Sequential Analysis
Source: PLoS One. 2023 Jan 30;18(1):e0267420. doi: 10.1371/journal.pone.0267420 (PMC9886264; doi:10.1371/journal.pone.0267420)
Supplement: Attachment — Submitted filename: Response to Reviewers.docx [file pone.0267420.s006.docx]

When submitting your revision, we need you to address these additional requirements.

1. Please ensure that your manuscript meets PLOS ONE's style requirements, including those for file naming. The PLOS ONE style templates can be found at
   https://journals.plos.org/plosone/s/file?id=wjVg/PLOSOne_formatting_sample_main_body.pdf and 
   <https://journals.plos.org/plosone/s/file?id=ba62/PLOSOne_formatting_sample_title_authors_affiliations.pdf>.

This has been addressed. We have changed the reference style. The headings have been changed according to the PLOS ONE requirements. Figure and table caption have been inserted directly after they have been cited in the text.

2. Please ensure that you refer to Figures 2-5 in your text as, if accepted, production will need this reference to link the reader to the figure.

Figures 2-5 are now referred to in the text.

3. Please include your tables as part of your main manuscript and remove the individual files. Please note that supplementary tables (should remain/ be uploaded) as separate "supporting information" files.

We have now included the “Table 1: Table of included studies” in our manuscript.

4. Please include captions for your Supporting Information files at the end of your manuscript, and update any in-text citations to match accordingly. Please see our Supporting Information guidelines for more information: http://journals.plos.org/plosone/s/supporting-information.

We have now included a paragraph of our supporting information files at the end of the manuscript. This includes captions of all the supplemental files.

5.  We noticed you have some minor occurrence of overlapping text with the following previous publication(s), which needs to be addressed:

- <https://bmjopen.bmj.com/content/9/10/e031574>

We have now rephrased and changes the sentence structure of the introduction section in order to avoid overlapping text with the above mentioned publication.

In your revision ensure you cite all your sources (including your own works), and quote or rephrase any duplicated text outside the methods section. Further consideration is dependent on these concerns being addressed.

We have carefully checked that all sources are cited and that there is no duplicated text outside the methods section.

1.Don’t use short forms like TSA directly in the abstract.

We have changed this accordingly

2. Introduction: Please improve upon the Introduction. There should be use of a variety of sentence structures, if possible. Importantly, the gaps in current literature and consequently the rationale for this review remain unclear and should be elaborated upon further in the Introduction. Secondly, please discuss both acute and chronic pain while briefly outlining the comparison of cannabinoids with other relevant treatment therapies for pain. Thirdly, briefly outline the please discuss current guideline recommendations, if any. You may find the following paper useful: <https://www.cadth.ca/sites/default/files/pdf/htis/2019/RC1153%20Cannabis%20Chronic%20Pain%20Final.pdf>

The introduction has now been revised and separated into different sections to provide a better overview. We have tried to describe the difference between acute pain, chronic pain and cancer pain more in depth while trying to included current guideline recommendations. We thank the reviewer for this suggestion.

We have also tried to clarify the rational for this systematic review further in the end of the introduction section.

3. Methods: Please cite “visual analogue scale (VAS) or numerical rating scale (NRS)”. Additionally, please elaborate upon the use of 95% and 98% confidence intervals for dichotomous outcomes – “We calculated risk ratios (RRs) with 95% and 98% confidence intervals (CIs) for our dichotomous outcomes”.

We have now cited the visual analogue scale (VAS) or numerical rating scale (NRS) in the methods section.

We have also elaborated upon the use of two different confidence intervals. This rationale has also been described in the section “Assessment of statistical and clinical significance" previously, therefore we have also referred to this section.

4. Results: The detailed analysis of the data at hand is commendable. Please state an insignificant p value as there being no difference between the cannabinoid and placebo groups, for instance for “Meta-analysis indicated that cannabinoids increased the risk of serious adverse events (RR 1.18; 98% CI 0.95 to 1.45; P = 0.07; low certainty of evidence; Supplementary figure 27).”

We have changed this to “Meta-analysis showed no evidence of a difference between cannabinoids versus placebo (RR 1.18; 98% CI 0.95 to 1.45; P = 0.07; low certainty of evidence; Fig 3, Supplementary figure 27)”.

5. Use the short form for “Trial Sequential Analysis” as TSA after mentioning the full-form once initially. Also, state the full form of GRADE.

We have changed this accordingly.

6. Discussion: Please discuss, compare, and contrast findings of other studies with your findings instead of merely stating “Our findings are in contrast to the majority of previous reviews which have indicated an adequate analgesic effect of cannabinoids and supported the use of cannabinoids for the treatment of chronic pain. 20-23 111 112 Our findings suggest that cannabinoids reduced chronic pain when compared with placebo, however, whether this effect is clinically important seems questionable. Our findings regarding cancer pain and acute pain are in line with previous reviews on this topic.113- 115”. Also, improve upon the structure and quality of the Discussion section in general.

We have included the position paper of the IASP and compared our results to the findings of the IASP. Further, we have divided the discussion section into several parts to improve the structure. Therefore the following sections appear in the discussion section: “Strengths”, “Limitations” and “Minimal important difference”.

7. References: Avoid the use of references more than 10 years old.

We thank the peer reviewer for this advice. This has been taken into consideration.
